# Hierarchy of human IgG recognition within the *Staphylococcus aureus* immunome

**DOI:** 10.1038/s41598-018-31424-3

**Published:** 2018-09-05

**Authors:** Emily E. Radke, Stuart M. Brown, Adam J. Pelzek, Yi Fulmer, David N. Hernandez, Victor J. Torres, Isaac P. Thomsen, William K. Chiang, Andy O. Miller, Bo Shopsin, Gregg J. Silverman

**Affiliations:** 1 New York University School of Medicine, Department of Medicine, New York, 10016 USA; 2 New York University School of Medicine, Department of Microbiology, New York, 10016 USA; 3 Vanderbilt University Medical Center, Department of Pediatrics, Division of Infectious Diseases, Nashville, 37232 USA; 4 Hospital for Special Surgery, New York, 10021 USA

## Abstract

*Staphylococcus aureus* is an opportunistic pathogen that causes a range of serious infections associated with significant morbidity, by strains increasingly resistant to antibiotics. However, to date all candidate vaccines have failed to induce protective immune responses in humans. We need a more comprehensive understanding of the antigenic targets important in the context of human infection. To investigate infection-associated immune responses, patients were sampled at initial presentation and during convalescence from three types of clinical infection; skin and soft tissue infection (SSTI), prosthetic joint infection (PJI) and pediatric hematogenous osteomyelitis (PHO). Reactivity of serum IgG was tested with an array of recombinant proteins, representing over 2,652 *in-vitro*-translated open reading frames (ORFs) from a community-acquired methicillin-resistant *S*. *aureus* USA300 strain. High-level reactivity was demonstrated for 104 proteins with serum IgG in all patient samples. Overall, high-level IgG-reactivity was most commonly directed against a subset of secreted proteins. Although based on limited surveys, we found subsets of *S*. *aureus* proteins with differential reactivity with serum samples from patients with different clinical syndromes. Together, our studies have revealed a hierarchy within the diverse proteins of the *S*. *aureus* “immunome”, which will help to advance efforts to develop protective immunotherapeutic agents.

## Introduction

*Staphylococcus aureus* is an opportunistic bacterial pathogen that causes a range of serious infections associated with significant morbidity, with 10,000 deaths per year in the US alone^1–3^. *S*. *aureus* is also a common commensal microorganism, chronically colonizing approximately 30 percent of adults, with the remainder intermittently colonized^4,5^. Antibiotic resistant strains, including methicillin-resistant *S*. *aureus* (MRSA), have become more prevalent, especially in community settings^6^, and increasing resistance to other commonly used antibiotic treatments has also been documented^7,8^. Mobile genetic elements enable efficient horizontal transfer of antibiotic resistance genes and other virulence factors^9,10^, resulting in the rapid genetic diversification of *S*. *aureus* strains^11^. Due to this genomic plasticity^12^, coupled with the slow development of new antibiotics for clinical use, there has been greatly increased interest in the development of vaccines and therapeutic immunotherapies directed against *S*. *aureus*.

The need for an effective vaccine is well recognized, and thus a range of *S*. *aureus* surface antigens, as well as whole organism preparations, have been evaluated^13–15^. Yet despite extensive efforts to develop vaccines against *S*. *aureus*, a broadly protective clinical vaccine has not been validated in controlled clinical trials^16^, as none has imparted protective immunity from serious infections, while in some cases vaccination has led to worse outcomes^17^. In part, this may be due to our current incomplete understanding of host-commensal/pathogen relationships and the range of *S*. *aureus* products that can represent antigenic targets for host immune defenses.

Candidate vaccines for *S*. *aureus* are generally first validated in mouse models, before a clinical trial is even considered. Yet murine models of infection display important differences from human infections, and therefore may have inherent issues for the identification of key features of human immunity^18^. Indeed, the set of immunodominant antigens recognized in mouse infection may not accurately identify antigens critical for controlling human infections^19^. Interpretation of animal model data is further complicated by evidence that chronic carrier states and recurrent *S*. *aureus* infections are common in humans, and these exposures do not induce immune responses sufficient to protect from subsequent infections^20^. Furthermore, protection from specific types of clinical infection syndromes may require antibody responses to different sets of staphylococcal antigens. Thus, it is essential that we perform more complete interrogations of immune responses in patients with different clinical syndromes.

To help guide the development of effective immune protective and therapeutic agents, we sought to perform unbiased surveys of human immune responsiveness to all potential protein antigens encoded by the genome of epidemic community-acquired MRSA (CA-MRSA) strain USA300. Our goal was to better understand which *S*. *aureus* proteins are recognized during human infection, as well as those which are rarely or never recognized by our immune systems. We postulated that the results from these simple surveys would in part provide an essential step in the assembly of an effective combinatorial vaccine.

*S*. *aureus* can cause different clinical infection syndromes, which in part may result from expression of different virulence factors by the infecting strain^21–23^. To identify whether infections commonly lead to antibody responses to different sets of *S*. *aureus* proteins, we used serum samples, collected at acute and convalescent time points, from representative patients with: adult skin and soft tissue infection (SSTI), adult prosthetic joint infection (PJI), and pediatric hematogenous osteomyelitis (PHO). To investigate which open reading frames (ORFs) can encode for immunogenic proteins, we used solid-phase arrays printed with *in vitro*-translated recombinant proteins from every ORF from a prototypic clinical MRSA strain. This view of the reactivity across all ORFs is enhanced by the use of longitudinal samples from human patients with a variety of clinical syndromes, providing a novel and comprehensive view of what the immune system is recognizing from *S*. *aureus*. From these unbiased investigations, we identified a hierarchy of antigenic proteins from *S*. *aureus*, which are recognized by the immune systems of individuals recovering from *S*. *aureus* infections.

## Results

### Selection of representative patients with serum antibody responses against common *S*. *aureus* antigens

To investigate patterns of immune responsiveness to the *S*. *aureus* immunome, we recruited a total of 95 patients with *S*. *aureus* infection from three cohorts; adults with SSTI (n = 55) or PJI (n = 12), and a pediatric cohort (n = 28) with hematogenous osteomyelitis. For each patient in the study, we also recovered the infecting *S*. *aureus* isolate, and screened for colonization of the nares and groin.

For the initial characterization of patient immune responses, serum samples from initial clinical presentation and follow-up visits were used to quantitate IgG reactivity with 46 recombinant antigens, including *S*. *aureus* proteins and control antigens (Supplementary Table S1). We prioritized male patients from each of the three clinical cohorts for further study based on detection of increased IgG-reactivity with three or more *S*. *aureus* antigens in the short-term follow-up blood sample compared to the baseline sample (Supplementary Fig. S1). These representative patients ranged from 9 to 63 years of age (median 39 years). Of the seven selected patients, four were from the SSTI cohort, one from the PJI cohort, and two from the PHO cohort (Table 1).

**Table 1:** Patients selected for *S. aureus* protein array analysis.

Patient ID	Age	Gender	*S*. *aureus* cohort	Other medical conditions	Prior *S*. *aureus* infection^	Prior antibiotic course	Days Infected Prior to Visit 1*	Days from Visit 1 to Visit 2	Days from Visit 1 to Visit 3
SSTI 1	54	Male	SSTI	Seizures, HCV	No	No	3	37	178
SSTI 2	25	Male	SSTI	None	No	Yes	5	48	195
SSTI 3	61	Male	SSTI	HCV, pulmonary fibrosis, hypothyroidism	No	No	3	39	207
SSTI 4	50	Male	SSTI	None	No	No	4	41	173
PJI 1	63	Male	Prosthetic Joint (PJI)	Peripheral vascular disease, chronic liver failure, ulcerative colitis, alcoholic cirrhosis, recurrent *C*. *difficile*	N/A	N/A	N/A	45	183
PHO 1	9	Male	Septic Arthritis (PHO)	Eczema	N/A	N/A	N/A	27	190
PHO 2	11	Male	Osteomylitis (PHO)	None	N/A	N/A	N/A	41	223

Patient identification numbers, as well as their age, gender, *S. aureus* proven syndrome, any co-morbid medical conditions are included for all patients selected for the protein array, in addition to the number of days between acute clinical presentation and both the short-term and long-term follow-up visits. Patients in the SSTI cohort provided additional information such as prior and subsequent *S. aureus* infections or antibiotic courses prior to enrollment, as well as patient-reported duration of symptoms that preceded the day of enrollment. N/A: Data not available. ^This designation was assigned based on the patient’s statement that there was no history provided of an infection requiring attention from a healthcare professional, and a course of antibiotics. *Patient reported, days with symptoms prior to hospital visit.

### Differential serum IgG reactivity within the *S*. *aureus* proteome

We next sought to characterize each of the patient serum samples for the presence of IgG-antibodies against *S*. *aureus* antigens in our solid-phase printed immunome arrays. Each array included 2,652 ORF-encoded recombinant proteins from epidemic CA-MRSA strain USA300, and each ORF product was individually ranked based on mean reactivity with IgG across a total of 21 patient visit serum samples (Supplementary Fig. S2). An ORF-encoded protein was classified as immune reactive if the associated IgG signal was at least two-fold above the background level of control spot fluorescence on an array, as described^24,25^. Whereas most ORF protein products were devoid of significant IgG-reactivity with any of the samples tested (Supplementary Table S2), 1,086 of these 2,652 polypeptides instead displayed IgG-reactivity with one or more serum sample (Supplementary Table S3).

**Figure 1:**
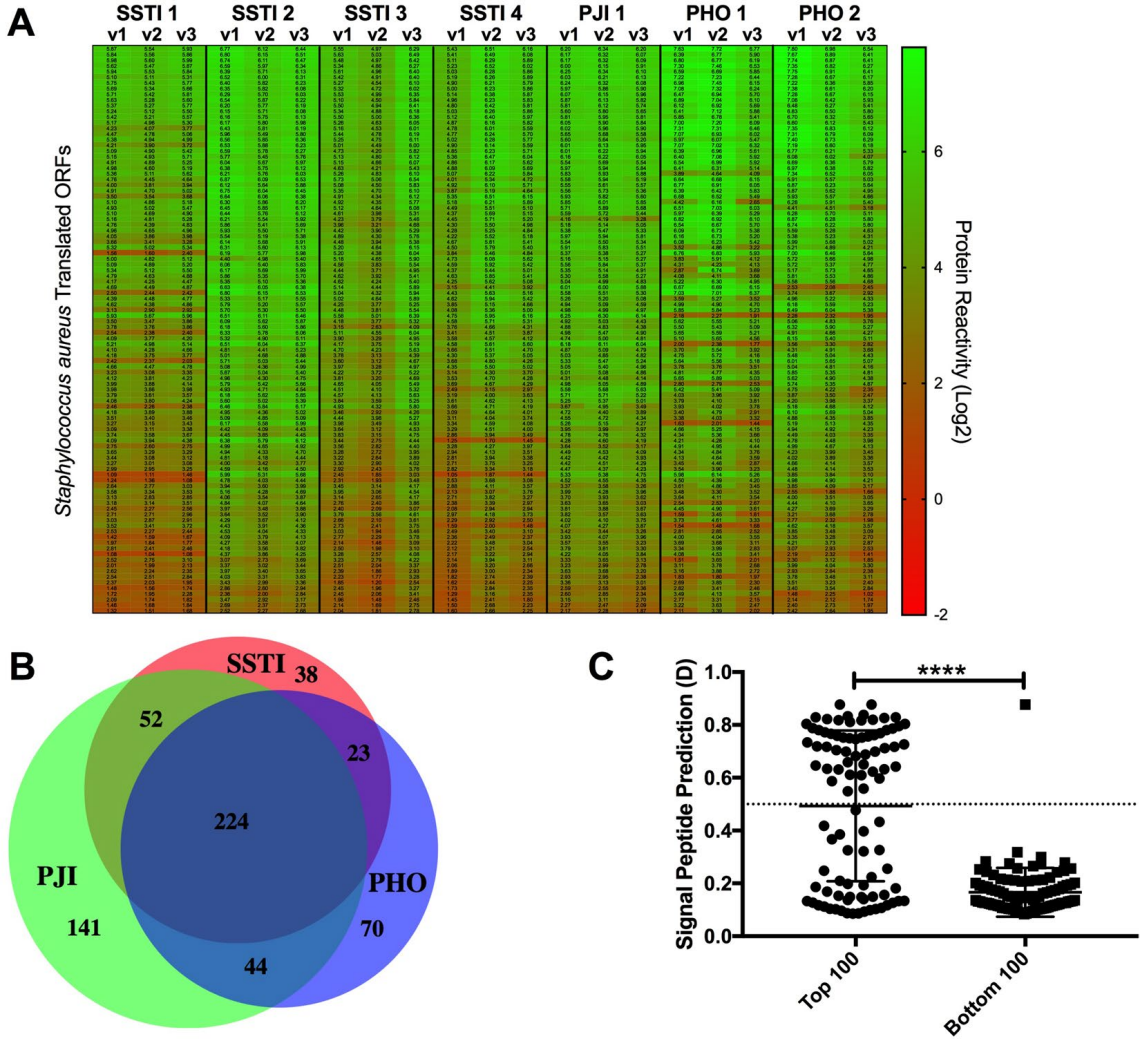
Hierarchy of reactivity for human serum IgG to antigens from *S*. *aureus* USA300 MRSA in different clinical infection types. Relative IgG binding reactivity was measured using a chip-based method for 2,652 ORFs in the *S*. *aureus* FPR3757 reference genome for seven patients, with SSTI (n = 4), PJI (n = 1), and PHO (n = 2) at three different time points of infection (acute, short term follow-up, and long-term follow-up). **(A)** Proteins were ranked based on overall mean IgG-antibody reactivity within patient serum samples. The 100 proteins with the top IgG reactivity proteins with each of the 21 serum samples are depicted. **(B)** The mean antibody reactivity value for each infection group (SSTI, PJI and PHO) across all the samples was determined, and antigens with mean reactivity of 1.0 or greater (base 2) were included in the analysis. **(C)** ORF sequences from the top and bottom 100 antigens identified in the protein array were analyzed with the SignalP Server and the Signal Peptide Prediction value, termed D, was obtained. A cutoff of D = 0.5 or greater predicted there was a signal peptide associated with the particular ORF. The top and bottom 100 IgG-reactive ORF-encoded antigens displayed significant differences (p < 0.0001) in the representation of predicted signal peptides.

To identify the most immune-reactive *S*. *aureus* proteins, we ranked all of the ORF products by their relative IgG reactivity with all of the serum samples, with reactivity shown for the top 100 shown in Fig. 1A and listed in Supplementary Table S4, and the top 50 most IgG-reactive proteins (Table 2). Although there was some variability in reactivity between patients, we found more limited differences in samples obtained at different time points from in an individual (Fig. 1A). We thereby found an overall hierarchy amongst these ORFS, with a defined subset that were IgG-reactive across each of the different subjects from the three infection types that were evaluated.

**Table 2:** 50 most IgG-reactive *S. aureus* antigen by protein immunoarray.

No.	Locus Tag	Protein Name/Description	Signal Peptide	Transmembrane
1	SAUSA300-0113	immunoglobulin G binding protein A (SpA)	+	+
2	SAUSA300-0398	superantigen-like protein SSL4	+	−
3	SAUSA300-0403	superantigen-like protein SSL9	+	−
4	SAUSA300-2366	gamma-hemolysin component C (hlgC)	+	−
5	SAUSA300-2579	N-acetylmuramoyl-L-alanine amidase domain protein	+	−
6	SAUSA300-0883	MAP domain-containing extracellular adherence protein Eap	+	−
7	SAUSA300-2364	IgG-binding protein SBI (sbi)	+	−
8	SAUSA300-1029	Iron-regulated surface determinant Protein A (IsdA)	+	+
9	SAUSA300-1028	Iron-regulated surface determinant Protein B (IsdB)	+	+
10	SAUSA300-1920	chemotaxis-inhibiting protein CHIPS (chs)	+	−
11	SAUSA300-1382	Panton-Valentine leukocidin, LukS-PV (lukS-PV)	+	−
12	SAUSA300-2367	gamma-hemolysin component B (hlgB)	+	−
13	SAUSA300-1768	leukotoxin LukD (lukD)	+	−
14	SAUSA300-1917	map protein, programmed frameshift (map)	+	−
15	SAUSA300-0651	peptidase M23	+	−
16	SAUSA300-0951	V8 protease (sspA) [3.4.21.19]	+	−
17	SAUSA300-0408	heme uptake protein	+	−
18	SAUSA300-0438	CHAP domain family	+	−
19	SAUSA300-0409	peroxidase inhibitor	+	+
20	SAUSA300-0693	electron transfer DM13	+	−
21	SAUSA300-0214	Sugar phosphate isomerase/epimerase PFAM family PF07582	−	−
22	SAUSA300-2506	immunodominant staphylococcal antigen A precursor (isaA)	+	−
23	SAUSA300-1058	alpha-hemolysin precursor (HLA)	+	−
24	SAUSA300-0548-s2	sdrE protein (sdrE)	+	+
25	SAUSA300-0776	thermonuclease precursor (nuc) [3.1.31.1]	+	+
26	SAUSA300-0547-s2	sdrD protein (sdrD)	+	+
27	SAUSA300-2572	zinc metalloproteinase aureolysin (aur) [3.4.24.29]	+	−
28	SAUSA300-0370	SEP staphylococcal enterotoxin	−	−
29	SAUSA300-1030	iron transport associated domain protein	+	+
30	SAUSA300-1476	acetyl-CoA carboxylase, biotin carboxyl carrier protein (accB)	−	−
31	SAUSA300-1922	staphylokinase precursor (sak)	+	+
32	SAUSA300-0862	glycerophosphoryl diester phosphodiesterase (glpQ) [3.1.4.46]	+	−
33	SAUSA300-1055	fibrinogen-binding protein (efb)	+	−
34	SAUSA300-1481	hypothetical mating channel protein	+	+
35	SAUSA300-0955-s1	autolysin (atl) [3.5.1.28]	+	−
36	SAUSA300-0774	secretory extracellular matrix, plasma binding protein (empbp)	+	−
37	SAUSA300-0950	cysteine protease precursor (sspB) [3.4.22.48]	+	+
38	SAUSA300-1381	Panton-Valentine leukocidin, LukF-PV (lukF-PV)	+	−
39	SAUSA300-2441	fibronectin binding protein A (fnbA)	+	−
40	SAUSA300-2136	iron compound ABC transporter, iron compound-binding protein	+	−
41	SAUSA300-2440	fibronectin binding protein B (fnbB)	+	−
42	SAUSA300-0242	sorbitol dehydrogenase (gutB) [1.1.1.14]	−	−
43	SAUSA300-1985	serine-aspartate repeat family protein, SdrH (sdrH)	+	+
44	SAUSA300-0279	membrane protein YhgE, type VII secretion protein EsaA)	−	+
45	SAUSA300-0708	histidinol-phosphate aminotransferase (hisC) [2.6.1.9]	−	−
46	SAUSA300-0399	superantigen-like protein SSL5	+	−
47	SAUSA300-0955-s2	autolysin (atl) [3.5.1.28]	+	−
48	SAUSA300-0395	superantigen-like protein SSL1	+	−
49	SAUSA300-1031	heme ABC transporter permease	−	+
50	SAUSA300-1512	penicillin-binding protein 3 (pbp3)	−	+

IgG reactivity for each antigen was determined across all patients and samples, with ranking determined by overall mean reactivity, in addition to the presence of reactivity in each patient at each time point (see Methods). Numbers in brackets [] represent the enzyme commission designation (see www.genome.jp/kegg/).

### IgG reactivity correlated in part with structural domain predictions

For each infection group, we calculated the average reactivity for each individual at each time point compared to the other infection groups. An average reactivity of two-fold over background was considered a positive immune response. The specific proteins associated with each infection group were identified, and the average reactivity was estimated (Supplementary Table S5). A subset of antigens was reactive in individuals from all the three infection groups (n = 224), whereas other antigens (n = 253) were reactive with sera from only one of the three infection groups (Fig. 1B). Subsets of proteins which were the most highly IgG-reactive were identified for each of the clinical infection syndromes. Although only a limited number of patients per clinical group were studied, amongst the most highly IgG-reactive proteins we identified subsets of proteins that uniquely distributed with each of the different infection groups (Supplementary Table S5).

Although different proteins were associated with different clinical syndromes, secreted virulence factors were generally highly represented at the top of these hierarchies (Table 2). Notably, SSTI syndrome alone was associated with reactivity to a specific cell surface protein^26^, encoded by SAUSA300-1327 (Supplementary Table S5). This gene encodes for extracellular matrix-binding protein or ebh^27^, which contains many FIVAR (Found In Various Architectures) protein domains that are postulated to bind fibronectin and  N-acetyl glucosamine in the host extracellular matrix^28^. Notably, due to the large size of the predicted ORF, 16 segments were separately printed on the array, and we found that seven of these ebh polypeptides were recognized by antibodies in the serum of one or more of the SSTI patients (Supplementary Table S5).

We next analyzed the genotypes of the infecting isolates from the representative patients chosen for this study. Five out of the seven patients were infected with MRSA isolates (Supplementary Table S6), while the two PHO patients were instead infected with MSSA. We also examined the phylogenetic relationship between the patient infecting *S*. *aureus* isolates and the reference strain, FPR3575, used in the array (Supplementary Fig. S3). Curiously, based on our genome analyses the isolates from each infection group did not cluster together. We then compared the ORF sequence homology of the 100 top IgG-reactive proteins in each of the 7 infecting strains and the reference USA300 strain used in the array by BLAST identity (Supplementary Fig. S4). The results indicate that the isolates are polymorphic, and among the top 100 proteins there are many cases in which an isolate either does not contain a homologous protein or contain a variant of the protein with differences in sequence. Thus, we detected sequence variations between isolates and in synonymous ORFs that could affect recognition of associated epitopes.

Among USA300 antigens that were highly reactive with sera from all 7 patients, we screened antigen sequences for the presence of a secretion signal peptide or transmembrane domains. As expected, ORFs with signal peptides were more common amongst the top 100 IgG-reactive proteins (Table 2) compared to the 100 with lowest reactivity levels (p < 0.0001, Fig. 1C). In contrast, we found that ORFs predicted to contain transmembrane domains were not differentially represented in the top and bottom IgG-reactive sets of potential antigens (Supplementary Fig. S5). The representation of motifs predictive of transmembrane domains therefore did not correlate with the level of IgG-reactivity. Furthermore, many of the genes for proteins that were non-reactive with circulating IgG (Supplementary Table S2), were associated with intracellular bacterial functions, such as ribosome-binding factor A (rbfA – SAUSA300-1163) or 30S ribosomal protein S14 (rpsN – SAUSA300-2191). Collectively, these observations suggest that antibody reactivity was primarily against secreted antigens that likely become accessible for immune recognition by the host.

Amongst the top antibody-reactive proteins, the IgG-binding proteins, Staphylococcal Protein A (SpA) and Staphylococcal binding immunoglobulin protein (Sbi) ranked first and seventh for overall reactivity, respectively. Yet due to the non-immune nature of the binding specificities of these virulence factors^29,30^ the results for these microbial products must be deemed as not informative for investigations of host immune responses.

### Concordance of IgG-antibody reactivity in solid-phase printed protein and bead-based assays

As immunoreactivity may in part be affected by technical features of the assay, we also assessed whether the level of IgG reactivity for a given *S*. *aureus* antigen in the chip-based approach correlated with the level of *S*. *aureus* expressed antigen reactivity in a multiplex bead-based assay system. Notably, the bead-based assay detected IgG reactivity in a generally broader dynamic range, and for many Leukocidin family members this enabled the detection of longitudinal increases in IgG antibody responses in the sequential samples from each of these selected patients (Supplementary Fig. S1). In general, although specific reactivity values differed, we found that secreted toxins, especially members of the Leukocidin family, were amongst the most immunogenic *S*. *aureus* antigens by both approaches (Table 2).

Finally, we compared our results to available data on the level of *S*. *aureus* gene expression during clinical infection that is no doubt an important determinant of the potential immunogenicity of individual *S*. *aureus* genes. We used data reported for *S*. *aureus* gene expression levels determined from *ex vivo* analyses of mRNA isolated from active clinical cutaneous abscesses, as reported by Date *et al*.^31^. Gene expression data from this study was normalized with levels detected in *in vitro* cultures of the same *S*. *aureus* strain (USA300) that was used as the source of proteins in our microarray. Our analyses found that during active infection 26 of our top 50 immune-reactive proteins also displayed two-fold or greater increases in gene-specific transcript expression (Supplementary Fig. S6). Interestingly, a prominent member of the Leukocidin family, LukF-PV, displayed an 89-fold increase in RNA levels in skin abscesses compared to laboratory culture^31^. Thus, antigen reactivity in our system correlated with *in vivo* bacterial gene expression during clinical infection, which strongly supported the relevance of our methodological approach for the identification of *S*. *aureus* proteins involved in host immune responses. In summary, based on recognition by host serum IgG we have identified a hierarchical set of staphylococcal antigens, and found that a limited group of these microbial products appear to be immunologically targeted in patients with a range of clinical infection syndromes (Fig. 1B).

## Discussion

Herein, we report findings from an unbiased approach to identify antigens associated with different types of *S*. *aureus* clinical infections. Using an antigen array of all ORFs in a USA300 CA-MRSA clinical strain, we measured serologic IgG reactivity against every potential staphylococcal protein and found that only a small subset of total ORFs encoded for proteins that are immunologically-recognized across all infection types. In addition, we detected IgG-binding reactivity in one or more serum sample with nearly half of the *S*. *aureus* ORF protein products; 1,028 out of the 2,652 tested, which at first glance represents an overwhelming number of potential antigenic targets (Supplementary Table S3).

Among the top IgG-reactive antigens were the subunits of Panton-Valentine Leukocidin (i.e., LukS/F-PV). The heterodimeric *S*. *aureus* antigen LukS/F has been reported to be highly associated with SSTI^21,32^. In our comparisons in disease syndromes, the genes for LukS and LukF were amongst the most up-regulated^31^ and these also appeared to be amongst the most immunogenic targets in patients with skin abscess (Supplementary Fig. S6). Notably, although IgG responses to LukS and LukF were highly up-regulated in all patients studied, only isolates from SSTI patients contained genes for the LukS/F genes (Supplementary Table S6). This could reflect persistent responses from prior exposure to a strain that produced these gene products. Alternatively, this could also reflect the IgG cross-reactivity with epitopes expressed by different members of the Leukocidin family, which reiterates recently reported findings from more focused studies of a larger number of patients^33^.

In several important instances, reactivity documented in the printed-arrays also correlated with post infection increases in antigen-reactive IgG titers demonstrated in our multiplex bead-based analysis. In particular, LukS-PV ranked at number 11 and LukF-PV, the partner subunit in the heterodimeric protein, ranked at 38 in overall IgG reactivity in these array studies (Table 2), and very similar patterns of increases in antibody reactivity over time were demonstrated in our bead-based assay (Supplementary Fig. S1). In a recent study we showed that infection can induce raised antibody responses to a number of *S*. *aureus* gene protein products, but such enhanced immune responses to infection do not generally persist long-term^33^. The technical approach used in the current report enables more complete antigenic surveys with more sensitive detection of IgG binding. By including sera from a follow-up visit at about 6-weeks that was after a course of antibiotics, we ensured the best detection of even transiently enhanced responses to staphylococcal proteins. Furthermore, proteins with the greatest IgG reactivity were amongst the most highly expressed during cutaneous infection based on gene expression data (Supplementary Fig. S6). Cumulatively these findings suggest that our immune systems often have exuberant, yet transient, responses to specific virulence factors that are associated with highly up-regulated expression during *S*. *aureus* infection of the human host.

Among the most reactive recombinant proteins identified were SpA and Sbi, which are IgG-binding proteins implicated as countermeasures that actively subvert host defenses^30,34,35^. SpA, a virulence factor that is both expressed on the bacterial surface and secreted^36^, promotes immune evasion due to its ability to bind the Fc portions of IgG antibodies, interfering with opsonophagocytic clearance. SpA also has a separate binding site for Fab encoded by VH3-family genes that convey the properties of a VH-targeted B-cell superantigen^37,38^. In our study, SpA, which was identified as the most IgG-reactive recombinant *S*. *aureus* protein in all serum samples (Table 2). Although it is unclear from these surveys whether the high reactivity comes from the binding of the Fab or Fc portions of the IgG antibodies, it provided a validating technical control for detection of IgG reactivity. Notably, Iron-regulated surface determinant Protein B (IsdB) was also identified within the top 10 IgG reactive proteins as well as the related IsdA (Table 2). In a recent report, NEAT1 and NEAT2 domains of IsdB were associated with a different type of non-immune Fab-binding interaction specific for germ-line VH gene segment CDR2 motifs that may reflect co-evolution of the human immune system with this opportunistic microbial pathogen^39^.

Many other *S*. *aureus* genes, including several in our top 100 list for IgG-reactivity, are currently annotated as hypothetical proteins of unknown structure (Table 2, Supplementary Table S3) due to current uncertainty regarding their biologic roles. However, the detection of IgG reactivity may suggest these proteins are expressed in the context of infection. Further investigations of the structure and function of “hypothetical” proteins with prominent IgG reactivity may illuminate currently obscure facets of the pathogenesis of *S*. *aureus* infection. Notably, SSTI syndrome alone was associated with reactivity for an extracellular matrix-binding protein, ebh, encoded by SAUSA300-1327 (Supplementary Table S5), which should be further considered as a candidate antigen in an experimental vaccine.

A previous report of the immunome associated with *S*. *aureus* infection assessed reactivity of serum antibodies against staphylococcal antigens using 2-D immunoproteomics and mass spectrometry, which by comparison are time and labor-intensive approaches^40^. In one recent paper, a printed array-based strategy similar to ours was used but only 44 antigens examined^41^. Other studies have used ELISA or bead-based arrays to assess responses to much more limited sets of selected *S*. *aureus* antigens^15,42–44^. Whereas Etz *et al*. described surveys of the *S*. *aureus* genome that assessed binding to short *S*. *aureus* gene product fragments displayed on bacterial cells, few potential antigens, including a coagulase, as well as SpA, were identified^45^. By comparison, our studies used a much more comprehensive array of *in vitro*-translated antigens, representing products of the entire genome of a clinical isolate of *S*. *aureus*, with a method amenable to high-throughput analyses. We thereby identified antigens that were recognized immunologically across several groups of patients with different types of clinical infection. We have also identified the large set of staphylococcal antigens that are not reactive with human serum IgG antibodies. Taken together, we have provided a comprehensive overview of the immunome during the acute phase and during the resolution of *S*. *aureus* infection.

The design of this methodological approach also involved a level of compromise as there are several inherent technical limitations. First, although our microarray studies were unbiased and comprehensive, these are based on a single reference strain (USA300, FPR3757) and therefore could not detect antigens not represented in this strain. There is such large diversity of gene composition between *S*. *aureus* strains that it would be very difficult to generate a protein array containing all potential ORFs found in any and all *S*. *aureus* strain, therefore this single reference genome was chosen. Likewise, in some cases the microarray may not have been able to detect antibody binding to antigen variants unique to other infecting strains, which may be polymorphic from the microarray reference strain (Supplementary Fig. S4). Second, in these studies of *in vitro* translated *S*. *aureus* ORFs printed onto the microarray we could not control for altered protein folding and conformation, or post-translational modifications that could affect the range of epitope expression. Third, compared to results from our bead-based multiplex assay, we found that there was a more limited dynamic range for antibody reactivity in the printed protein array studies, and binding levels were more quickly saturated for recombinant proteins encoded by the same gene sequence^33^. Therefore, we chose to use a ranking based hierarchy method, instead of using values from a semi-quantitative method that we felt would have less inherent utility. Using a ranking based method, we are able to normalize for differences between patients from overall average MFI for IgG binding. For practical reasons, for each patient and time point we used a single dilution of serum (1:2) and based on the absence of truly naïve human serum we could not otherwise normalize serum concentrations across the time points of patients and between patients. Inherent to the technical approach of the printed protein microarray, non-protein antigens were outside of the scope of our study, as this report was meant to test the potential utility of this printed array technical approach to aid in prioritizing potential protein antigens. In the future, these studies will be extended to larger patient groups and studies of ORF products from multiple strains.

We emphasize that *S*. *aureus* is an opportunistic pathogen, and a common commensal colonizer of the nares and cutaneous sites, with evidence of near universal immune exposure after infancy. We therefore focused on studies of patients with infection-associated increases in antibody responses during subsequent visits. In fact, all of the adult patients we investigated had significant IgG anti-Staphylococcal antibody reactivity at the time of recruitment, and it is therefore unknown whether such responses are from the current infection or from prior exposures. Furthermore, structurally homologous toxins expressed during the active infection could induce cross-reactive antibodies to antigens that themselves are not actively being expressed, such as the Panton-Valentine (i.e., LukS/F) exotoxin mentioned previously, which had high IgG-reactivity in the immunoassay even though we found that the gene was only present in 4 out of the 7 patients tested (Supplementary Fig. S4, Supplementary Table S6)^33^. In addition to the Leukocidin toxins, cross-reactivity has been reported within other antigenic groups like the *S*. *aureus* superantigen family^46^. While our studies focused on elucidating the reactivity of IgG antibodies with the *S*. *aureus* immunome, we are aware that other isotypes may also be important, and especially IgA antibodies that play essential roles at mucosal surfaces. Despite these caveats, our investigations were informative and shed light on the immunoreactivity patterns that were shared, or which varied, between different subjects, and in those with different clinical syndromes.

In part, we initiated these studies as past vaccine candidates have not met their primary endpoints^47,48^. The failure of past vaccine attempts could be due to the *S*. *aureus* antigens chosen for these vaccines, and that in many cases only single proteins were used. We therefore initiated the current studies because we believed it was time to take a step back and perform unbiased surveys of a more complete set of *S*. *aureus* antigens that may be recognized by serum antibodies from well-characterized patients recovering from different clinical infection syndromes.

Our studies have provided an overview of the immunologic potential of individual antigens within the *S*. *aureus* genome, which we hope will contribute to the advancement of vaccine research for this pathogen. From our findings, we postulate that the proteins associated with strong antibody immunoreactivity in all convalescent patients may be relevant to the development of a broad-reaching vaccine, whereas proteins with immunoreactivity associated with only certain infection groups may reflect features deriving from mechanisms of pathogenicity of the associated infection syndrome that at times may be linked to specific infecting strains of *S*. *aureus*. As *S*. *aureus* genomes shows great genetic diversity and plasticity, efforts such as ours to objectively characterize levels of antigenicity of the broad range of staphylococcal proteins, in the context of human infection, are critical for the development of effective clinical vaccines.

## Methods

### Patient cohort

Patients were enrolled and informed consent was obtained following IRB approval under institutional supervision at three university medical centers: SSTI and uninfected adult controls at Bellevue Hospital and NYU Tisch Hospital; Prosthetic Joint Infection (PJI) patients and uninfected controls at the Hospital for Special Surgery (HSS); and pediatric patients with hematogenous osteomyelitis (PHO) at Vanderbilt Medical Center. Blood samples and cultures were obtained from colonizing (nares, groin) and infecting sites in patients. Eligibility was confirmed by culture-based documentation of *S*. *aureus* infection by the clinical microbiology laboratory in accordance with IRB protocols. All *S*. *aureus* isolates were preserved for genomic characterization. Genotyping involved a combination of standard molecular methods including *spa* (staphylococcal protein A) and *SCCmec* typing and screening for presence of *pvl* (Supplementary Table S6)^49^. For analysis of antigen polymorphism, DNA from *S*. *aureus* isolates was extracted as described^50,51^. DNA from each patient isolate was then sequenced to a depth of approximately 150X with 150 base paired-end reads on the Illumina HiSeq4000. Adapters and low-quality bases were trimmed from the ends of reads using Trimmomatic v 0.33^52^. Draft genomes were assembled from the sequence data for each isolate using SPAdes v 3.9.0^53^. All genome assemblies have N50 > 100 Kb and (sum of contigs) genome length from 2.8 to 3.1 Mb.

At initial presentation to the hospital with symptoms patient blood samples and complete blood counts were obtained, then patients received a course of antibiotics as determined by their physicians. Further blood samples were recovered at short-term (after 27–48 days), and long-term follow-up (after 173–223 days). Among the data collected were patient reports of past exposure to *S*. *aureus* and infections, co-morbidities, and past antibiotic usage. Seven *S*. *aureus* patients, chosen from a larger cohort (n = 95) for analysis of serum antibody responses, based on evidence of induction of increased serum IgG responses to *S*. *aureus* antigens during convalescence^33^ (Supplementary Fig. S1). Four of the patients had SSTI, one had PJI, and two had PHO. Three serum samples from each of the seven *S*. *aureus* infected patients (obtained at initial presentation, short-term follow-up, and long-term follow-up) were used in the analysis.

### Bead-based multiplex immunoassays

To evaluate antigen-specific IgG-antibody reactivity, immunoassays were performed with cocktails of beads, each identified via internal fluorophores (MagPlex microspheres, Luminex). Each bead was coupled to an individual recombinant *S*. *aureus* protein, expressed in *S*. *aureus*, or control protein/ analyte (Supplementary Table S1), adapting previously described methods^54^. Assays were performed on serum sample at 1:100, 1:1000, 1:10,000 and 1:100,000 dilutions, with IgG binding detected with a PE-labeled goat anti-Human IgG Fc secondary antibody (eBioscienceTM, CAT:12-4998-82), using the MagPix xMap (Luminex) platform. Custom software was used to generate binding curves for each antigen, from which comparisons to the acute visit baseline sample were presented as fold-change in IgG titer.

### Solid-phase recombinant protein printed microarrays

Following their previously developed standard protocol (Antigen Discovery Inc.). 2,652 open reading frames (ORFs) from the USA300 genotype MRSA strain, FPR3757, were expressed *in vitro*, and printed onto nitrocellulose coated slides (for strain details, see www.atcc.org/Products/All/BAA-1556). 34 ORFs were split into a varying number of segments based on the length of the ORF. These are annotated SAUSA300-0000-s1 as an example for segment one of an ORF. Individual replicate slides were then incubated with blocking buffer. The patient samples were each diluted 1:100 in blocking buffer and incubated with a replicate slide, which were washed, and secondary PE-conjugated goat anti-human IgG (Fc gamma-specific) was added. After washing, slides were then scanned using Genepix 4300 scanner (Molecular Devices), with data analyzed using Genepix software with a validated protocol. IgG-binding data were normalized against control negative regions on the slide and log2 transformed, with the normalized value of 0 indicating no difference compared to background, and 1.0 indicating twice the signal compared to background.

### Data analysis

Normalized data were used to rank immune reactivity for each antigen, with further analysis using MATLAB® R2016b (MathWorks) and GraphPad Prism7® (Instat). Signal-peptide containing ORFs were identified with the CBS Prediction Server SignalP 4.1 (www.cbs.dtu.dk/services/SignalP/), by assignment of a Signal Peptide Prediction value (D), and a cutoff value of D = 0.5 or greater was used to identify the ORFs with signal sequences as previously described^55,56^. To identify transmembrane ORFs, we used the CBS Prediction Server TMHMM Server v 2.0 (www.cbs.dtu.dk/services/TMHMM/) to predict the number of transmembrane helices as previously described^57,58^. Significance between signal peptide containing proteins within the top 100 reactive with serum IgG (Fig. 1C), as well as for transmembrane containing proteins (Supplementary Fig. S5), was determined using a two-tailed Mann-Whitney test. Venn Diagram was made using BioVenn^59^.

Gene content was assayed by translated BLAST search of the draft genome contigs for each isolate, using as queries, protein sequences from the annotated genome of strain USA300 FPR-3757. The draft genome contigs from each isolate were built into individual BLAST databases then searched with each of the query proteins. The percent amino acid identity of the best match for each protein to each genome is reported. Proteins that match the draft genome with less than 50 percent amino acid identity are clearly absent (the best match is to a distantly related protein). Matches with greater than 90 percent identity are clearly present as closely related isoforms. Matches in the 50–90 percent range represent either distant gene isoforms or closely related members of a multi-gene family.

The phylogenetic tree was calculated using the shared kmer distance method as implemented in the AAF software v 20171001 with kmer size of 25. Assembled draft genome contigs were used as genome data for each isolate and the GenBank draft genome assembly for strain USA300 FPR-3757 (GCA-000013465) was used as a reference^60^. Phylogenetic tree drawings were made using FigTree v 1.4.3.

## Electronic supplementary material

Supplementary Information

## Data Availability

The datasets generated and analyzed in the current report are available from the corresponding author on reasonable request.
