# Supplementary material for: Hierarchy of human IgG recognition within the Staphylococcus aureus immunome
Source: Sci Rep. 2018 Sep 5;8:13296. doi: 10.1038/s41598-018-31424-3 (PMC6125462; doi:10.1038/s41598-018-31424-3)
Supplement: Supplementary file 1 — Supplementary Information [file 41598_2018_31424_MOESM1_ESM.pdf]

Supplementary Information:

Hierarchy of human IgG recognition within the *Staphylococcus aureus* immunome

Emily E. Radke,[1] Stuart M. Brown,[1] Adam J. Pelzek,[1] Yi Fulmer,[1] David N. Hernandez,[1] Victor J. Torres,[2] Isaac P. Thomsen,[3] William K. Chiang,[1] Andy O. Miller,[4] Bo Shopsin,[1] Gregg J. Silverman[1]*

1 New York University School of Medicine, Department of Medicine, New York, 10016, USA.

2 New York University School of Medicine, Department of Microbiology, New York, 10016, USA.

3 Vanderbilt University Medical Center, Department of Pediatrics, Division of Infectious Diseases, Nashville, 27232, USA.

4 Hospital for Special Surgery, New York, 10021, USA.

* Address correspondence to Gregg.Silverman@nyumc.org

Supplementary Figures:

Supplementary Figure S1. IgG fold-change between acute and convalescent visits in *S. aureus* by bead-based multiplex assay. A custom bead-based multiplex assay was used to analyze serially-diluted patient serum samples (acute, short-term follow-up, long-term follow-up), and fold-changes in IgG titer were determined by custom software analysis.

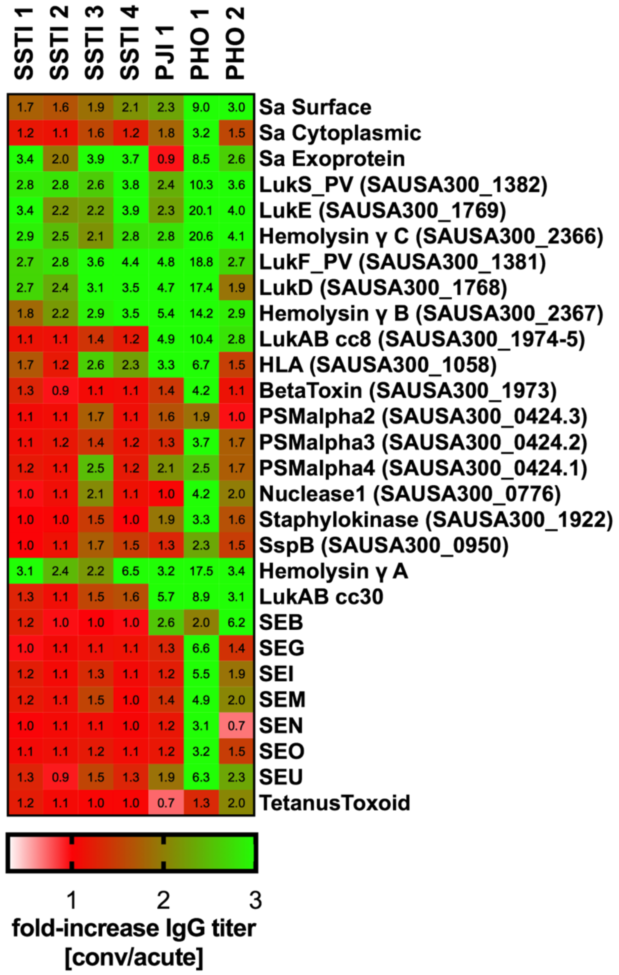

Supplementary Figure S2. Global serologic antibody responses of *S. aureus* infected patients against MRSA ORFs. Recombinant proteins from all ORFs (2652) from *S. aureus* strain USA300-FPR3757 were probed for binding of serum IgG from seven infected patients obtained at three different time points. Values were normalized for fluorescence intensity of control spots on each chip. Values above 1.0 were considered positively reactive as they are at least two-fold above background intensity for IgG-reactivity.

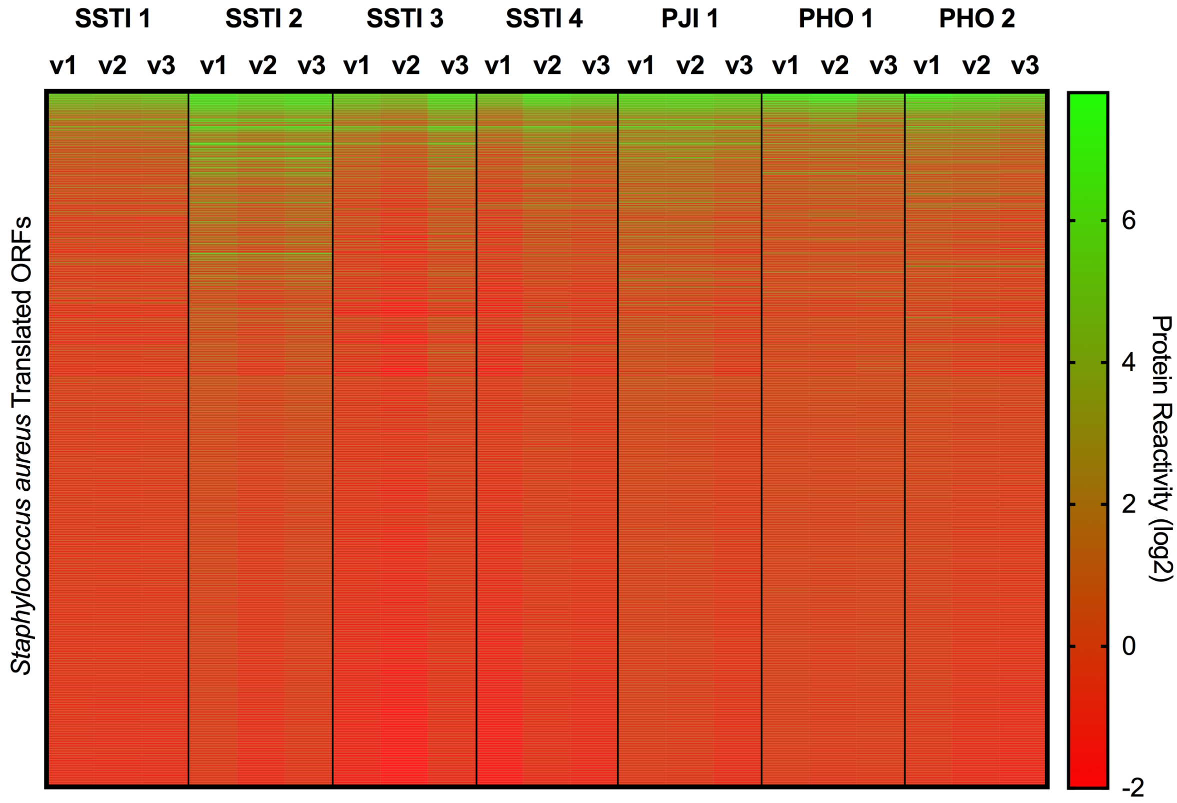

Supplementary Figure S3. Phylogenetic relationship between *S. aureus* infecting isolates and reference genome. The relationship between the seven patients infecting *S. aureus* isolates and the reference genome used in the protein array (USA300 FPR-3757). The assembled draft genome contigs were used as genome data for each isolate and the GenBank draft genome assembly for strain USA300 FPR-3757 (GCA_000013465) was used as a reference.

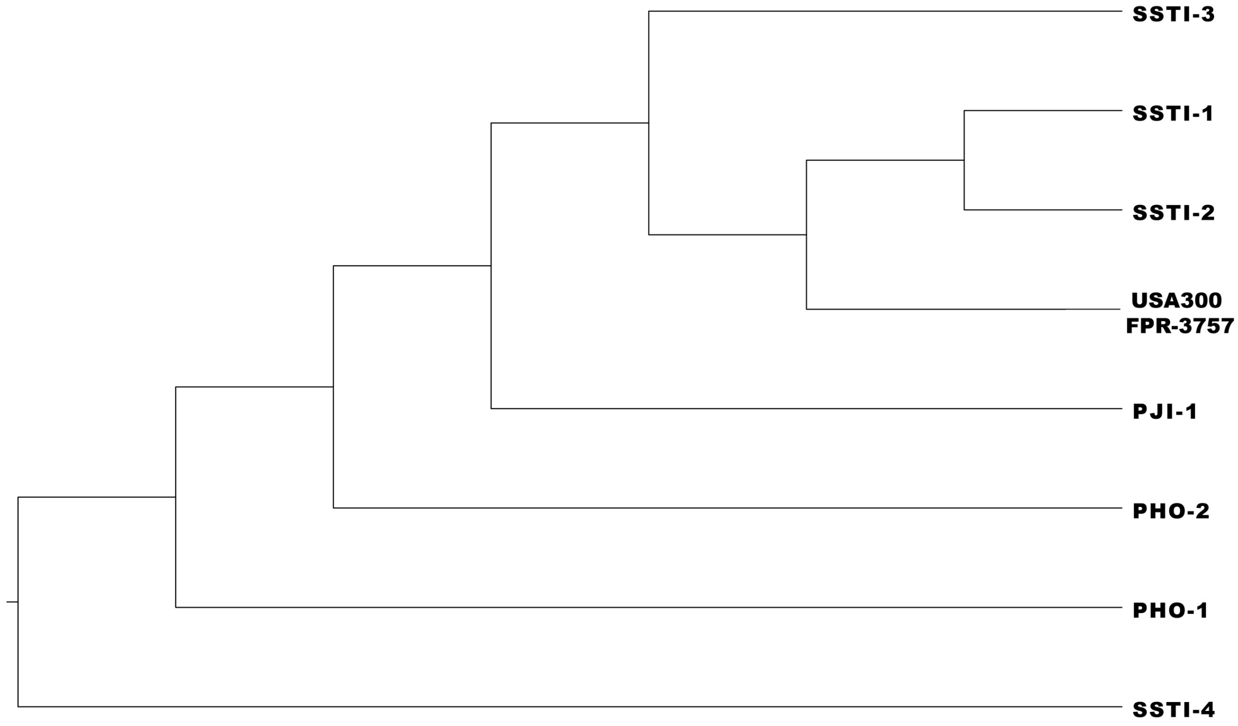

Supplementary Figure S4. Comparison of genomic content between reference and infecting *S. aureus* strains. Percentage amino acid identity deduced from genomic sequences was determined using a customized translated (tBLASTn) script for the ORFs for the top 50 IgG-reactive antigens from the protein array studies, with comparisons of the reference strain used in the array (USA300 FPR3757) with the sequence determined for the infecting strain for each patient. Amino acid sequence homology of 90% and above was generally considered a match for the representation of the gene of interest in infecting strain. Any percent identity below 90% could potentially be either a highly variant form of that protein or could be a match to a different protein with a related sequence. Proteins with percent identity below 50% are considered to be not present in that isolate.

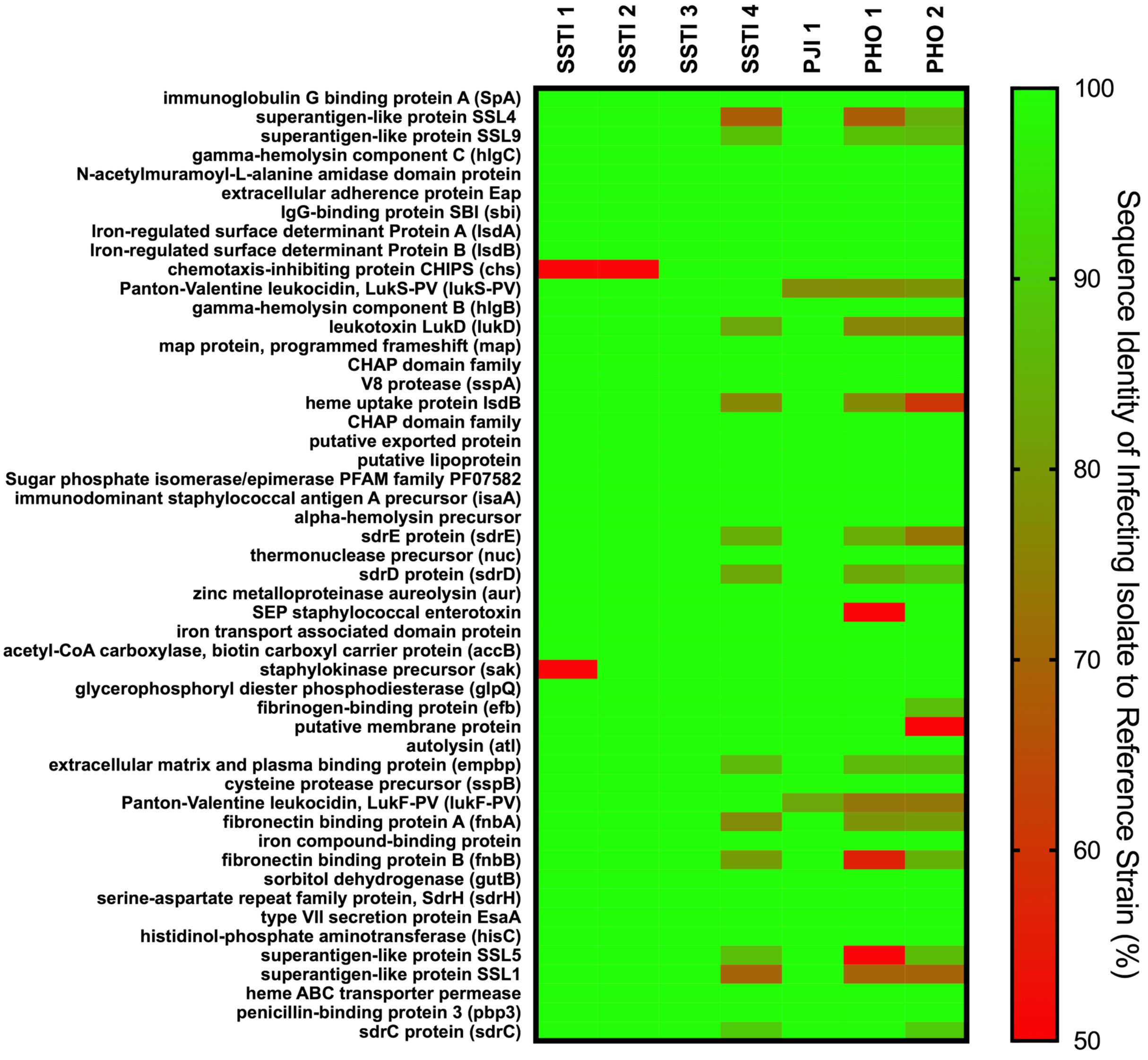

Supplementary Figure S5: Transmembrane domain-containing proteins in top and bottom 100 proteins. ORF sequences from the top and bottom 100 proteins identified by immunoarray were analyzed with the TMHMM Server and the representation of transmembrane domains thereby predicted. There was no significant difference in transmembrane containing proteins between the top and bottom 100.

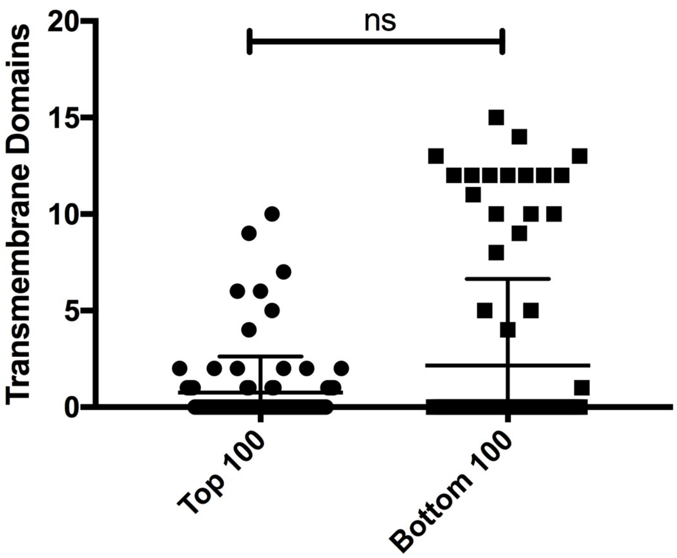

Supplementary Figure S6. IgG reactivity for *S. aureus* antigens by protein array correlates with the level of increased gene expression at the site of disease. We performed a Spearman correlation in GraphPad PRISM between IgG reactivity determined by protein array for each antigen with and fold-change in RNA expression for each ORF from abscess infection, as reported by Date et al. [32]. The vertical dotted line is the cut-off for antigens of two-fold above background for IgG reactivity. Similarly, the horizontal dotted line represents the cutoff for gene expression of two-fold above background expression after in vitro culture. The number of antigens in each group are shown by the boxed number in each quadrant. The top 10 reactive proteins from the protein array are highlighted in red and labeled.

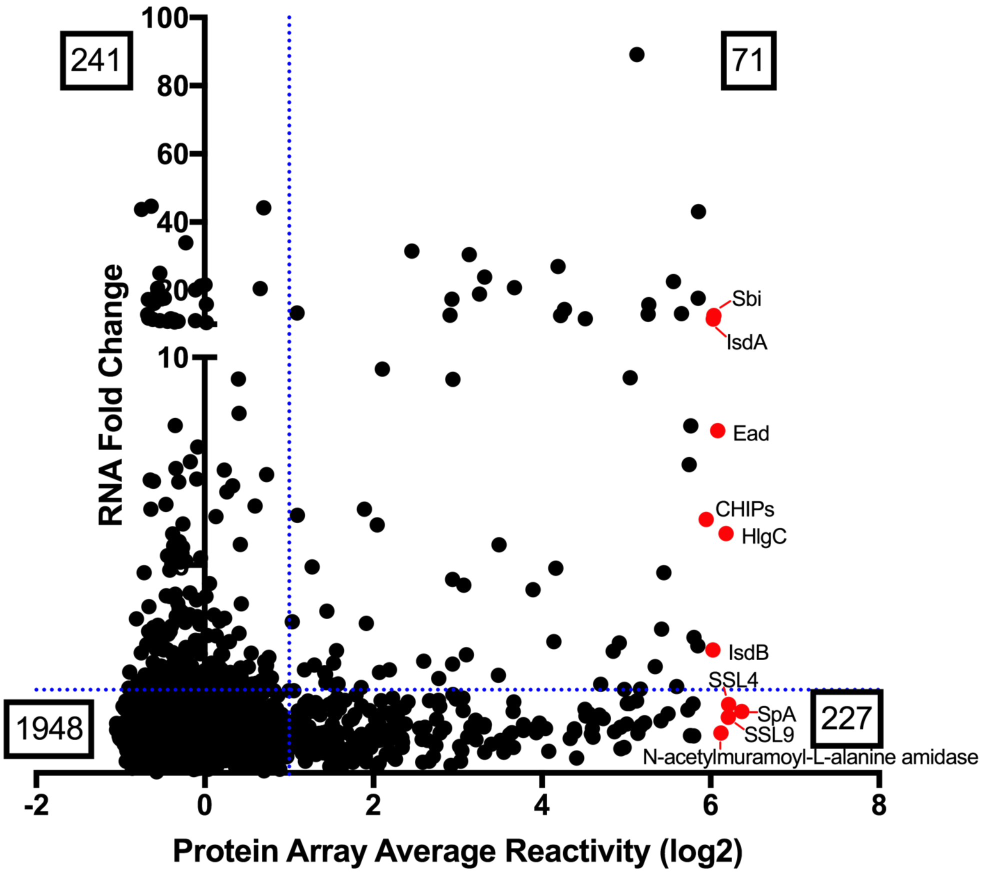

Supplementary Tables:

Supplementary Table S1. Antigens included in the multiplex bead-based assay. Ligands are assigned to categories; *S. aureus* antigens, or positive or negative controls. The full name and abbreviation of each antigen is indicated, as well as the source each antigen. The rank of IgG-reactivity for tested antigen in the chip-based protein array is also shown. N/A: Protein is not present in the array.

	Antigen	Abbreviation	Rank in Protein Array	Protein Array ID
**Positive Controls**	Tetanus Toxoid	Tetanus Tox	N/A	N/A
	Phosphorylcholine-16-BSA	PC16-BSA	N/A	N/A
	Phosphorylcholine-12-BSA	PC12-BSA	N/A	N/A
	Phosphorylcholine-4-BSA	PC4-BSA	N/A	N/A
**Ig-Binding**	anti-human IgG Fc-gamma Specific	anti-hIgG	N/A	N/A
	SpA domain D- WT (5x)	SpAD-WT5x	1	SAUSA300_0113
	SpA domain D- FcNull (5x)	SpAD-FcNull5x	N/A	N/A
**Negative Controls**	Bovine Serum Albumin	BSA	N/A	N/A
	Human Serum Albumin	HSA	N/A	N/A
	Azobenzenearsonate	ABA	N/A	N/A
***S. aureus* protein fractions**	surface extract	surface extract	N/A	N/A
	cytoplasmic extract	cytoplasmic extract	N/A	N/A
	exoproteins	exoproteins	N/A	N/A
***S. aureus* Exotoxins**	Leukocidin S	LukS-PV	11	SAUSA300_1382
	Leukocidin F	LukF-PV	38	SAUSA300_1381
	Leukocidin E	LukE	284	SAUSA300_1769
	Leukocidin D	LukD	13	SAUSA300_1768
	Leukocidin AB cc8	LukABcc8	LukAcc8 - 65	SAUSA300_1975
			LukBcc8 - 163	SAUSA300_1974
	Leukocidin AB cc30	LukABcc30	N/A	N/A
	Hemolysin-gamma A	HlgA	N/A	N/A
	Hemolysin-gamma B	HlgB	12	SAUSA300_2367
	Hemolysin-gamma C	HlgC	4	SAUSA300_2366
	Alpha Hemolysin	HLA	23	SAUSA300_1058
	β-Toxin	β-Toxin	176	SAUSA300_1973
**Superantigens or Superantigen-like proteins**	Staphylococcal Entertoxin B	SEB (*S. aureus)*	N/A	N/A
	Staphylococcal Entertoxin B (E. coli)	SEB (*E. coli)*	N/A	N/A
	Staphylococcal Entertoxin G	SEG	N/A	N/A
	Staphylococcal Entertoxin I	SEI	N/A	N/A
	Staphylococcal Entertoxin M	SEM	N/A	N/A
	Staphylococcal Entertoxin N	SEN	N/A	N/A
	Staphylococcal Entertoxin O	SEO	N/A	N/A
	Staphylococcal Entertoxin U	SEU	N/A	N/A
**Phenol Soluble Modulins**	Phenol Soluble Modulin 1	PSM1	740	SAUSA300_0424.4
	Phenol Soluble Modulin 2	PSM2	874	SAUSA300_0424.3
	Phenol Soluble Modulin 3	PSM3	256	SAUSA300_0424.2
	Phenol Soluble Modulin 4	PSM4	679	SAUSA300_0424.1
**Other *S. aureus* proteins**	Nuclease 1	Nuc1	25	SAUSA300_0776
	Staphylokinase	Sak	31	SAUSA300_1922
	Cysteine Protease SspB	SspB	37	SAUSA300_0950
**Other microbial antigens**	SP superantigen	SP superantigen	N/A	N/A
	Pneumococcal polysaccharide 12	Pneumo ps12	N/A	N/A
	Pneumococcal polysaccharide 23	Pneumo ps23	N/A	N/A
	Poly N-acetyl Glucosamine	PNAG	N/A	N/A
	Pneumococcal Cell Wall Polysaccharide	Pneumo CWPS	N/A	N/A
	S. pneumoniae pneumolysin	PLY	N/A	N/A
	S. pyogenes ArcA	ArcA	N/A	N/A

Supplementary Table S2. Antigens without significant IgG reactivity by protein array. Antigens that fell below the cutoff of two-fold above background are listed with their ranking number, locus tag and protein name/description.

Locus Tag	Protein Name/Description	Rank
SAUSA300_1965	conserved hypothetical phage protein	1087
SAUSA300_1694	tRNA (guanine-N(7)-)-methyltransferase (trmB) [2.1.1.33]	1088
SAUSA300_1333-s1	conserved hypothetical protein	1089
SAUSA300_0781	conserved hypothetical protein	1090
SAUSA300_1979	cation transport family protein	1091
SAUSA300_0017	adenylosuccinate synthetase (purA) [6.3.4.4]	1092
SAUSA300_0631	putative nucleoside transporter	1093
SAUSA300_0508	conserved hypothetical protein	1094
SAUSA300_1750-s1	conserved hypothetical protein	1095
SAUSA300_1802	conserved hypothetical protein	1096
SAUSA300_0260	6-phospho-beta-glucosidase (bglA) [3.2.1.86]	1097
SAUSA300_0608	conserved hypothetical protein	1098
SAUSA300_2063	ATP synthase F0, C subunit (atpE) [3.6.3.14]	1099
SAUSA300_1848	conserved hypothetical protein	1100
SAUSA300_0805	pathogenicity island protein	1101
SAUSA300_1899	conserved hypothetical protein	1102
SAUSA300_1295	cold shock protein, CSD family	1103
SAUSA300_1609	type III leader peptidase family protein	1104
SAUSA300_1710	putative lysophospholipase	1105
SAUSA300_1041	conserved hypothetical protein	1106
SAUSA300_2134	iron compound ABC transporter, permease protein	1107
SAUSA300_1163	ribosome-binding factor A (rbfA)	1108
SAUSA300_2142	alkaline shock protein 23 (asp23)	1109
SAUSA300_2047	thiamine-phosphate pyrophosphorylase (thiE) [2.5.1.3]	1110
SAUSA300_0347	Sec-independent protein translocase TatC (tatC)	1111
SAUSA300_2095	conserved hypothetical protein	1112
SAUSA300_2156	lactose phosphotransferase system repressor	1113
SAUSA300_0932	putative membrane protein	1114
SAUSA300_0812	conserved hypothetical protein	1115
SAUSA300_1400	phiSLT ORF92-like protein, uncharacterized phage protein (possible DNA packaging)	1116
SAUSA300_1662	aminotransferase, class V	1117
SAUSA300_2108	Mannitol-1-phosphate 5-dehydrogenase (mtlD) [1.1.1.17]	1118
pUSA01_0005	putative membrane protein	1119
SAUSA300_0811	conserved hypothetical protein	1120
SAUSA300_1405	phiSLT ORF 101-like protein, terminase, small subunit	1121
SAUSA300_0621	iron-dependent repressor	1122
SAUSA300_0361	ParB-like partition protein	1123
SAUSA300_0496	lysyl-tRNA synthetase (lysS) [6.1.1.6]	1124
SAUSA300_0850	Na(+)/H(+) antiporter subunit F (mnhF) [2.3.1.-]	1125
SAUSA300_1673	1-acyl-sn-glycerol-3-phosphate acyltransferases [2.3.1.51]	1126
SAUSA300_1275	peptide ABC transporter, permease protein	1127
SAUSA300_2591	conserved hypothetical protein	1128
SAUSA300_0762	preprotein translocase, SecG subunit (secG)	1129
SAUSA300_0657	conserved hypothetical protein	1130
SAUSA300_1144	glucose inhibited division protein (gid)	1131
SAUSA300_1203	conserved hypothetical protein	1132
SAUSA300_0808	conserved hypothetical protein	1133
SAUSA300_0421	conserved hypothetical protein	1134
SAUSA300_0632	conserved hypothetical protein	1135
SAUSA300_1541	co-chaperone GrpE (grpE)	1136
SAUSA300_1362	DNA-binding protein HU (hup)	1137
SAUSA300_2349	formate/nitrite transporter family protein	1138
SAUSA300_1248	conserved hypothetical protein	1139

SAUSA300_1210	conserved hypothetical protein	1140
SAUSA300_0345	Tat-translocated enzyme	1141
SAUSA300_0068	cadmium-exporting ATPase, truncation	1142
SAUSA300_0241	PTS system, sorbitol-specific IIC component (gatC)	1143
pUSA03_0004	conserved hypothetical protein	1144
SAUSA300_2256	putative N-acetylmuramoyl-L-alanine amidase	1145
SAUSA300_1687-s1	FtsK/SpoIIIE family protein	1146
SAUSA300_1196	RNA chaperone, host factor-1 protein (hfq)	1147
SAUSA300_0529	conserved hypothetical protein	1148
SAUSA300_0835	D-alanine-activating enzyme/D-alanine-D-alanyl, dltA protein (dltA)	1149
SAUSA300_1167	polyribopolyribonucleotide nucleotidyltransferase (pnpA) [2.7.7.8]	1150
SAUSA300_0519	conserved hypothetical protein	1151
SAUSA300_0300	conserved hypothetical protein	1152
SAUSA300_1583	conserved hypothetical protein	1153
SAUSA300_1445	segregation and condensation protein A (scpA)	1154
SAUSA300_1638	sensory box histidine kinase PhoR (phoR) [2.7.3.-]	1155
SAUSA300_1966	phi77 ORF014-like protein, phage anti-repressor protein	1156
SAUSA300_2008	acetolactate synthase, small subunit (ilvN) [4.1.3.18]	1157
SAUSA300_1432	phiSLT ORF78-like protein	1158
pUSA03_0018	transfer complex protein TraI (DNA topoisomerase III) (traI) [5.99.1.2]	1159
SAUSA300_1416	phiSLT ORF 81b-like protein	1160
SAUSA300_1945	phi77 ORF071-like protein	1161
SAUSA300_2348	conserved hypothetical protein	1162
SAUSA300_2519	putative cobalamin synthesis protein	1163
SAUSA300_2642	conserved hypothetical protein	1164
SAUSA300_0533	translation elongation factor Tu (tuf)	1165
SAUSA300_0849	Na(+)/H(+) antiporter subunit G (mnhG)	1166
SAUSA300_0080	conserved hypothetical protein	1167
SAUSA300_1997	conserved hypothetical protein	1168
SAUSA300_2191	30S ribosomal protein S14 (rpsN)	1169
SAUSA300_1527	GTP-binding protein Era (era)	1170
SAUSA300_2132	conserved hypothetical protein	1171
SAUSA300_0013	putative membrane protein	1172
SAUSA300_0325	glycine cleavage H-protein	1173
SAUSA300_0839	conserved hypothetical protein	1174
SAUSA300_2308	response regulator protein	1175
pUSA03_0026	conserved hypothetical protein	1176
pUSA03_0017	transfer complex protein TraH (traH)	1177
SAUSA300_0612	putative Na+/H+ antiporter, MnhC component	1178
SAUSA300_0882	conserved hypothetical protein	1179
SAUSA300_1259	ImpB/MucB/SamB family protein	1180
SAUSA300_0554	glucosamine-6-phosphate isomerase [3.5.99.6]	1181
SAUSA300_0792	conserved hypothetical protein	1182
SAUSA300_0675	conserved hypothetical protein	1183
SAUSA300_2205	30S ribosomal protein S10 (rpsJ)	1184
SAUSA300_1325	conserved hypothetical protein	1185
SAUSA300_1394	conserved hypothetical phage protein	1186
SAUSA300_0077	ABC transporter, ATP-binding protein	1187
SAUSA300_2102	haloacid dehalogenase-like hydrolase	1188
SAUSA300_0806	conserved hypothetical protein	1189
SAUSA300_0699	chorismate binding enzyme domain protein	1190
SAUSA300_2046	membrane protein oxaA precursor (oxaA)	1191
SAUSA300_0592	conserved hypothetical protein	1192
SAUSA300_1915	conserved hypothetical protein	1193
SAUSA300_1202	conserved hypothetical protein	1194
SAUSA300_0777	cold shock protein	1195
SAUSA300_1663	conserved hypothetical protein	1196

SAUSA300_2217	putative drug transporter	1197
SAUSA300_0915	conserved hypothetical protein	1198
SAUSA300_2580	isochorismatase family protein	1199
SAUSA300_1448	transcriptional regulator, Fur family	1200
SAUSA300_0624	teichoic acid translocation ATP-binding protein (tagH) [3.6.3.40]	1201
SAUSA300_0316	ROK family protein	1202
SAUSA300_0098	conserved hypothetical protein	1203
SAUSA300_1360	menaquinone biosynthesis methyltransferase ubiE (ubiE) [2.1.1.-]	1204
SAUSA300_1004	conserved hypothetical protein	1205
SAUSA300_1534	conserved hypothetical protein	1206
SAUSA300_2267	hydrolase, haloacid dehalogenase-like family	1207
SAUSA300_0715	nrdI protein (nrdI)	1208
SAUSA300_1930-s2	phi77 ORF001-like protein, phage tail tape measure protein	1209
SAUSA300_1749	conserved hypothetical protein	1210
SAUSA300_0104	transcriptional regulator, AraC family	1211
SAUSA300_1157-s2	DNA polymerase III, alpha subunit (polC) [2.7.7.7]	1212
SAUSA300_2466	putative membrane protein	1213
SAUSA300_0967	phosphoribosylaminoimidazole carboxylase, ATPase subunit (purK) [4.1.1.21]	1214
SAUSA300_1107	conserved hypothetical protein	1215
SAUSA300_2050	TENA/THI-4 family protein	1216
SAUSA300_0655	conserved hypothetical protein	1217
SAUSA300_1623	conserved hypothetical protein	1218
SAUSA300_0183	conserved hypothetical protein	1219
SAUSA300_2390	glycine betaine/carnitine/choline transport system permease (opuCd) [3.6.3.32]	1220
SAUSA300_0053	Spermidine N(1)-acetyltransferase (speG) [2.3.1.57]	1221
SAUSA300_2157	NAD-dependent deacetylase	1222
SAUSA300_0942	conserved hypothetical protein	1223
SAUSA300_0515	cysteinyl-tRNA synthetase (cysS) [6.1.1.16]	1224
pUSA01_0002	hypothetical protein	1225
SAUSA300_0816	CsbD-like superfamily	1226
SAUSA300_0581	conserved hypothetical protein	1227
SAUSA300_1461	conserved hypothetical protein	1228
SAUSA300_0896	oligopeptide ABC transporter, permease protein (oppC)	1229
SAUSA300_2098	transcriptional repressor, ArsR family (arsR)	1230
SAUSA300_0048	hypothetical protein	1231
SAUSA300_1343	endonuclease III (nth) [4.2.99.18]	1232
SAUSA300_1508	conserved hypothetical protein	1233
SAUSA300_2034	K+-transporting ATPase, A subunit (kdpA) [3.6.3.12]	1234
SAUSA300_1492	putative lipoprotein	1235
SAUSA300_0050	conserved hypothetical protein	1236
SAUSA300_1255	oxacillin resistance-related FmtC protein (fmtC)	1237
SAUSA300_2250	Na+/H+ antiporter NhaC (nhaC)	1238
SAUSA300_0960	quinol oxidase, subunit IV (qoxD) [1.9.3.-]	1239
SAUSA300_0015	50S ribosomal protein L9 (rplI)	1240
SAUSA300_2512	glyoxalase family protein	1241
SAUSA300_0120	siderophore biosynthesis protein, IucC family (sbnC)	1242
SAUSA300_0460	conserved hypothetical protein	1243
SAUSA300_2434	transporter protein	1244
SAUSA300_1209	conserved hypothetical protein	1245
SAUSA300_1489	conserved hypothetical protein	1246
SAUSA300_1205	conserved hypothetical protein	1247
SAUSA300_0906	conserved hypothetical protein	1248
SAUSA300_2527	conserved hypothetical protein	1249
SAUSA300_2074	50S ribosomal protein L31 type B (rpmE)	1250
SAUSA300_2499	squalene desaturase (crtM) [2.5.1.-]	1251

SAUSA300_1502	putative competence protein ComGC	1252
SAUSA300_0662	acetyltransferase, GNAT family	1253
SAUSA300_2625	transcriptional regulator, PadR family	1254
SAUSA300_1937	phi77 ORF045-like protein	1255
SAUSA300_2353	conserved hypothetical protein	1256
SAUSA300_2127	conserved hypothetical protein	1257
SAUSA300_1742	conserved hypothetical protein	1258
SAUSA300_2067	serine hydroxymethyltransferase (glyA) [2.1.2.1]	1259
SAUSA300_1314	conserved hypothetical protein	1260
SAUSA300_0937	conserved hypothetical protein	1261
SAUSA300_1750-s2	conserved hypothetical protein	1262
SAUSA300_2062	ATP synthase F0, B subunit (atpF) [3.6.3.14]	1263
SAUSA300_1131	30S ribosomal protein S16 (rpsP)	1264
SAUSA300_2555	glutathione peroxidase [1.11.1.9]	1265
SAUSA300_2433	phosphoglucomutase/phosphomannomutase family protein	1266
SAUSA300_1086	putative cell-division initiation protein	1267
SAUSA300_0595	conserved hypothetical protein	1268
SAUSA300_2056	conserved hypothetical protein	1269
SAUSA300_0826	conserved hypothetical protein	1270
SAUSA300_1983	10 kDa chaperonin (groES)	1271
SAUSA300_1969	phi77 ORF011-like protein, phage transcriptional repressor	1272
SAUSA300_2080	conserved hypothetical protein	1273
SAUSA300_0471	veg protein	1274
SAUSA300_1918	truncated beta-hemolysin	1275
SAUSA300_1079	cell division protein ftsA (ftsA)	1276
SAUSA300_0664	conserved hypothetical protein	1277
SAUSA300_1598	holliday junction DNA helicase RuvA (ruvA)	1278
SAUSA300_1096-s1	carbamoyl-phosphate synthase, large subunit (carB) [6.3.5.5]	1279
SAUSA300_1603	50S ribosomal protein L21 (rplU)	1280
SAUSA300_0163	capsular polysaccharide biosynthesis protein Cap5L (cap5L) [2.4.1.-]	1281
SAUSA300_1125	acyl carrier protein (acpP)	1282
SAUSA300_1221	conserved hypothetical protein	1283
SAUSA300_1230	conserved hypothetical protein	1284
SAUSA300_2283	ribose 5-phosphate isomerase A (rpiA) [5.3.1.6]	1285
SAUSA300_0901	putative competence protein	1286
SAUSA300_2396	para-nitrobenzyl esterase (pnbA) [3.1.1.-]	1287
SAUSA300_2280	metallothiol transferase fosB (fosB) [2.5.1.18]	1288
SAUSA300_0949	cysteine protease (sspC)	1289
SAUSA300_1319	dihydrofolate reductase (folA) [1.5.1.3]	1290
SAUSA300_0920	conserved hypothetical protein	1291
SAUSA300_0538	NAD dependent epimerase/dehydratase family [5.1.3.2]	1292
SAUSA300_1927	phi77 ORF109-like protein	1293
SAUSA300_0423	conserved hypothetical protein	1294
SAUSA300_1692	conserved hypothetical protein	1295
SAUSA300_2384	putative Na+/H+ antiporter	1296
SAUSA300_0782	conserved hypothetical protein	1297
SAUSA300_0254	sensor histidine kinase	1298
SAUSA300_0211	maltose ABC transporter, permease protein	1299
SAUSA300_0181-s1	non-ribosomal peptide synthetase	1300
SAUSA300_1021	hypothetical protein	1301
SAUSA300_2493	conserved hypothetical protein	1302
SAUSA300_2193	50S ribosomal protein L24 (rplX)	1303
SAUSA300_0779	conserved hypothetical protein	1304
SAUSA300_1935	phi77 ORF029-like protein	1305
SAUSA300_2215	conserved hypothetical protein	1306
SAUSA300_0055	alcohol dehydrogenase, zinc-containing	1307

SAUSA300_0439	conserved hypothetical protein	1308
SAUSA300_1253	transcription antiterminator (glcT)	1309
SAUSA300_1160	conserved hypothetical protein	1310
SAUSA300_1990	accessory gene regulator protein D (agrD)	1311
SAUSA300_1469	arginine repressor (argR)	1312
SAUSA300_2360	multidrug resistance protein	1313
SAUSA300_2286	conserved hypothetical protein	1314
SAUSA300_2143	conserved hypothetical protein	1315
SAUSA300_2294	conserved hypothetical protein	1316
SAUSA300_1040	conserved hypothetical protein	1317
SAUSA300_0719	iron compound ABC transporter, permease protein	1318
SAUSA300_1388	phiSLT ORF488-like protein	1319
SAUSA300_1607	conserved hypothetical protein	1320
SAUSA300_0106	putative drug transporter	1321
SAUSA300_0785	acetyltransferase, GNAT family [2.3.1.-]	1322
SAUSA300_2361	conserved hypothetical protein	1323
SAUSA300_0305	formate/nitrite transporter family protein	1324
SAUSA300_2638	conserved hypothetical protein	1325
SAUSA300_2408	oligopeptide ABC transporter, ATP-binding protein	1326
SAUSA300_2524	conserved hypothetical protein	1327
SAUSA300_2223	molybdopterin-guanine dinucleotide biosynthesis protein B (mobB)	1328
SAUSA300_0232	conserved hypothetical protein	1329
SAUSA300_0197	conserved hypothetical protein	1330
SAUSA300_0694	putative membrane protein	1331
SAUSA300_0908	NAD(+)/NADH kinase [2.7.1.-]	1332
SAUSA300_0029	conserved hypothetical protein	1333
SAUSA300_0189	isochorismatase (entB) [3.3.2.1]	1334
SAUSA300_2514	conserved hypothetical protein	1335
SAUSA300_1180	conserved hypothetical protein	1336
SAUSA300_2199	50S ribosomal protein L22 (rplV)	1337
SAUSA300_1647	acetyl-CoA carboxylase, carboxyl transferase, beta subunit (accD) [6.4.1.2]	1338
pUSA03_0011	transfer complex protein TraB (traB)	1339
SAUSA300_1926	phi77 ORF044-like protein	1340
SAUSA300_2481	conserved hypothetical protein	1341
SAUSA300_0790	putative arsenate reductase	1342
SAUSA300_1803	conserved hypothetical protein	1343
SAUSA300_2202	50S ribosomal protein L23 (rplW)	1344
SAUSA300_1932	conserved hypothetical phage protein	1345
SAUSA300_0239	PTS system, fructose-specific enzyme II, BC component [2.7.1.69]	1346
SAUSA300_2355	putative lipoprotein	1347
SAUSA300_0710	hypothetical protein	1348
SAUSA300_0512	PIN domain protein	1349
SAUSA300_0035	truncated hypothetical protein, similar to type I restriction-modification system endonuclease	1350
SAUSA300_0789	putative thioredoxin	1351
SAUSA300_1430	phiSLT ORF 87-like protein, putative DNA-binding protein	1352
SAUSA300_0059	conserved hypothetical protein	1353
SAUSA300_2196	50S ribosomal protein L29 (rpmC)	1354
SAUSA300_2335	conserved hypothetical protein	1355
SAUSA300_1256	peptide methionine sulfoxide reductase MsrA (msrA) [1.8.4.6]	1356
SAUSA300_1431	phiSLT ORF71-like protein	1357
SAUSA300_2508	conserved hypothetical protein	1358
SAUSA300_1240	conserved hypothetical protein	1359
SAUSA300_2144	conserved hypothetical protein	1360
SAUSA300_1024	phosphopantetheine adenylyltransferase (coaD) [2.7.7.3]	1361

SAUSA300_1166	30S ribosomal protein S15 (rpsO)	1362
SAUSA300_1258	4-oxalocrotonate tautomerase [5.3.2.-]	1363
SAUSA300_1873	Mur ligase family protein	1364
SAUSA300_0451	acetyltransferase, GNAT family [2.3.1.-]	1365
SAUSA300_0352	ABC transporter, ATP-binding protein	1366
SAUSA300_1395	phiSLT ORF116b-like protein	1367
SAUSA300_1008	conserved hypothetical protein	1368
SAUSA300_0615	putative Na+/H+ antiporter, MnhF component	1369
SAUSA300_0658	transcriptional regulator, LysR family	1370
SAUSA300_1874	ferritins family protein	1371
SAUSA300_0561	hypothetical protein	1372
SAUSA300_0744	prolipoprotein diacylglyceryl transferase (lgt) [2.4.99.-]	1373
SAUSA300_0374	putative membrane protein	1374
SAUSA300_1276	oligopeptide permease, channel-forming protein (opp-2B)	1375
SAUSA300_2093	conserved hypothetical protein	1376
SAUSA300_0545	Flavodoxin-like fold [1.5.1.29]	1377
SAUSA300_0837	D-alanine-activating enzyme/D-alanine-D-alanyl, dltC protein (dltC)	1378
SAUSA300_1352	putative membrane protein	1379
SAUSA300_1518	ATP-dependent RNA helicase, DEAD/DEAH box family	1380
SAUSA300_2053	conserved hypothetical protein	1381
SAUSA300_1265	indole-3-glycerol phosphate synthase (trpC) [4.1.1.48]	1382
SAUSA300_1297	conserved hypothetical protein	1383
SAUSA300_2425	conserved hypothetical protein	1384
SAUSA300_2158	conserved hypothetical protein	1385
SAUSA300_1342	conserved hypothetical protein	1386
SAUSA300_1298	putative XpaC protein	1387
SAUSA300_1434	phiSLT ORF104a-like protein, repressor	1388
SAUSA300_0494	2-amino-4-hydroxy-6-hydroxymethyldihydropteridine pyrophosphokinase (folK) [2.7.6.3]	1389
SAUSA300_2544	conserved hypothetical protein	1390
SAUSA300_1440	conserved hypothetical protein	1391
SAUSA300_2428	staphylococcus tandem lipoprotein	1392
SAUSA300_0933	conserved hypothetical protein	1393
SAUSA300_2619	conserved hypothetical protein	1394
SAUSA300_0619	ABC transporter, permease protein	1395
SAUSA300_2200	30S ribosomal protein S19 (rpsS)	1396
SAUSA300_0761	conserved hypothetical protein	1397
SAUSA300_1653	conserved hypothetical protein	1398
SAUSA300_1496	glycine dehydrogenase, subunit 2 [1.4.4.2]	1399
SAUSA300_0917	putative membrane protein	1400
SAUSA300_0058	conserved hypothetical protein	1401
SAUSA300_1208	conserved hypothetical protein	1402
SAUSA300_2647	ribonuclease P protein component (rnpA) [3.1.26.5]	1403
SAUSA300_2131	conserved hypothetical protein	1404
SAUSA300_0334	transcriptional regulator, MarR family	1405
SAUSA300_1746	conserved hypothetical protein	1406
SAUSA300_0823	hypothetical protein	1407
SAUSA300_2181	50S ribosomal protein L36 (rpmJ)	1408
SAUSA300_0607	conserved hypothetical protein	1409
SAUSA300_1717	arsenical resistance operon repressor (arsR)	1410
SAUSA300_0480	peptidyl-tRNA hydrolase (pth) [3.1.1.29]	1411
SAUSA300_1016	protoheme IX farnesyltransferase (cyoE) [2.5.1.-]	1412
SAUSA300_0940	conserved hypothetical protein	1413
SAUSA300_2359	amino acid ABC transporter, amino acid-binding protein	1414

SAUSA300_1499	shikimate kinase (aroK) [2.7.1.71]	1415
SAUSA300_0385	conserved hypothetical protein	1416
SAUSA300_1679	acetyl-coenzyme A synthetase (acsA) [6.2.1.1]	1417
SAUSA300_0488	hypoxanthine phosphoribosyltransferase (hpt) [2.4.2.8]	1418
SAUSA300_1377	conserved hypothetical protein	1419
SAUSA300_1356	3-dehydroquinate synthase (aroB) [4.2.3.4]	1420
SAUSA300_0057	conserved hypothetical protein	1421
SAUSA300_1709	transposase, degenerate	1422
SAUSA300_0767	conserved hypothetical protein	1423
SAUSA300_1375	conserved hypothetical protein	1424
SAUSA300_1924	holin	1425
SAUSA300_0193	conserved hypothetical protein	1426
SAUSA300_1117	50S ribosomal protein L28 (rpmB)	1427
SAUSA300_1573	conserved hypothetical protein TIGR00250	1428
SAUSA300_1949	dUTP diphosphatase (dut) [3.6.1.23]	1429
SAUSA300_0956	conserved hypothetical protein	1430
SAUSA300_1488	conserved hypothetical protein	1431
SAUSA300_2185	50S ribosomal protein L15 (rplO)	1432
SAUSA300_2263	putative transposase	1433
SAUSA300_2452	transcriptional regulator, MarR family	1434
SAUSA300_0376	conserved hypothetical protein	1435
SAUSA300_1686	UDP-N-acetylmuramate--alanine ligase (murC) [6.3.2.8]	1436
SAUSA300_0112	L-lactate permease (lctP)	1437
SAUSA300_2052	single-stranded DNA- binding protein family	1438
SAUSA300_2621	conserved hypothetical protein	1439
SAUSA300_2340	respiratory nitrate reductase, gamma subunit (narI) [1.7.99.4]	1440
SAUSA300_2609	imidazole glycerol phosphate dehydratase hisB (hisB) [4.2.1.19]	1441
SAUSA300_1274	peptide ABC transporter, ATP-binding protein	1442
SAUSA300_1505	conserved hypothetical protein	1443
SAUSA300_0713	GTP cyclohydrolase I (folE) [3.5.4.16]	1444
SAUSA300_1963	conserved hypothetical phage protein	1445
SAUSA300_0553	conserved hypothetical protein	1446
SAUSA300_0692	conserved hypothetical protein	1447
SAUSA300_1950	conserved hypothetical phage protein	1448
SAUSA300_0392	conserved hypothetical protein	1449
SAUSA300_0596	arginyl-tRNA synthetase (argS) [6.1.1.19]	1450
SAUSA300_0256	holin-like protein lrgA	1451
SAUSA300_2635	conserved hypothetical protein	1452
SAUSA300_0076	ABC transporter, ATP-binding protein	1453
SAUSA300_1429	phiSLT ORF53-like protein	1454
SAUSA300_1412	phiSLT ORF 50-like protein, similar to transcriptional activator rinB	1455
SAUSA300_1054	conserved hypothetical protein	1456
SAUSA300_1925	phiPVL ORF17-like protein	1457
SAUSA300_2495	copper chaperone copZ	1458
SAUSA300_0155	capsular polysaccharide biosynthesis protein Cap5D, authentic frameshift (cap5D)	1459
SAUSA300_1027	50S ribosomal protein L32 (rpmF)	1460
SAUSA300_1529	diacylglycerol kinase (dgkA) [2.7.1.107]	1461
SAUSA300_0090	conserved hypothetical protein	1462
SAUSA300_2464	hydrolase, haloacid dehalogenase-like family [1.6.99.3]	1463
SAUSA300_0597	putative endonuclease III	1464
SAUSA300_1643	transposase, authentic frameshift	1465
SAUSA300_1268	tryptophan synthase, alpha subunit (trpA) [4.2.1.20]	1466
SAUSA300_1355	3-phosphoshikimate 1-carboxyvinyltransferase (aroA) [2.5.1.19]	1467

SAUSA300_1635	formamidopyrimidine-DNA glycosylase (mutM) [3.2.2.23]	1468
SAUSA300_0749	conserved hypothetical protein	1469
SAUSA300_2488	ferrous iron transport protein A (feoA)	1470
SAUSA300_1484	conserved hypothetical protein	1471
SAUSA300_1351	conserved hypothetical protein	1472
SAUSA300_1099	conserved hypothetical protein	1473
SAUSA300_2639	cold shock protein	1474
SAUSA300_1084	conserved hypothetical protein	1475
SAUSA300_0458	Orn/Lys/Arg decarboxylase [4.1.1.18]	1476
SAUSA300_0931	conserved hypothetical protein	1477
SAUSA300_0323	conserved hypothetical protein	1478
SAUSA300_1601	50S ribosomal protein L27 (rpmA)	1479
SAUSA300_0660	conserved hypothetical protein	1480
SAUSA300_0493	dihydroneopterin aldolase (folB) [4.1.2.25]	1481
SAUSA300_0378	conserved hypothetical protein	1482
SAUSA300_2326	transcription regulatory protein	1483
SAUSA300_0073	peptide ABC transporter, peptide-binding protein	1484
SAUSA300_2339	conserved hypothetical protein	1485
SAUSA300_1422	phiSLT ORF65-like protein	1486
SAUSA300_0009	seryl-tRNA synthetase (serS) [6.1.1.11]	1487
SAUSA300_0656	conserved hypothetical protein	1488
SAUSA300_0315	N-acetylneuraminate lyase subunit (nanA) [4.1.3.3]	1489
SAUSA300_1748	transposase, authentic frameshift	1490
SAUSA300_2564	tributyrin esterase (estA)	1491
SAUSA300_1116	thiamine pyrophosphokinase [2.7.6.2]	1492
SAUSA300_2595	acetyltransferase, GNAT family	1493
SAUSA300_0544	hydrolase, haloacid dehalogenase-like family	1494
SAUSA300_0043	conserved hypothetical protein	1495
SAUSA300_1493	conserved hypothetical protein	1496
SAUSA300_1038	phenylalanyl-tRNA synthetase, beta subunit (pheT) [6.1.1.20]	1497
SAUSA300_1952	phiPV083 ORF027-like protein	1498
SAUSA300_2221	molybdopterin converting factor, subunit 1 (moaD)	1499
SAUSA300_1358	nucleoside diphosphate kinase superfamily (ndk) [2.7.4.6]	1500
SAUSA300_1339	conserved hypothetical protein	1501
SAUSA300_2337	transcriptional regulator, DegU family	1502
SAUSA300_0481	transcription-repair coupling factor (mfd)	1503
SAUSA300_2632	putative membrane protein	1504
SAUSA300_0728	conserved hypothetical protein	1505
SAUSA300_0842	conserved hypothetical protein	1506
SAUSA300_1506	conserved hypothetical protein	1507
SAUSA300_1406	phiSLT ORF 104b-like protein	1508
SAUSA300_2309	sensor histidine kinase	1509
SAUSA300_2066	uracil phosphoribosyltransferase (upp) [2.4.2.9]	1510
SAUSA300_1373	ferredoxin	1511
SAUSA300_2533	pantoate--beta-alanine ligase (panC) [6.3.2.1]	1512
SAUSA300_0130	NAD-dependent epimerase/dehydratase family protein	1513
SAUSA300_1877	conserved hypothetical protein	1514
SAUSA300_0874	conserved hypothetical protein	1515
SAUSA300_1849	A/G-specific adenine glycosylase (mutY) [3.2.2.-]	1516
SAUSA300_1277	conserved hypothetical protein	1517
SAUSA300_2048	hydroxyethylthiazole kinase (thiM) [2.7.1.50]	1518
SAUSA300_2345	nitrite reductase [NAD(P)H], small subunit (nirD) [1.7.1.4]	1519
SAUSA300_1931	phi77 ORF100-like protein	1520
SAUSA300_1161	ribosomal protein L7Ae	1521
SAUSA300_0044	metallo-beta-lactamase family protein	1522
SAUSA300_2040	putative membrane protein	1523

SAUSA300_0741	excinuclease ABC, B subunit (uvrB)	1524
SAUSA300_0263	ribose permease (rbsD)	1525
SAUSA300_2535	2-dehydropantoate 2-reductase (panE) [1.1.1.169]	1526
SAUSA300_0049	hypothetical protein	1527
SAUSA300_0331	conserved hypothetical protein	1528
SAUSA300_0321	conserved hypothetical protein	1529
SAUSA300_1853	conserved hypothetical protein	1530
SAUSA300_0884	conserved hypothetical protein	1531
SAUSA300_2331	transcriptional regulator, MarR family	1532
SAUSA300_0556	SIS domain protein	1533
SAUSA300_1294	conserved hypothetical protein	1534
SAUSA300_1206	conserved hypothetical protein	1535
SAUSA300_0827	putative membrane protein	1536
SAUSA300_1383	phiSLT ORF484-like protein, lysin [3.5.1.28]	1537
SAUSA300_0363	conserved hypothetical protein	1538
SAUSA300_0490	33 kDa chaperonin (Heat shock protein 33 homolog)	1539
SAUSA300_1296	conserved hypothetical protein	1540
SAUSA300_2204	50S ribosomal protein L3 (rplC)	1541
SAUSA300_1585	conserved hypothetical protein	1542
SAUSA300_2070	conserved hypothetical protein	1543
SAUSA300_0678	putative membrane protein	1544
SAUSA300_0465	conserved hypothetical protein	1545
SAUSA300_1271	hydrolase-related protein	1546
SAUSA300_0659	sugar efflux transporter	1547
SAUSA300_0353	conserved hypothetical protein	1548
SAUSA300_0520	preprotein translocase, SecE subunit	1549
SAUSA300_2295	conserved hypothetical protein	1550
SAUSA300_2534	3-methyl-2-oxobutanoate hydroxymethyltransferase (panB) [2.1.2.11]	1551
SAUSA300_2338	sensor histidine kinase	1552
SAUSA300_2522	conserved hypothetical protein	1553
SAUSA300_1223	conserved hypothetical protein	1554
SAUSA300_1703	rhodanese-like domain protein	1555
SAUSA300_0618	ABC transporter, substrate-binding protein	1556
SAUSA300_1921	truncated amidase	1557
SAUSA300_0700	conserved hypothetical protein	1558
SAUSA300_1958	Single-strand binding protein	1559
SAUSA300_2401	addiction module toxin, Txe/YoeB family	1560
SAUSA300_0977	cobalt transport family protein	1561
SAUSA300_2266	conserved hypothetical protein	1562
SAUSA300_1511	50S ribosomal protein L33 (rpmG)	1563
SAUSA300_1980	acetyltransferase, GNAT family	1564
SAUSA300_1409	conserved hypothetical phage protein	1565
SAUSA300_0181-s2	non-ribosomal peptide synthetase	1566
SAUSA300_1882	aspartyl/glutamyl-tRNA amidotransferase subunit C (gatC) [6.3.5.-]	1567
SAUSA300_2423	conserved hypothetical protein	1568
SAUSA300_1404	phiSLT ORF 563-like protein, terminase, large subunit	1569
SAUSA300_0173	conserved hypothetical protein	1570
SAUSA300_1380	conserved hypothetical protein	1571
SAUSA300_0878	transcriptional regulator, LysR family	1572
SAUSA300_0476	hypothetical protein	1573
SAUSA300_2094	conserved hypothetical protein	1574
SAUSA300_1867	conserved hypothetical protein	1575
SAUSA300_1279	phosphate transport system regulatory protein PhoU (phoU)	1576
SAUSA300_1626	50S ribosomal protein L35 (rpmI)	1577
SAUSA300_0063	cyclic nucleotide-binding domain protein	1578
SAUSA300_2450	DedA family protein	1579
SAUSA300_1318	DegV family protein	1580

SAUSA300_1419	phiSLT ORF80-like protein	1581
SAUSA300_1213	conserved hypothetical protein	1582
SAUSA300_2245	staphylococcal accessory regulator R	1583
SAUSA300_2222	molybdopterin converting factor, subunit 2 (moaE)	1584
SAUSA300_2218	staphylococcal accessory regulator	1585
SAUSA300_1088	glyoxalase family protein [4.4.1.5]	1586
SAUSA300_2091	purine nucleoside phosphorylase (deoD) [2.4.2.1]	1587
SAUSA300_1017	conserved hypothetical protein	1588
SAUSA300_2042	conserved hypothetical protein	1589
SAUSA300_1479	conserved hypothetical protein	1590
pUSA01_0003	putative membrane protein	1591
SAUSA300_1967	conserved hypothetical phage protein	1592
SAUSA300_0236	PTS system, IIBC components [2.7.1.69]	1593
SAUSA300_2029	conserved hypothetical protein	1594
SAUSA300_2480	transcriptional regulator, LysR family	1595
SAUSA300_1723	conserved hypothetical protein	1596
SAUSA300_0207	conserved hypothetical protein	1597
SAUSA300_0278	conserved hypothetical protein	1598
SAUSA300_2389	putative drug transporter	1599
SAUSA300_0576	putative Pyridine nucleotide-disulphide oxidoreductase	1600
SAUSA300_2617	putative cobalt ABC transporter, ATP-binding protein	1601
SAUSA300_1222	thermonuclease (nuc) [3.1.31.1]	1602
SAUSA300_2272	conserved hypothetical protein	1603
SAUSA300_2511	conserved hypothetical protein	1604
SAUSA300_0290	putative lipoprotein	1605
SAUSA300_1392	phiSLT ORF191-like protein	1606
SAUSA300_0484	conserved hypothetical protein	1607
SAUSA300_1857	conserved hypothetical protein	1608
SAUSA300_2648	50S ribosomal protein L34 (rpmH)	1609
SAUSA300_1035	conserved hypothetical protein	1610
SAUSA300_1100	conserved hypothetical protein	1611
SAUSA300_2212	conserved hypothetical protein	1612
SAUSA300_1738	putative lipoprotein	1613
SAUSA300_1126	ribonuclease III (rnc) [3.1.26.3]	1614
SAUSA300_0431	conserved hypothetical protein	1615
SAUSA300_0804	putative transcriptional regulator	1616
SAUSA300_1366	conserved hypothetical protein	1617
SAUSA300_1251	DNA topoisomerase IV, subunit A (parC) [5.99.1.-]	1618
SAUSA300_1614	glutamate-1-semialdehyde-2,1-aminomutase (hemL) [5.4.3.8]	1619
SAUSA300_2077	conserved hypothetical protein	1620
SAUSA300_1844	bacterioferritin comigratory protein [1.11.1.-]	1621
SAUSA300_1215	conserved hypothetical protein	1622
SAUSA300_0100	staphylococcus tandem lipoprotein	1623
SAUSA300_0614	putative Na+/H+ antiporter, MnhE component	1624
SAUSA300_0875	conserved hypothetical protein	1625
SAUSA300_0628	teichoic acid biosynthesis protein D [2.7.7.39]	1626
SAUSA300_0667	YaiI/YqxD family protein	1627
SAUSA300_1485	conserved hypothetical protein	1628
SAUSA300_1231	gamma-aminobutyrate permease	1629
SAUSA300_2352	addiction module toxin, Txe/YoeB family	1630
SAUSA300_0365	conserved hypothetical protein	1631
SAUSA300_0011	conserved hypothetical protein	1632
SAUSA300_1233	50S ribosomal protein L33 (rpmG)	1633
SAUSA300_0688	oxidoreductase, aldo/keto reductase family	1634
SAUSA300_2392	glycine betaine/carnitine/choline ABC transporter (opuCb) [3.6.3.32]	1635
SAUSA300_1384	phiSLT ORF100b-like protein, holin	1636
SAUSA300_1554	conserved hypothetical protein	1637

SAUSA300_1721	conserved hypothetical protein	1638
SAUSA300_1184	conserved hypothetical protein	1639
SAUSA300_0961	quinol oxidase, subunit III (qoxC) [1.9.3.-]	1640
SAUSA300_2180	30S ribosomal protein S13 (rpsM)	1641
SAUSA300_0010	putative membrane protein	1642
pUSA03_0035	conserved hypothetical protein	1643
SAUSA300_0066	arginine repressor (argR)	1644
SAUSA300_0428	conserved hypothetical protein	1645
SAUSA300_2133	transporter gate domain protein	1646
SAUSA300_0135	Superoxide dismutase (Mn/Fe family) [1.15.1.1]	1647
SAUSA300_1212	conserved hypothetical protein	1648
SAUSA300_2594	methionine-S-sulfoxide reductase (msrA) [1.8.4.6]	1649
SAUSA300_1953	phiPVL ORF051-like protein	1650
SAUSA300_2195	30S ribosomal protein S17 (rpsQ)	1651
SAUSA300_1909	conserved hypothetical protein	1652
SAUSA300_0585	conserved hypothetical protein	1653
SAUSA300_2257	conserved hypothetical protein	1654
SAUSA300_0840	conserved hypothetical protein	1655
SAUSA300_2571	arginine repressor (argR)	1656
SAUSA300_2145	glycine betaine transporter	1657
SAUSA300_0697	exsB protein	1658
SAUSA300_2615	conserved hypothetical protein	1659
SAUSA300_0831	conserved hypothetical protein	1660
SAUSA300_0158	capsular polysaccharide biosynthesis protein Cap5G (cap5G) [5.1.3.14]	1661
SAUSA300_0027	conserved hypothetical protein	1662
SAUSA300_2287	putative membrane protein	1663
SAUSA300_1726	crcB family protein	1664
SAUSA300_0342	conserved hypothetical protein	1665
SAUSA300_1470	geranyltranstransferase [2.5.1.10]	1666
SAUSA300_0067	universal stress protein family	1667
SAUSA300_0832	conserved hypothetical protein	1668
SAUSA300_2451	drug transporter	1669
SAUSA300_1324	putative membrane protein	1670
SAUSA300_0350	transcriptional regulator, Cro/CI family-related protein	1671
SAUSA300_1943	phi77 ORF040-like protein	1672
SAUSA300_1211	conserved hypothetical protein	1673
SAUSA300_0148	conserved hypothetical protein	1674
SAUSA300_0216	hexose phosphate transport protein (uhpT)	1675
SAUSA300_2152	tagatose 1,6-diphosphate aldolase (lacD) [4.1.2.40]	1676
SAUSA300_2274	putative membrane protein	1677
SAUSA300_0916	conserved hypothetical protein	1678
SAUSA300_0075	oligopeptide permease, channel-forming protein (opp-3C)	1679
SAUSA300_1586	aspartyl-tRNA synthetase (aspS) [6.1.1.12]	1680
SAUSA300_1109	methionyl-tRNA formyltransferase (fmt) [2.1.2.9]	1681
SAUSA300_1948	phi77 ORF069-like protein	1682
SAUSA300_0393	conserved hypothetical protein	1683
SAUSA300_1666	30S ribosomal protein S4 (rpsD)	1684
SAUSA300_1656	universal stress protein family	1685
SAUSA300_2460	acetyltransferase family protein	1686
SAUSA300_1805	RNA methyltransferase	1687
SAUSA300_1545	30S ribosomal protein S20 (rpsT)	1688
SAUSA300_1229	hydrolase, haloacid dehalogenase-like family [3.8.1.-]	1689
SAUSA300_1602	conserved hypothetical protein	1690
pUSA03_0023	transfer complex protein TraO (traO)	1691
SAUSA300_0819	FeS assembly protein SufD (sufD)	1692
SAUSA300_1650	conserved hypothetical protein	1693
SAUSA300_1535	30S ribosomal protein S21 (rpsU)	1694

SAUSA300_0276	putative membrane protein	1695
SAUSA300_1359	polyprenyl synthetase	1696
SAUSA300_2031	conserved hypothetical protein	1697
SAUSA300_0054	hypothetical protein	1698
SAUSA300_2418	conserved hypothetical protein	1699
SAUSA300_0872	conserved hypothetical protein	1700
SAUSA300_2005	conserved hypothetical protein	1701
SAUSA300_1103	DNA-directed RNA polymerase, omega subunit (rpoZ) [2.7.7.6]	1702
SAUSA300_1292	alanine racemase (alr2) [5.1.1.1]	1703
SAUSA300_1288	dihydrodipicolinate synthase (dapA) [4.2.1.52]	1704
SAUSA300_0470	dimethyladenosine transferase (ksgA) [2.1.1.-]	1705
SAUSA300_0752	ATP-dependent Clp protease, proteolytic subunit ClpP (clpP) [3.4.21.92]	1706
SAUSA300_2107	PTS system, mannitol specific IIA component (mtlA) [2.7.1.69]	1707
SAUSA300_0969	phosphoribosylformylglycinamidine synthase (purS)	1708
SAUSA300_1700	polysaccharide biosynthesis protein	1709
SAUSA300_0572	mevalonate kinase (mvk) [2.7.1.36]	1710
SAUSA300_2252	conserved hypothetical protein	1711
SAUSA300_1190	glycerol uptake operon antiterminator regulatory protein (glpP)	1712
SAUSA300_1264	anthranilate phosphoribosyltransferase (trpD) [2.4.2.18]	1713
SAUSA300_0150	transposase, degenerate	1714
SAUSA300_2375	ABC transporter, ATP-binding/permease protein	1715
pUSA01_0001	replication protein	1716
SAUSA300_2264	phosphosugar-binding transcriptional regulator, RpiR family	1717
SAUSA300_1118	conserved hypothetical protein	1718
SAUSA300_2388	2-dehydropantoate 2-reductase (panE) [1.1.1.169]	1719
SAUSA300_0855	Na(+)/H(+) antiporter subunit A (mnhA)	1720
SAUSA300_1572	conserved hypothetical protein	1721
SAUSA300_1436	phiSLT ORF144-like protein, putative lipoprotein	1722
SAUSA300_0322	NADH-dependent flavin oxidoreductase, Oye family [1.-.-.-]	1723
SAUSA300_1051	conserved hypothetical protein	1724
SAUSA300_0140	deoxyribose-phosphate aldolase (deoC) [4.1.2.4]	1725
SAUSA300_0084	conserved hypothetical protein	1726
SAUSA300_2446	conserved hypothetical protein	1727
SAUSA300_0803	transcriptional regulator, Cro/CI family	1728
SAUSA300_0394	FAD/NAD(P)-binding Rossmann fold Superfamily	1729
SAUSA300_1628	lysine-specific permease (lysP)	1730
SAUSA300_1312	acetyltransferase, GNAT family	1731
SAUSA300_0302	conserved hypothetical protein	1732
SAUSA300_0698	para-aminobenzoate synthase, glutamine amidotransferase, component II (pabA) [4.1.3.27]	1733
SAUSA300_0626	teichoic acid biosynthesis protein B (tagB)	1734
SAUSA300_0459	thymidylate kinase (tmk) [2.7.4.9]	1735
SAUSA300_1154	phosphatidate cytidylyltransferase (cdsA) [2.7.7.41]	1736
SAUSA300_1414	phiSLT ORF 78B-like protein	1737
SAUSA300_0784	LysE/YggA family protein	1738
SAUSA300_1244	large conductance mechanosensitive channel protein (mscL)	1739
SAUSA300_2232	acetyltransferase, GNAT family	1740
SAUSA300_1536	conserved hypothetical protein	1741
SAUSA300_1736	conserved hypothetical protein	1742
SAUSA300_2058	ATP synthase F1, beta subunit (atpD) [3.6.3.14]	1743
SAUSA300_0429	PAP2 family protein	1744
SAUSA300_0669	undecaprenol kinase [2.7.1.66]	1745

SAUSA300_2343-s3	respiratory nitrate reductase, alpha subunit [1.7.99.4]	1746
SAUSA300_2174	cobalt transport family protein	1747
SAUSA300_0188	branched-chain amino acid transport system II carrier protein (brnQ)	1748
SAUSA300_0096	conserved hypothetical protein	1749
SAUSA300_0843	conserved hypothetical protein	1750
SAUSA300_1316	methionine-R-sulfoxide reductase (msrB) [1.8.4.-]	1751
SAUSA300_0870-s3	exonuclease RexA (rexA)	1752
SAUSA300_0577	putative transcriptional regulator	1753
SAUSA300_0253	ScdA protein (scdA)	1754
SAUSA300_1115	ribulose-phosphate 3-epimerase (rpe) [5.1.3.1]	1755
SAUSA300_1668	OsmC/Ohr family protein	1756
SAUSA300_0267	transposase	1757
SAUSA300_0422	conserved hypothetical protein	1758
SAUSA300_0257	Antiholin-like protein lrgB.	1759
SAUSA300_0813	conserved hypothetical protein	1760
SAUSA300_0778	conserved hypothetical protein	1761
SAUSA300_0020	DNA-binding response regulator	1762
SAUSA300_1379	putative lipoprotein	1763
SAUSA300_1317	methionine-S-sulfoxide reductase (msrA) [1.8.4.6]	1764
SAUSA300_0109	integral membrane domain protein	1765
SAUSA300_1129	conserved hypothetical protein	1766
SAUSA300_1191	glycerol uptake facilitator (glpF)	1767
SAUSA300_2633	ABC transporter, ATP-binding protein	1768
SAUSA300_0802	conserved hypothetical protein	1769
SAUSA300_0594	alcohol dehydrogenase (adh) [1.1.1.1]	1770
SAUSA300_2203	50S ribosomal protein L4 (rplD)	1771
SAUSA300_0851	Na(+)/H(+) antiporter subunit E (mnhE)	1772
SAUSA300_0983	phosphocarrier protein HPr (ptsH)	1773
SAUSA300_1875	exonuclease	1774
SAUSA300_2236	conserved hypothetical protein	1775
SAUSA300_2246	conserved hypothetical protein	1776
SAUSA300_1747	conserved hypothetical protein	1777
SAUSA300_0990	conserved hypothetical protein	1778
SAUSA300_2234	Inosine-uridine preferring nucleoside hydrolase	1779
SAUSA300_0639	conserved hypothetical protein	1780
SAUSA300_0696	6-pyruvoyl tetrahydrobiopterin synthase-like protein [4.2.3.12]	1781
SAUSA300_0389	GMP synthase (guaA) [6.3.5.2]	1782
SAUSA300_2043	conserved hypothetical protein	1783
SAUSA300_1003	conserved hypothetical protein	1784
SAUSA300_0985	conserved hypothetical protein	1785
SAUSA300_0868	signal peptidase IB (spsB) [3.4.21.89]	1786
SAUSA300_2317	putative zinc-binding dehydrogenase	1787
SAUSA300_0799	integrase (int)	1788
SAUSA300_0366	ribosomal protein S6 (rpsF)	1789
SAUSA300_0689	glycosyl transferase, group 2 family protein	1790
SAUSA300_2547	conserved hypothetical protein	1791
SAUSA300_2025	sigma-B regulation protein (rsbU)	1792
SAUSA300_2593	conserved hypothetical protein	1793
SAUSA300_2369	6-carboxyhexanoate--CoA ligase	1794
SAUSA300_0381	putative NAD(P)H-flavin oxidoreductase	1795
SAUSA300_2532	aspartate 1-decarboxylase (panD) [4.1.1.11]	1796
SAUSA300_2260	inositol monophosphatase family protein [3.1.3.25]	1797
SAUSA300_0652	putative membrane protein	1798
SAUSA300_2182	translation initiation factor IF-1 (infA)	1799
SAUSA300_0539	branched-chain amino acid aminotransferase (ilvE) [2.6.1.42]	1800
SAUSA300_0304	conserved hypothetical protein	1801
SAUSA300_1010	conserved hypothetical protein	1802

SAUSA300_0518	conserved hypothetical protein	1803
SAUSA300_0464	Methyltransferase	1804
SAUSA300_2270	PTS system, arbutin-like IIBC component (glvC) [2.7.1.69]	1805
SAUSA300_0725	conserved hypothetical protein	1806
SAUSA300_1861	conserved hypothetical protein	1807
SAUSA300_0036	conserved hypothetical protein	1808
SAUSA300_1847	conserved hypothetical protein	1809
SAUSA300_0516	conserved hypothetical protein	1810
SAUSA300_2167	conserved hypothetical protein	1811
SAUSA300_0586	conserved hypothetical protein	1812
SAUSA300_1854	regulatory protein RecX	1813
pUSA03_0006	Replication and maintenance protein	1814
SAUSA300_0997	conserved hypothetical protein	1815
SAUSA300_2151	PTS system, lactose-specific IIA component (lacF) [2.7.1.69]	1816
pUSA03_0015	transfer complex protein TraF (traF)	1817
SAUSA300_0900	putative competence protein	1818
SAUSA300_0240	PTS system, galactitol-specific enzyme II, B component [2.7.1.69]	1819
SAUSA300_0673	cobalamin synthesis protein/P47K family protein	1820
SAUSA300_0695	radical activating enzyme family protein	1821
SAUSA300_1420	conserved hypothetical phage protein	1822
SAUSA300_1321	conserved hypothetical protein	1823
SAUSA300_2147	alcohol dehydrogenase, zinc-containing	1824
SAUSA300_0530	ribosomal protein S12 (rpsL)	1825
SAUSA300_1806	putative iron-sulfur cluster-binding protein	1826
SAUSA300_0780	conserved hypothetical protein	1827
SAUSA300_1661	thiamine biosynthesis protein ThiI (thiI)	1828
SAUSA300_0371	conserved hypothetical protein	1829
SAUSA300_0285	conserved hypothetical protein	1830
SAUSA300_0807	conserved hypothetical protein	1831
SAUSA300_2171	30S ribosomal protein S9 (rpsI)	1832
SAUSA300_1670	D-3-phosphoglycerate dehydrogenase (serA) [1.1.1.95]	1833
SAUSA300_1977	conserved hypothetical protein	1834
SAUSA300_1933	hypothetical phage protein	1835
SAUSA300_0306	branched-chain amino acid transport system II carrier protein (brnQ)	1836
SAUSA300_0555	putative hexulose-6-phosphate synthase	1837
SAUSA300_0332	PTS system, IIA component	1838
SAUSA300_2057	ATP synthase F1, epsilon subunit (atpC) [3.6.3.14]	1839
SAUSA300_1552	conserved hypothetical protein	1840
SAUSA300_0979	conserved hypothetical protein	1841
SAUSA300_0604	hydrolase, alpha/beta hydrolase fold family [3.4.11.5]	1842
SAUSA300_0786	OsmC/Ohr family protein	1843
SAUSA300_1472	exodeoxyribonuclease VII, large subunit (xseA) [3.1.11.6]	1844
SAUSA300_2032	K+-transporting ATPase, C subunit (kdpC) [3.6.3.12]	1845
SAUSA300_0542	deoxynucleoside kinase family protein [2.7.1.-2.7.1.113]	1846
SAUSA300_0672	transcriptional regulator, MarR family	1847
SAUSA300_1604	rod shape-determining protein MreD (mreD)	1848
SAUSA300_1238	conserved hypothetical protein	1849
SAUSA300_0966	phosphoribosylaminoimidazole carboxylase, catalytic subunit (purE) [4.1.1.21]	1850
SAUSA300_0641	putative lipase/esterase	1851
SAUSA300_1410	virulence-associated protein E	1852
SAUSA300_1690	putative thioredoxin	1853
SAUSA300_0495	hypothetical protein	1854

SAUSA300_0912	trans-2-enoyl-ACP reductase [1.3.1.9]	1855
SAUSA300_2502	conserved hypothetical protein	1856
SAUSA300_0111	conserved hypothetical protein	1857
SAUSA300_2194	50S ribosomal protein L14 (rplN)	1858
SAUSA300_2410	oligopeptide ABC transporter, permease protein	1859
SAUSA300_0340	NADH-dependent FMN reductase	1860
SAUSA300_1787	HIT family protein	1861
SAUSA300_2443	gluconate kinase (gntK) [2.7.1.12]	1862
SAUSA300_1665	conserved hypothetical protein	1863
SAUSA300_2582	conserved hypothetical protein	1864
SAUSA300_2160	transcriptional regulator, MerR family	1865
SAUSA300_1763	lantibiotic epidermin leader peptide processing serine protease EpiP (epiP) [3.4.21.-]	1866
SAUSA300_2379	putative transporter protein	1867
SAUSA300_0513	glutamyl-tRNA synthetase (gltX) [6.1.1.17]	1868
SAUSA300_0679	conserved hypothetical protein	1869
SAUSA300_1386	phiETA ORF59-like protein	1870
SAUSA300_1734	conserved hypothetical protein	1871
SAUSA300_2624	putative membrane protein	1872
SAUSA300_1337	conserved hypothetical protein	1873
SAUSA300_0930	lipoate-protein ligase A family protein [6.3.4.-]	1874
SAUSA300_2548	conserved hypothetical protein	1875
SAUSA300_0909	pseudouridine synthases, RluA subfamily	1876
SAUSA300_1797	conserved hypothetical protein	1877
SAUSA300_1551	conserved hypothetical protein	1878
SAUSA300_2407	oligopeptide ABC transporter, ATP-binding protein	1879
SAUSA300_2453	ABC transporter, ATP-binding protein	1880
SAUSA300_2300	transcriptional regulator, TetR family	1881
SAUSA300_1914	GntR family regulatory protein	1882
SAUSA300_0953	putative membrane protein	1883
SAUSA300_1174	conserved hypothetical protein	1884
SAUSA300_0149	replication initiation protein, degenerate	1885
SAUSA300_1912	putative membrane protein	1886
SAUSA300_1001	spermidine/putrescine ABC transporter, permease protein (potC)	1887
SAUSA300_1463	conserved hypothetical protein	1888
SAUSA300_1642	D-serine/D-alanine/glycine transporter	1889
SAUSA300_0042	conserved hypothetical protein	1890
SAUSA300_0368	ribosomal protein S18 (rpsR)	1891
SAUSA300_0587	conserved hypothetical protein	1892
SAUSA300_0468	hydrolase, TatD family [3.1.21.-]	1893
SAUSA300_2238	urease, gamma subunit (ureA) [3.5.1.5]	1894
SAUSA300_0957	conserved hypothetical protein	1895
SAUSA300_0442	YibE/F-like protein	1896
SAUSA300_1458	glyoxalase family protein [4.4.1.5]	1897
SAUSA300_2611	histidinol dehydrogenase hisD (hisD) [1.1.1.23]	1898
SAUSA300_0486	polyribonucleotide nucleotidyltransferase [2.7.7.8]	1899
SAUSA300_1743	conserved hypothetical protein	1900
SAUSA300_1523	conserved hypothetical protein	1901
SAUSA300_0097	conserved hypothetical protein	1902
SAUSA300_0169	conserved hypothetical protein	1903
SAUSA300_1941	phi77 ORF003-like protein, phage terminase, large subunit	1904
SAUSA300_0954	transcriptional regulator, MarR family	1905
SAUSA300_2211	putative membrane protein	1906
SAUSA300_1591	adenine phosphoribosyltransferase (apt) [2.4.2.7]	1907
SAUSA300_1303	conserved hypothetical protein	1908
SAUSA300_1767	lantibiotic epidermin biosynthesis protein EpiA (epiA)	1909
SAUSA300_1044	thioredoxin (trx)	1910
SAUSA300_1955	putative endodeoxyribonuclease RusA	1911

SAUSA300_2198	30S ribosomal protein S3 (rpsC)	1912
SAUSA300_0525	ribosomal protein L7/L12 (rplL)	1913
SAUSA300_2417	putative transporter	1914
SAUSA300_0591	acetyltransferase, GNAT family	1915
SAUSA300_1618	hemA concentration negative effector hemX (hemX)	1916
SAUSA300_1708	staphylococcal accessory regulator Rot (rot)	1917
SAUSA300_2028	holo-(acyl-carrier-protein) synthase (acpS) [2.7.8.7]	1918
SAUSA300_1546	DNA polymerase III, delta subunit (holA) [2.7.7.7]	1919
SAUSA300_2376	conserved hypothetical protein	1920
SAUSA300_1013	cell division protein, FtsW/RodA/SpoVE family	1921
SAUSA300_0623	teichoic acid biosynthesis protein (tagA) [2.4.1.187]	1922
SAUSA300_1421	phiSLT ORF122-like protein, DNA polymerase	1923
SAUSA300_2409	oligopeptide ABC transporter, permease protein	1924
SAUSA300_1478	putative lipoprotein	1925
SAUSA300_0565	conserved hypothetical protein	1926
SAUSA300_1761	lantibiotic epidermin immunity protein F (epiE)	1927
SAUSA300_2459	transcriptional regulator, MarR family	1928
SAUSA300_0440	MutT/nudix family protein [3.6.1.-]	1929
SAUSA300_1134	50S ribosomal protein L19 (rplS)	1930
SAUSA300_0564	conserved hypothetical protein	1931
SAUSA300_2518	hydrolase family protein	1932
SAUSA300_1110	ribosomal RNA small subunit methyltransferase B (sun) [2.1.1.-]	1933
SAUSA300_0768	conserved hypothetical protein	1934
SAUSA300_2036	DNA-binding response regulator, KdpE (kdpE) [2.7.3.-]	1935
SAUSA300_0567	conserved hypothetical protein	1936
SAUSA300_0590	conserved hypothetical protein	1937
SAUSA300_0180	integral membrane protein LmrP	1938
SAUSA300_1072	protein mraZ (mraZ)	1939
SAUSA300_1632	conserved hypothetical protein	1940
SAUSA300_0110	transcriptional regulator, GntR family/aminotransferase, class I	1941
SAUSA300_0281	conserved hypothetical protein	1942
SAUSA300_0863	argininosuccinate lyase (argH) [4.3.2.1]	1943
SAUSA300_2242	urease accessory protein UreF (ureF) [3.5.1.5]	1944
SAUSA300_0876	putative membrane protein	1945
SAUSA300_2468	acetyltransferase, GNAT family [2.3.1.-]	1946
SAUSA300_2045	HD domain protein	1947
SAUSA300_1688	phenylalanyl-tRNA synthetase (beta subunit)	1948
pUSA03_0009	putative regulator of transfer genes ArtA	1949
SAUSA300_2631	putative N-acetyltransferase	1950
SAUSA300_0172	conserved hypothetical protein	1951
SAUSA300_1558	5-methylthioadenosine/S-adenosylhomocysteine nucleosidase (mtnN) [3.2.2.16 3.2.2.9]	1952
SAUSA300_1069	conserved hypothetical protein	1953
SAUSA300_0446	glutamate synthase, small subunit (gltD) [1.4.1.13]	1954
SAUSA300_1993	kinase, pfkB family	1955
SAUSA300_2291	sodium/glutamate symporter (gltS)	1956
SAUSA300_2279	LysR family regulatory protein	1957
SAUSA300_1070	acetyltransferase, GNAT family	1958
SAUSA300_0637	dihydroxyacetone kinase, DhaL subunit [2.7.1.-]	1959
SAUSA300_2381	conserved hypothetical protein	1960
SAUSA300_1851	putative membrane protein	1961
SAUSA300_0026	orfX	1962
SAUSA300_1865	DNA-binding response regulator (vraR)	1963
SAUSA300_2368	putative membrane protein	1964
SAUSA300_2437	staphylococcal accessory regulator T (sarT)	1965
SAUSA300_1956	conserved hypothetical phage protein	1966
SAUSA300_0507	transcriptional regulator CtsR (ctsR)	1967
SAUSA300_0753	conserved hypothetical protein	1968

SAUSA300_0854	Na(+)/H(+) antiporter subunit B (mnhB)	1969
SAUSA300_2216	transcriptional regulator, MarR family	1970
SAUSA300_0233	conserved hypothetical protein	1971
SAUSA300_1634	dephospho-CoA kinase (coaE) [2.7.1.24]	1972
SAUSA300_0543	putative deaminase	1973
SAUSA300_0430	conserved hypothetical protein	1974
SAUSA300_0833	conserved hypothetical protein	1975
SAUSA300_1719	arsenate reductase (arsC)	1976
SAUSA300_0847	conserved hypothetical protein	1977
SAUSA300_1427	phiSLT ORF86-like protein	1978
SAUSA300_2243	urease accessory protein UreG (ureG)	1979
SAUSA300_0074	oligopeptide permease, channel-forming protein (opp-3B)	1980
SAUSA300_0121	putative drug transporter	1981
SAUSA300_2073	thymidine kinase (tdk) [2.7.1.21]	1982
SAUSA300_1019	conserved hypothetical protein	1983
SAUSA300_1872	conserved hypothetical protein	1984
SAUSA300_1437	phiSLT ORF204-like protein	1985
SAUSA300_2186	50S ribosomal protein L30 (rpmD)	1986
SAUSA300_0643	acetyltransferase, GNAT family	1987
SAUSA300_2137	conserved hypothetical protein	1988
SAUSA300_1582	conserved hypothetical protein	1989
SAUSA300_2155	galactose-6-phosphate isomerase (lacA) [5.3.1.26]	1990
SAUSA300_1968	putative phage transcriptional regulator	1991
SAUSA300_2327	conserved hypothetical protein	1992
SAUSA300_1859	conserved hypothetical protein	1993
SAUSA300_2188	50S ribosomal protein L18 (rplR)	1994
pUSA03_0019	transfer protein complex TraJ (traJ)	1995
SAUSA300_1348	polyA polymerase [2.7.7.19]	1996
SAUSA300_0129	Acetoin(diacetyl) reductase [1.1.1.5]	1997
SAUSA300_1745	conserved hypothetical protein	1998
SAUSA300_1939	phi77 ORF015-like protein, putative protease	1999
SAUSA300_0386	xanthine phosphoribosyltransferase (xpt) [2.4.2.-]	2000
SAUSA300_0261	conserved hypothetical protein	2001
SAUSA300_1936	conserved hypothetical phage protein	2002
SAUSA300_2197	50S ribosomal protein L16 (rplP)	2003
SAUSA300_0083	putative membrane protein	2004
SAUSA300_2140	conserved hypothetical protein	2005
SAUSA300_0246	putative alcohol dehydrogenase	2006
SAUSA300_2422	oxidoreductase, short-chain dehydrogenase/reductase family	2007
SAUSA300_1537	conserved hypothetical protein	2008
SAUSA300_2592	conserved hypothetical protein	2009
SAUSA300_0195	RpiR family transcriptional regulator	2010
SAUSA300_0613	putative Na+/H+ antiporter, MnhD component	2011
SAUSA300_2290	putative 3-methyladenine DNA glycosylase	2012
SAUSA300_0289	conserved hypothetical protein	2013
pUSA03_0025	conserved hypothetical protein	2014
SAUSA300_0474	putative endoribonuclease L-PSP	2015
SAUSA300_2037	ATP-dependent RNA helicase	2016
SAUSA300_1455	transcriptional regulator, AraC family	2017
SAUSA300_0472	4-diphosphocytidyl-2C-methyl-D-erythritol kinase (ispE) [2.7.1.148]	2018
SAUSA300_1435	phiSLT ORF153-like protein	2019
SAUSA300_1732	putative transposase	2020
SAUSA300_1901	aldehyde dehydrogenase (aldA2) [1.2.1.3]	2021
SAUSA300_1589	D-tyrosyl-tRNA (Tyr) deacylase (dtd) [3.1.-.-]	2022
SAUSA300_1871	conserved hypothetical protein	2023
SAUSA300_2233	BioY family protein	2024
SAUSA300_1971	phi77 ORF017-like protein	2025
SAUSA300_1790	foldase protein PrsA precursor (prsA) [5.2.1.8]	2026

SAUSA300_1724	abortive infection protein family	2027
SAUSA300_1081	conserved hypothetical protein	2028
SAUSA300_2620	conserved hypothetical protein	2029
SAUSA300_2613	conserved hypothetical protein	2030
SAUSA300_2225	molybdenum cofactor biosynthesis protein C (moaC)	2031
SAUSA300_1033	iron/heme permease	2032
SAUSA300_1629	threonyl-tRNA synthetase (thrS) [6.1.1.3]	2033
SAUSA300_2210	probable glucose uptake protein (glcU)	2034
SAUSA300_2206	conserved hypothetical protein	2035
SAUSA300_1733	conserved hypothetical protein	2036
SAUSA300_0569	conserved hypothetical protein	2037
SAUSA300_0444	LysR family regulatory protein (gltC)	2038
SAUSA300_0904	protozoan/cyanobacterial globin family protein	2039
SAUSA300_1424	conserved hypothetical phage protein	2040
SAUSA300_1981	phage terminase family protein	2041
SAUSA300_0998	conserved hypothetical protein	2042
SAUSA300_1462	conserved hypothetical protein	2043
SAUSA300_0154	capsular polysaccharide biosynthesis protein Cap5C (cap5C) [3.1.3.48]	2044
SAUSA300_2461	glyoxalase family protein [1.13.11.39]	2045
SAUSA300_0343	acetyltransferase, GNAT family	2046
SAUSA300_1242	exonuclease SbcD (sbcD) [3.1.-.-]	2047
SAUSA300_0735	competence protein F	2048
SAUSA300_2269	conserved hypothetical protein	2049
SAUSA300_1501	putative competence protein ComG	2050
SAUSA300_2636	integrase/recombinase	2051
SAUSA300_2002	glycoprotein endopeptidase	2052
SAUSA300_2054	(3R)-hydroxymyristoyl-[acyl carrier protein] dehydratase (fabZ) [4.2.1.-]	2053
SAUSA300_2088	S-ribosylhomocysteinase (luxS) [3.13.1.-]	2054
SAUSA300_2177	50S ribosomal protein L17 (rplQ)	2055
SAUSA300_1273	oligopeptide permease, ATP-binding protein (opp-2F)	2056
SAUSA300_1281	phosphate ABC transporter, permease protein PstA (pstA)	2057
SAUSA300_0330	putative transport protein SgaT	2058
SAUSA300_0390	conserved hypothetical protein	2059
SAUSA300_0745	putative acetyltransferase	2060
SAUSA300_0991	peptide deformylase (def) [3.5.1.88]	2061
SAUSA300_2521	conserved hypothetical protein	2062
SAUSA300_0528	DNA-directed RNA polymerase, beta subunit (rpoC) [2.7.7.6]	2063
SAUSA300_0788	nitroreductase family protein	2064
SAUSA300_2618	conserved hypothetical protein	2065
SAUSA300_0714	Integral membrane protein	2066
SAUSA300_2059	ATP synthase F1, gamma subunit (atpG) [3.6.3.14]	2067
SAUSA300_1237	LexA repressor (lexA) [3.4.21.88]	2068
SAUSA300_1397	phiSLT ORF213-like protein, major tail protein	2069
SAUSA300_1760	lantibiotic epidermin immunity protein F (epiG)	2070
SAUSA300_2346	nitrite reductase [NAD(P)H], large subunit (nirB) [1.7.1.4]	2071
SAUSA300_1989	accessory gene regulator protein B (agrB)	2072
SAUSA300_0095	transcriptional regulator, LysR family domain protein	2073
SAUSA300_1368	L-asparaginase (ansA) [3.5.1.1]	2074
SAUSA300_0391	conserved hypothetical protein	2075
SAUSA300_1500	putative competence protein ComYC	2076
SAUSA300_2570	arginine deiminase (arcA) [3.5.3.6]	2077
SAUSA300_1000	spermidine/putrescine ABC transporter, permease protein (potB)	2078

SAUSA300_0647	ABC transporter, ATP-binding protein	2079
SAUSA300_1300	branched-chain amino acid transport system II carrier protein (brnQ)	2080
SAUSA300_1934	phi77 ORF020-like protein, phage major tail protein	2081
SAUSA300_1267	tryptophan synthase, beta subunit (trpB) [4.2.1.20]	2082
SAUSA300_1241	conserved hypothetical protein	2083
SAUSA300_0526	Methyltransferase small domain	2084
SAUSA300_1722	conserved hypothetical protein	2085
SAUSA300_1049	glutamate racemase (murI) [5.1.1.3]	2086
SAUSA300_1868	conserved hypothetical protein	2087
SAUSA300_2479	Holin-like protein cidA (cidA)	2088
SAUSA300_1566	conserved hypothetical protein	2089
SAUSA300_2448	putative membrane protein	2090
SAUSA300_0182	4-phosphopantetheinyl transferase superfamily protein	2091
SAUSA300_1751	type I restriction-modification enzyme, S subunit (hsdS)	2092
SAUSA300_0159	capsular polysaccharide biosynthesis protein Cap5H (cap5H)	2093
SAUSA300_2577	mannose-6-phosphate isomerase, class I (manA) [5.3.1.8]	2094
SAUSA300_0144	phosphonate ABC transporter, ATP-binding protein (phnC)	2095
SAUSA300_1331	alanine dehydrogenase (ald) [1.4.1.1]	2096
SAUSA300_0108	antigen, 67 kDa	2097
SAUSA300_1795	conserved hypothetical protein	2098
SAUSA300_0131	putative Bacterial sugar transferase	2099
SAUSA300_0482	polysaccharide biosynthesis protein	2100
SAUSA300_0178	conserved hypothetical protein	2101
SAUSA300_1515	ABC transporter, permease protein	2102
SAUSA300_1744	conserved hypothetical protein	2103
SAUSA300_0176	ABC transporter, permease protein	2104
SAUSA300_2104	glucosamine--fructose-6-phosphate aminotransferase (isomerizing) (glmS) [2.6.1.16]	2105
SAUSA300_2614	putative lipoprotein	2106
SAUSA300_1261	putative glutamyl aminopeptidase	2107
SAUSA300_2021	S1 RNA binding domain protein	2108
SAUSA300_1132	16S rRNA processing protein RimM (rimM)	2109
SAUSA300_0127	conserved hypothetical protein	2110
SAUSA300_1964	conserved hypothetical phage protein	2111
SAUSA300_0070	putative lysophospholipase	2112
SAUSA300_1898	conserved hypothetical protein	2113
SAUSA300_2162	M23/M37 peptidase domain protein	2114
SAUSA300_1504	putative competence protein ComGA	2115
SAUSA300_1750-s3	conserved hypothetical protein	2116
SAUSA300_0718	iron compound ABC transporter, permease	2117
SAUSA300_2154	galactose-6-phosphate isomerase (lacB) [5.3.1.26]	2118
SAUSA300_2405	putative membrane protein	2119
pUSA02_0001	tetracycline resistance protein	2120
SAUSA300_0266	conserved hypothetical protein	2121
SAUSA300_0427	conserved hypothetical protein	2122
SAUSA300_0583	conserved hypothetical protein	2123
SAUSA300_1278	oligoendopeptidase F (pepF) [3.4.24.-]	2124
SAUSA300_0559	putative substrate--CoA ligase	2125
SAUSA300_2313	L-lactate permease	2126
SAUSA300_2318	acetyltransferase, GNAT family	2127
SAUSA300_2456	putative membrane protein	2128
SAUSA300_1313	carboxyl-terminal protease (ctpA) [3.4.21.-]	2129
SAUSA300_1353	conserved hypothetical protein	2130
SAUSA300_0312	indigoidine synthase family protein	2131
SAUSA300_0898	Regulatory protein spx (spxA)	2132

SAUSA300_1524	CBS domain pair protein	2133
pUSA03_0036	conserved hypothetical protein	2134
SAUSA300_2640	putative transcriptional regulator	2135
SAUSA300_1621	ATP-dependent Clp protease, ATP-binding subunit ClpX (clpX)	2136
SAUSA300_0968	phosphoribosylaminoimidazole-succinocarboxamide synthase (purC) [6.3.2.6]	2137
SAUSA300_2320	conserved hypothetical protein	2138
SAUSA300_0008	histidine ammonia-lyase (hutH) [4.3.1.3]	2139
SAUSA300_2138	conserved hypothetical protein	2140
SAUSA300_1497	glycine dehydrogenase, subunit 1 (glycine cleavage system P protein) [1.4.4.2]	2141
SAUSA300_0186	N-acetyl-gamma-glutamyl-phosphate reductase (argC) [1.2.1.38]	2142
SAUSA300_0046	conserved hypothetical protein	2143
SAUSA300_0181	non-ribosomal peptide synthetase	2144
SAUSA300_0094	conserved hypothetical protein	2145
SAUSA300_1530	conserved hypothetical protein	2146
SAUSA300_1954	phiPVL ORF050-like protein	2147
SAUSA300_2169	conserved hypothetical protein	2148
SAUSA300_1991	accessory gene regulator protein C (agrC)	2149
SAUSA300_1326	putative cell wall enzyme EbsB	2150
SAUSA300_1022	conserved hypothetical protein	2151
SAUSA300_0918	conserved hypothetical protein	2152
SAUSA300_0701	conserved hypothetical protein TIGR00370	2153
SAUSA300_2553	putative siroheme synthase	2154
SAUSA300_1959	phiPVL ORF044-like protein	2155
SAUSA300_1930-s3	phi77 ORF001-like protein, phage tail tape measure protein	2156
SAUSA300_2629	conserved hypothetical protein	2157
SAUSA300_1262	anthranilate synthase component I (trpE) [4.1.3.27]	2158
SAUSA300_2485	methylated DNA-protein cysteine methyltransferase [2.1.1.63]	2159
SAUSA300_1232	catalase [1.11.1.6]	2160
SAUSA300_1148	GTP-sensing transcriptional pleiotropic repressor CodY (codY)	2161
pUSA03_0005	transposase	2162
SAUSA300_2525	conserved hypothetical protein	2163
SAUSA300_1984	putative membrane protein	2164
SAUSA300_1374	conserved hypothetical protein	2165
SAUSA300_2090	deoxyribose-phosphate aldolase (deoC) [4.1.2.4]	2166
SAUSA300_2009	ketol-acid reductoisomerase (ilvC) [1.1.1.86]	2167
SAUSA300_0078	ATPase copper transport (copA) [3.6.3.4]	2168
SAUSA300_2179	30S ribosomal protein S11 (rpsK)	2169
SAUSA300_2328	conserved hypothetical protein	2170
SAUSA300_2509	transcriptional regulator, TetR family	2171
SAUSA300_2228	molybdenum ABC transporter, ATP-binding protein ModC (modC) [3.6.3.29]	2172
SAUSA300_1907	conserved hypothetical protein	2173
SAUSA300_0902	oligoendopeptidase F (pepF) [3.4.24.-]	2174
SAUSA300_2033	K+-transporting ATPase, B subunit (kdpB) [3.6.3.12]	2175
SAUSA300_2373	dethiobiotin synthase (bioD) [6.3.3.3]	2176
SAUSA300_2394	conserved hypothetical protein	2177
SAUSA300_0103	staphylococcus tandem lipoprotein	2178
SAUSA300_0435	ABC transporter, ATP-binding protein	2179
SAUSA300_0436	ABC transporter, permease protein	2180
SAUSA300_1660	putative membrane protein	2181
SAUSA300_1999	redox-sensing transcriptional repressor rex (rex)	2182
SAUSA300_0349	conserved hypothetical protein	2183
SAUSA300_1567	transcription elongation factor GreA (greA)	2184
SAUSA300_0817	putative membrane protein	2185

SAUSA300_0016	replicative DNA helicase (dnaB) [3.6.1.-]	2186
SAUSA300_0052	hypothetical protein	2187
SAUSA300_1608	DNA repair protein RadC	2188
SAUSA300_0433	cysteine synthase/cystathionine beta-synthase (cysM) [4.2.99.8]	2189
SAUSA300_1568	uridine kinase (udk) [2.7.1.48]	2190
SAUSA300_0445-s3	glutamate synthase, large subunit (gltB) [1.4.1.13]	2191
SAUSA300_0822	FeS assembly protein SufB (sufB)	2192
SAUSA300_1513	superoxide dismutase (Mn/Fe family) [1.15.1.1]	2193
SAUSA300_1565	putative urea amidolyase	2194
SAUSA300_0250	alcohol dehydrogenase, zinc-containing [1.1.1.1]	2195
SAUSA300_1224	conserved hypothetical protein	2196
SAUSA300_0582	conserved hypothetical protein	2197
SAUSA300_1555	shikimate 5-dehydrogenase (aroE) [1.1.1.25]	2198
SAUSA300_0856	conserved hypothetical protein	2199
SAUSA300_2563	putative transcriptional regulator	2200
SAUSA300_0605	staphylococcal accessory regulator A (sarA)	2201
SAUSA300_2026	PemK family protein	2202
SAUSA300_1655	alanine dehydrogenase (ald) [1.4.1.1]	2203
SAUSA300_0907	GTP pyrophosphokinase [2.7.6.5]	2204
SAUSA300_1308	DNA-binding response regulator (arlR)	2205
SAUSA300_2606	imidazole glycerol phosphate synthase subunit hisF (hisF)	2206
SAUSA300_2011	3-isopropylmalate dehydrogenase (leuB) [1.1.1.85]	2207
SAUSA300_0521	transcription termination/antitermination factor NusG (nusG)	2208
SAUSA300_0824	conserved hypothetical protein	2209
SAUSA300_1799	putative sensor histidine kinase	2210
SAUSA300_0115	iron compound ABC transporter, permease protein SirC (sirC)	2211
SAUSA300_0705	ATP-dependent DNA helicase RecQ (recQ) [3.6.1.-]	2212
SAUSA300_0102	staphylococcus tandem lipoprotein	2213
SAUSA300_2129	putative hemolysin III	2214
SAUSA300_1645	6-phosphofructokinase (pfkA) [2.7.1.11]	2215
SAUSA300_2022	RNA polymerase sigma-37 factor (rpoF)	2216
SAUSA300_2168	conserved hypothetical protein	2217
SAUSA300_2552	citrate transporter, permease protein	2218
SAUSA300_1675	tyrosyl-tRNA synthetase (tyrS) [6.1.1.1]	2219
SAUSA300_2350	conserved hypothetical protein	2220
SAUSA300_0732	conserved hypothetical protein	2221
SAUSA300_0797	ABC transporter permease protein	2222
SAUSA300_2630	high-affinity nickel-transporter (nixA)	2223
SAUSA300_2237	putative urea transporter	2224
SAUSA300_0750	conserved hypothetical protein	2225
SAUSA300_2599	intercellular adhesion operon transcription regulator, TetR family (tetR)	2226
SAUSA300_0558	putative proline/betaine transporter	2227
SAUSA300_1282	phosphate ABC transporter, permease protein PstC (pstC)	2228
SAUSA300_1176	CDP-diacylglycerol--glycerol-3-phosphate 3-phosphatidyltransferase (pgsA) [2.7.8.5]	2229
SAUSA300_2438	staphylococcal accessory regulator U (sarU)	2230
SAUSA300_0791	glycine cleavage system H protein (gcvH)	2231
SAUSA300_0674	oxidoreductase, aldo/keto reductase family [1.-.-.-]	2232
SAUSA300_1207	conserved hypothetical protein	2233
SAUSA300_2602	intercellular adhesion protein C (icaC)	2234
SAUSA300_1804	conserved hypothetical protein	2235
SAUSA300_2558	sensor histidine kinase [2.7.3.-]	2236
SAUSA300_0531	30S ribosomal protein S7	2237
SAUSA300_2616	cobalt transport family protein	2238
SAUSA300_0848	conserved hypothetical protein	2239

SAUSA300_0584	conserved hypothetical protein	2240
SAUSA300_0622	putative membrane protein	2241
SAUSA300_0269	Ccholoylglycine hydrolase family protein [3.5.1.24]	2242
SAUSA300_1887	geranylgeranylglyceryl phosphate synthase family protein, PcrB (pcrB)	2243
SAUSA300_1453	ribonuclease Z [3.1.26.11]	2244
SAUSA300_1490	translation elongation factor P (efp)	2245
SAUSA300_0362	conserved hypothetical protein	2246
SAUSA300_2608	imidazole glycerol phosphate synthase subunit hisH (hisH) [2.4.2.-]	2247
SAUSA300_1201	glutamine synthetase, type I (glnA) [6.3.1.2]	2248
SAUSA300_2262	putative membrane protein	2249
SAUSA300_2150	PTS system, lactose-specific IIBC component (lacE) [2.7.1.69]	2250
SAUSA300_1782	ferrochelatase (hemH) [4.99.1.1]	2251
SAUSA300_1735	O-succinylbenzoic acid synthetase (menC) [4.2.1.-]	2252
SAUSA300_1727	putative transposase, degenerate	2253
SAUSA300_0731	glycosyl transferase, group 4 family protein [2.7.8.-]	2254
SAUSA300_0147	5 nucleotidase family protein [3.1.4.16]	2255
SAUSA300_2189	50S ribosomal protein L6 (rplF)	2256
SAUSA300_1657	acetate kinase (ackA) [2.7.2.1]	2257
SAUSA300_0988	potassium uptake protein (trkA)	2258
SAUSA300_1491	proline dipeptidase [3.4.13.9]	2259
SAUSA300_0563	uracil-DNA glycosylase (ung) [3.2.2.-]	2260
SAUSA300_1036	RNA methyltransferase, TrmH family	2261
SAUSA300_1194	hydrolase, alpha/beta hydrolase fold family	2262
SAUSA300_1553	nicotinate (nicotinamide) nucleotide adenylyltransferase (nadD) [2.7.7.18]	2263
SAUSA300_1577	TPR domain protein	2264
SAUSA300_2543	conserved hypothetical protein	2265
SAUSA300_0114	staphylococcal accessory regulator	2266
SAUSA300_1498	aminomethyltransferase (glycine cleavage system T protein) (gcvT) [2.1.2.10]	2267
SAUSA300_1869	methionine aminopeptidase (map) [3.4.11.18]	2268
SAUSA300_1266	N-(5phosphoribosyl)anthranilate isomerase (trpF) [5.3.1.24]	2269
SAUSA300_1050	non-canonical purine NTP pyrophosphatase, rdgB/HAM1 family	2270
SAUSA300_1093	aspartate carbamoyltransferase (pyrB) [2.1.3.2]	2271
SAUSA300_1329	amino acid permease	2272
SAUSA300_1228	homoserine kinase (thrB) [2.7.1.39]	2273
SAUSA300_1083	conserved hypothetical protein	2274
SAUSA300_2414	conserved hypothetical protein	2275
SAUSA300_1509	peptidase, rhomboid family	2276
SAUSA300_1570	peptidase, U32 family [3.4.-.-]	2277
SAUSA300_0726	glycerate kinase family protein [2.7.1.31]	2278
SAUSA300_2507	similar to regulatory protein	2279
SAUSA300_1218	ABC transporter, permease protein	2280
SAUSA300_2027	alanine racemase (alr) [5.1.1.1]	2281
pUSA03_0024	transposase	2282
SAUSA300_0972	amidophosphoribosyltransferase (purF) [2.4.2.14]	2283
SAUSA300_1336	conserved hypothetical protein	2284
SAUSA300_2126	drug resistance transporter, EmrB/QacA subfamily	2285
SAUSA300_0134	polysaccharide extrusion protein	2286
SAUSA300_2472	putative membrane protein	2287
SAUSA300_2310	conserved hypothetical protein	2288
SAUSA300_1198	putative GTP-binding protein	2289
SAUSA300_0245	2-C-methyl-D-erythritol 4-phosphate cytidylyltransferase [2.7.7.60]	2290
SAUSA300_1338	conserved hypothetical protein	2291
SAUSA300_2314	conserved hypothetical protein	2292

SAUSA300_2628	RarD protein (rarD)	2293
SAUSA300_2175	ABC transporter, ATP-binding protein	2294
SAUSA300_2399	ABC transporter, ATP-binding protein	2295
SAUSA300_1785	putative ABC transporter protein EcsB	2296
SAUSA300_1263	anthranilate synthase, glutamine amidotransferase, component II (trpG) [4.1.3.27]	2297
SAUSA300_1015	cytochrome oxidase assembly protein (ctaA)	2298
SAUSA300_0310	perfringolysin O regulator protein (pfoR)	2299
SAUSA300_2097	conserved hypothetical protein	2300
SAUSA300_2610	histidinol-phosphate aminotransferase hisC (hisC)	2301
SAUSA300_1146	ATP-dependent protease hslV (hslV) [3.4.99.-]	2302
SAUSA300_1121	conserved hypothetical protein	2303
SAUSA300_1902	conserved hypothetical protein	2304
SAUSA300_0354	low temperature requirement protein LtrA (ltrA)	2305
SAUSA300_0357	5-methyltetrahydropteroyltriglutamate--homocysteine S-methyltransferase (metE) [2.1.1.14]	2306
SAUSA300_1173	putative acetoacetyl-CoA reductase	2307
SAUSA300_1151	uridylate kinase (pyrH) [2.7.4.-]	2308
SAUSA300_2347	nitrite reductase transcriptional regulator NirR (nirR)	2309
SAUSA300_1214	conserved hypothetical protein	2310
SAUSA300_0709	5(3)-deoxyribonucleotidase [3.1.3.-]	2311
SAUSA300_2426	conserved hypothetical protein	2312
SAUSA300_0265	putative ribose operon repressor	2313
SAUSA300_1123	malonyl CoA-acyl carrier protein transacylase (fabD) [2.3.1.39]	2314
SAUSA300_0138	purine nucleoside phosphorylase (deoD) [2.4.2.1]	2315
SAUSA300_2303	transcriptional regulator TcaR (tcaR)	2316
SAUSA300_2549	choline/carnitine/betaine transporter, BCCT family (bccT)	2317
SAUSA300_0153	capsular polysaccharide biosynthesis protein Cap5B (cap5B) [2.7.1.112]	2318
SAUSA300_0818	FeS assembly ATPase SufC (sufC)	2319
SAUSA300_2551	anaerobic ribonucleotide reductase, large subunit (nrdD) [1.17.4.2]	2320
SAUSA300_2585	accessory secretory protein Asp3	2321
SAUSA300_0222	putative membrane protein	2322
SAUSA300_0890	oligopeptide ABC transporter, ATP-binding protein (oppF)	2323
SAUSA300_2261	conserved hypothetical protein	2324
SAUSA300_0432	sodium dependent transporter	2325
SAUSA300_0191	PTS system, glucose-specific IIBC component domain protein (ptsG) [2.7.1.69]	2326
SAUSA300_1561	putative membrane protein	2327
SAUSA300_0072	hypothetical protein	2328
SAUSA300_0895	oligopeptide ABC transporter, permease protein (oppB)	2329
SAUSA300_2284	conserved hypothetical protein	2330
SAUSA300_1142	DNA protecting protein DprA (dprA)	2331
SAUSA300_0217	DNA-binding response regulator, AraC family	2332
SAUSA300_1309	transposase, IS200 family	2333
SAUSA300_0314	sodium:solute symporter family protein	2334
SAUSA300_0976	phosphoribosylamine--glycine ligase (purD) [6.3.4.13]	2335
SAUSA300_0069	IS431 transposase, authentic frameshift	2336
SAUSA300_0384	conserved hypothetical protein	2337
SAUSA300_2306	ABC transporter, ATP-binding protein	2338
SAUSA300_0517	RNA methyltransferase, TrmH family, group 3	2339
SAUSA300_2190	30S ribosomal protein S8 (rpsH)	2340
SAUSA300_2128	putative drug transporter	2341
SAUSA300_2064	ATP synthase F0, A subunit (atpB) [3.6.3.14]	2342

SAUSA300_1328	putative drug transporter	2343
SAUSA300_1687	FtsK/SpoIIIE family protein	2344
SAUSA300_2482	conserved hypothetical protein	2345
SAUSA300_0107	Na/Pi cotransporter family protein	2346
SAUSA300_0268	putative drug transporter	2347
SAUSA300_2588	preprotein translocase, SecY protein	2348
SAUSA300_2416	similar to glucose 1-dehydrogenase [1.1.1.47]	2349
SAUSA300_0206	flavodoxin family protein	2350
SAUSA300_0712	amino acid/peptide transporter (Peptide:H+ symporter)	2351
SAUSA300_0609	phage integrase family protein	2352
SAUSA300_2490	regulatory protein, TetR family	2353
SAUSA300_0852	Na(+)/H(+) antiporter subunit D (mnhD)	2354
SAUSA300_0684	fructose 1-phosphate kinase (fruB) [2.7.1.56]	2355
SAUSA300_0635	ferrichrome transport permease protein fhuG (fhuG)	2356
SAUSA300_0062	ornithine carbamoyltransferase (arcB) [2.1.3.3]	2357
SAUSA300_1226	homoserine dehydrogenase [1.1.1.3]	2358
SAUSA300_2324	PTS system, sucrose-specific IIBC component [2.7.1.69]	2359
SAUSA300_1450	oxidoreductase, aldo/keto reductase family	2360
SAUSA300_0341	putative membrane protein	2361
SAUSA300_1393-s4	phiSLT ORF2067-like protein, phage tail tape measure protein	2362
SAUSA300_1080	cell division protein ftsZ (ftsZ)	2363
SAUSA300_0317	conserved hypothetical protein	2364
SAUSA300_2445	transcriptional regulator, MerR family	2365
SAUSA300_1996	ammonium transporter (amt)	2366
SAUSA300_2607	phosphoribosylformimino-5-aminoimidazole carboxamide ribotide isomerase hisA (hisA) [5.3.1.16]	2367
SAUSA300_2357	ABC transporter, ATP-binding protein	2368
SAUSA300_1243-s2	exonuclease SbcC (sbcC)	2369
SAUSA300_0215	conserved hypothetical protein	2370
SAUSA300_0037	cassette chromosome recombinase B (ccrB)	2371
SAUSA300_0650	phosphate transporter family protein	2372
SAUSA300_2255	monooxygenase family protein [1.14.13.-]	2373
SAUSA300_0271	ABC transporter, ATP-binding protein	2374
SAUSA300_0986	cytochrome D ubiquinol oxidase, subunit I [1.10.3.-]	2375
SAUSA300_0041	conserved hypothetical protein	2376
SAUSA300_1850	conserved hypothetical protein	2377
SAUSA300_0122	siderophore biosynthesis protein, IucA/IucC family	2378
SAUSA300_2496	D-isomer specific 2-hydroxyacid dehydrogenase family protein [1.1.1.28]	2379
SAUSA300_0238	transcriptional antiterminator, BglG family	2380
SAUSA300_0118	pyridoxal-phosphate dependent enzyme superfamily	2381
SAUSA300_2385	putative membrane protein	2382
SAUSA300_0116	iron compound ABC transporter, permease protein SirB (sirB)	2383
SAUSA300_1085	conserved hypothetical protein	2384
SAUSA300_2523	conserved hypothetical protein	2385
SAUSA300_0506	pyrimidine nucleoside transport protein (nupC)	2386
SAUSA300_1182	pyruvate ferredoxin oxidoreductase, alpha subunit [1.2.7.3]	2387
SAUSA300_0742	excinuclease ABC, A subunit (uvrA)	2388
SAUSA300_1457	maltose operon transcriptional repressor (malR)	2389
SAUSA300_1620	probable GTP-binding protein engB	2390
SAUSA300_1451	oxidoreductase, short-chain dehydrogenase/reductase family	2391
SAUSA300_2071	modification methylase, HemK family [2.1.1.-]	2392

SAUSA300_1809	putative membrane protein	2393
SAUSA300_0061	carbamate kinase (arcC) [2.7.2.2]	2394
SAUSA300_1531	phosphate starvation-induced protein, PhoH family (phoH)	2395
SAUSA300_2559	DNA-binding response regulator	2396
pUSA03_0020	transfer complex protein TraK (traK)	2397
SAUSA300_0736	ribosomal subunit interface protein (yfiA)	2398
SAUSA300_1183	pyruvate ferredoxin oxidoreductase, beta subunit	2399
SAUSA300_1672	phosphotransferase system, N-acetylglucosamine-specific IIBC component (nagE) [2.7.1.69]	2400
SAUSA300_1120	ATP-dependent DNA helicase RecG (recG) [3.6.1.-]	2401
SAUSA300_0143	phosphonate ABC transporter, permease protein (phnE)	2402
SAUSA300_2612	ATP phosphoribosyltransferase hisG (hisG) [2.4.2.17]	2403
SAUSA300_0466	conserved hypothetical protein	2404
SAUSA300_1065	exfoliative toxin A	2405
SAUSA300_0557	HAD-superfamily hydrolase, subfamily IA, variant 1	2406
SAUSA300_0218	sensor histidine kinase family protein [2.7.3.-]	2407
SAUSA300_1542	heat-inducible transcription repressor HrcA (hrcA)	2408
SAUSA300_0740	conserved hypothetical protein	2409
SAUSA300_0720	putative iron compound ABC transporter, ATP-binding protein	2410
SAUSA300_1916	aminotransferase	2411
SAUSA300_1716	conserved hypothetical protein	2412
SAUSA300_1063	carbamate kinase (arcC) [2.7.2.2]	2413
SAUSA300_2494	copper-translocating P-type ATPase	2414
SAUSA300_0227	acyl-CoA dehydrogenase FadD (fadD) [1.3.99.7]	2415
SAUSA300_1408	phage helicase	2416
SAUSA300_0119	ornithine cyclodeaminase [4.3.1.12]	2417
SAUSA300_0646	sensor histidine kinase [2.7.3.-]	2418
SAUSA300_2454	membrane spanning protein	2419
SAUSA300_1510	5-formyltetrahydrofolate cyclo-ligase subfamily	2420
SAUSA300_1786	ABC transporter, ATP-binding protein EcsA	2421
SAUSA300_1646	acetyl-CoA carboxylase, carboxyl transferase, alpha subunit (accA) [6.4.1.2]	2422
SAUSA300_2342	respiratory nitrate reductase, beta subunit (narH) [1.7.99.4]	2423
SAUSA300_2139	putative transporter	2424
SAUSA300_2281	formimidoylglutamase (hutG) [3.5.3.8]	2425
SAUSA300_0175	putative lipoprotein	2426
SAUSA300_1073	S-adenosyl-methyltransferase MraW (mraW) [2.1.1.-]	2427
SAUSA300_2277	imidazolonepropionase (hutI) [3.5.2.7]	2428
SAUSA300_0687	putative hemolysin	2429
SAUSA300_2568	arginine/ornithine antiporter (arcD)	2430
SAUSA300_1283	phosphate ABC transporter, phosphate-binding protein PstS (pstS)	2431
SAUSA300_0571	lipoate-protein ligase A family protein	2432
SAUSA300_0291	putative membrane protein	2433
SAUSA300_1020	Glycerophosphoryl diester phosphodiesterase family protein	2434
SAUSA300_1791	cmp-binding-factor 1 (cbf1)	2435
SAUSA300_2082	DNA-directed RNA polymerase, delta subunit (rpoE) [2.7.7.6]	2436
SAUSA300_2583	putative glycosyl transferase	2437
SAUSA300_0461	DNA polymerase III delta subunit (holB) [2.7.7.7]	2438
SAUSA300_0978	ABC transporter, ATP-binding protein	2439
SAUSA300_2557	ABC transporter protein	2440
SAUSA300_0142	phosphonate ABC transporter, permease protein (phnE)	2441

SAUSA300_0629	penicillin-binding protein 4 (pbp4) [3.4.16.4]	2442
SAUSA300_1714	riboflavin synthase, alpha subunit (ribE) [2.5.1.9]	2443
SAUSA300_2333	nitrite extrusion protein (narK)	2444
pUSA03_0003-s1	Isoleucyl-tRNA synthetase, mupirocin resistant protein (ileS) [6.1.1.5]	2445
SAUSA300_1995	sucrose operon repressor (scrR)	2446
SAUSA300_2135	iron compound ABC transporter, permease protein	2447
SAUSA300_0124	HPCH/HPAI aldolase family protein	2448
SAUSA300_0012	putative homoserine O-acetyltransferase	2449
SAUSA300_2383	amino acid permease	2450
pUSA03_0008	transposase	2451
SAUSA300_1970	putative exonuclease	2452
SAUSA300_2406	putative transporter	2453
pUSA03_0029	conserved hypothetical protein	2454
SAUSA300_2007	acetolactate synthase, large subunit (ilvB) [2.2.1.6]	2455
SAUSA300_0273	putative membrane protein	2456
SAUSA300_0313	putative nucleoside permease NupC	2457
SAUSA300_1293	diaminopimelate decarboxylase (lysA) [4.1.1.20]	2458
SAUSA300_1101	putative fibronectin/fibrinogen binding protein	2459
SAUSA300_2201	50S ribosomal protein L2 (rplB)	2460
SAUSA300_1580	bacterial luciferase family protein	2461
SAUSA300_2103	ABC transporter, ATP-binding protein	2462
SAUSA300_2356	fmhA protein (fmhA)	2463
SAUSA300_2207	xanthine/uracil permease family protein	2464
SAUSA300_2395	amino acid permease	2465
SAUSA300_1247	conserved hypothetical protein	2466
SAUSA300_2412	conserved hypothetical protein	2467
SAUSA300_1522	DNA primase (dnaG) [2.7.7.-]	2468
SAUSA300_0033	methicillin-resistance MecR1 regulatory protein	2469
SAUSA300_1687-s3	FtsK/SpoIIIE family protein	2470
SAUSA300_0047	conserved hypothetical protein	2471
SAUSA300_1789	conserved hypothetical protein	2472
SAUSA300_1579	aminotransferase, class V	2473
SAUSA300_2397	putative transport protein	2474
SAUSA300_1683	chorismate mutase/phospho-2-dehydro-3-deoxyheptonate aldolase [5.4.99.5]	2475
SAUSA300_2537	L-lactate dehydrogenase [1.1.1.27]	2476
SAUSA300_1456	alpha glucosidase [3.2.1.20]	2477
SAUSA300_0836	dltB protein (dltB)	2478
SAUSA300_1137	ribonuclease HII (rnhB) [3.1.26.4]	2479
SAUSA300_0747	thioredoxin-disulfide reductase (trxB) [1.8.1.9]	2480
SAUSA300_2319	pyridine nucleotide-disulfide oxidoreductase	2481
SAUSA300_2001	similar to DNA mismatch repair protein	2482
SAUSA300_1042	DNA-dependent DNA polymerase family X	2483
SAUSA300_2501	phytoene dehydrogenase	2484
SAUSA300_1649-s2	DNA polymerase III, alpha subunit (dnaE) [2.7.7.7]	2485
SAUSA300_2516	oxidoreductase, short chain dehydrogenase/reductase family	2486
SAUSA300_0448	PTS system, trehalose-specific IIBC component (treP) [2.7.1.69]	2487
SAUSA300_0760	phosphopyruvate hydratase (eno) [4.2.1.11]	2488
SAUSA300_2442	gluconate permease (gntP)	2489
SAUSA300_0814	conserved hypothetical protein	2490
SAUSA300_0164	capsular polysaccharide biosynthesis protein Cap5M (cap5M)	2491
SAUSA300_1305	2-oxoglutarate dehydrogenase, E2 component, dihydrolipoamide succinyltransferase (sucB) [2.3.1.61]	2492
SAUSA300_0579	conserved hypothetical protein	2493
SAUSA300_1025	conserved hypothetical protein	2494
SAUSA300_2293	magnesium and cobalt transport protein (corA)	2495

SAUSA300_0704	ABC transporter, ATP-binding protein	2496
SAUSA300_1728	oxidoreductase, aldo/keto reductase family	2497
SAUSA300_0091	putative permease	2498
SAUSA300_0382	sodium:dicarboxylate symporter family protein	2499
SAUSA300_0588	conserved hypothetical protein	2500
SAUSA300_2312	malate:quinone-oxidoreductase (mqo) [1.1.99.16]	2501
SAUSA300_1972	integrase (int)	2502
SAUSA300_0903	conserved hypothetical protein	2503
SAUSA300_2288	ABC transporter, ATP-binding protein	2504
SAUSA300_2596	capsular polysaccharide biosynthesis protein Cap1C (cap1C) [3.1.3.48]	2505
SAUSA300_0198	conserved hypothetical protein	2506
SAUSA300_2265	putative amino acid permease	2507
SAUSA300_2622	conserved hypothetical protein	2508
SAUSA300_0093	transcriptional regulator, LysR family domain protein	2509
SAUSA300_1252	amino acid carrier protein	2510
SAUSA300_2487	ferrous iron transport protein B (feoB)	2511
SAUSA300_0610	putative Na+/H+ antiporter, MnhA component	2512
pUSA03_0002	IS431mec-like transposase	2513
SAUSA300_0249	2-C-methyl-D-erythritol 4-phosphate cytidylyltransferase [2.7.7.60]	2514
SAUSA300_2273	Na+/H+ antiporter family protein	2515
SAUSA300_1372	conserved hypothetical protein	2516
SAUSA300_0869	exonuclease RexB (rexB)	2517
SAUSA300_0549	glycosyl transferase, group 1 family protein [2.4.1.-]	2518
SAUSA300_2305	transposase, truncation	2519
SAUSA300_2115	IS1181, transposase (tnp)	2520
SAUSA300_1897	sodium-dependent transporter	2521
SAUSA300_2372	adenosylmethionine-8-amino-7-oxononanoate transaminase (bioA) [2.6.1.62]	2522
SAUSA300_0946	2-succinyl-6-hydroxy-2,4-cyclohexadiene-1-carboxylic acid synthase/2-oxoglutarate decarboxylase (menD) [4.1.1.71]	2523
SAUSA300_0174	conserved hypothetical protein	2524
SAUSA300_0746	TPR domain protein	2525
pUSA02_0003	replication initiation protein	2526
SAUSA300_1563	acetyl-CoA carboxylase, biotin carboxylase (accC) [6.4.1.2]	2527
SAUSA300_1557	hydrolase, HAD-superfamily, subfamily IIIA [3.1.3.-]	2528
SAUSA300_0617	Na+/H+ antiporter	2529
SAUSA300_2343	respiratory nitrate reductase, alpha subunit [1.7.99.4]	2530
SAUSA300_0952	aminotransferase, class I [2.6.1.-]	2531
SAUSA300_2304	putative membrane protein	2532
SAUSA300_1164	tRNA pseudouridine synthase B (truB) [4.2.1.70]	2533
SAUSA300_0665	acetyltransferase, GNAT family	2534
SAUSA300_0648	ABC transporter, permease protein	2535
SAUSA300_2587	accessory secretory protein Asp1	2536
SAUSA300_1170	transcriptional regulator, GntR family	2537
SAUSA300_0248	putative teichoic acid biosynthesis protein F	2538
SAUSA300_0936	ABC transporter, ATP-binding protein	2539
SAUSA300_0406	putative restriction/modification system specificity protein	2540
SAUSA300_0653	transcriptional regulator, AraC family	2541
SAUSA300_1578	tRNA (5-methylaminomethyl-2-thiouridylate)-methyltransferase (trmU) [2.1.1.61]	2542
SAUSA300_1693	conserved hypothetical protein	2543
SAUSA300_0630	ABC transporter, ATP-binding protein	2544
SAUSA300_2497	aminotransferase, class I [2.6.1.-]	2545
SAUSA300_0928	competence transcription factor (comK)	2546

SAUSA300_0086	conserved hypothetical protein	2547
SAUSA300_1062	ornithine carbamoyltransferase (argF) [2.1.3.3]	2548
SAUSA300_2334	conserved hypothetical protein	2549
SAUSA300_2044	cardiolipin synthetase (cls) [2.7.8.-]	2550
SAUSA300_1894	nicotinate phosphoribosyltransferase [2.4.2.11]	2551
SAUSA300_2575	transcriptional antiterminator, BglG family	2552
SAUSA300_0251	putative teichoic acid biosynthesis protein	2553
SAUSA300_1039	ribonuclease HIII (rnhC) [3.1.26.-]	2554
SAUSA300_0060	putative transposase	2555
SAUSA300_0473	pur operon repressor (purR)	2556
SAUSA300_1094	dihydroorotase (pyrC) [3.5.2.3]	2557
SAUSA300_2556	ABC transporter protein	2558
SAUSA300_1810	IS1181, transposase	2559
SAUSA300_0388	inosine-5-monophosphate dehydrogenase (guaB) [1.1.1.205]	2560
SAUSA300_1306	2-oxoglutarate dehydrogenase, E1 component (sucA) [1.2.4.2]	2561
SAUSA300_0566	amino acid permease	2562
SAUSA300_1189	DNA mismatch repair protein mutL (mutL)	2563
SAUSA300_0201	peptide ABC transporter, permease protein	2564
SAUSA300_0226	3-hydroxyacyl-CoA dehydrogenase	2565
SAUSA300_2240	urease, alpha subunit (ureC) [3.5.1.5]	2566
SAUSA300_0379	alkyl hydroperoxide reductase, subunit F (ahpF) [1.6.4.-]	2567
SAUSA300_1177	competence/damage-inducible protein cinA (cinA)	2568
SAUSA300_1135	putative membrane protein	2569
SAUSA300_0333	transcriptional antiterminator, BglG family	2570
SAUSA300_0503	transcriptional regulator, gntR family protein	2571
SAUSA300_0877	Chaperone clpB (clpB)	2572
SAUSA300_0670	ABC transporter, ATP-binding protein, MsbA family	2573
SAUSA300_0051	integrase, authentic frameshift	2574
SAUSA300_1929	phi77 ORF004-like protein, putative phage tail component	2575
SAUSA300_1345	asparaginyl-tRNA synthetase (asnS) [6.1.1.22]	2576
SAUSA300_1128	signal recognition particle-docking protein FtsY (ftsY)	2577
SAUSA300_0783	phosphoglycerate mutase family protein	2578
SAUSA300_0319	putative membrane protein	2579
SAUSA300_1597	holliday junction DNA helicase RuvB (ruvB)	2580
SAUSA300_1846	conserved hypothetical protein	2581
SAUSA300_0939	glycosyl transferase, group 1 family protein [2.4.1.5 2.4.1.52]	2582
SAUSA300_1701	conserved hypothetical protein	2583
SAUSA300_0914	sodium:alanine symporter family protein	2584
SAUSA300_2627	2-oxoglutarate/malate translocator	2585
SAUSA300_2109-s3	truncated FmtB protein (fmtB)	2586
SAUSA300_2227	molybdopterin biosynthesis protein B (moeB)	2587
SAUSA300_0962	quinol oxidase, subunit I (qoxB) [1.9.3.-]	2588
SAUSA300_1878	RNA methyltransferase, TrmA family (rumA) [2.1.1.-]	2589
SAUSA300_1543	oxygen-independent coproporphyrinogen III oxidase [1.3.99.22]	2590
SAUSA300_1654	proline dipeptidase [3.4.13.9]	2591
SAUSA300_1590	GTP pyrophosphokinase [2.7.6.5]	2592
SAUSA300_2393	glycine betaine/carnitine/choline ABC transporter ATP-binding protein (opuCa) [3.6.3.32]	2593
SAUSA300_1737	O-succinylbenzoate-CoA ligase (menE) [6.2.1.26]	2594
SAUSA300_2065	UDP-N-acetylglucosamine 2-epimerase [5.1.3.14]	2595
SAUSA300_0335	MATE efflux family protein	2596
SAUSA300_1752	type I restriction-modification system, M subunit (hsdM) [2.1.1.72]	2597

SAUSA300_0996	dihydrolipoamide dehydrogenase (lpdA) [1.8.1.4]	2598
SAUSA300_1667	putative glycerophosphoryl diester phosphodiesterase	2599
SAUSA300_2020	metallopeptidase, SprT family	2600
SAUSA300_0337	glycerol-3-phosphate transporter (glpT)	2601
SAUSA300_2538	amino acid permease family protein	2602
SAUSA300_0729	integral membrane protein	2603
SAUSA300_1425	conserved hypothetical phage protein	2604
SAUSA300_0210	maltose ABC transporter, permease protein	2605
SAUSA300_1525	glycyl-tRNA synthetase (glyS) [6.1.1.14]	2606
SAUSA300_1718	aresenical pump membrane protein (arsB)	2607
SAUSA300_0975	bifunctional purine biosynthesis protein (purH) [2.1.2.3 3.5.4.10]	2608
SAUSA300_0537	L-ribulokinase [2.7.1.16]	2609
SAUSA300_1610	folylpolyglutamate synthase (folC) [6.3.2.17]	2610
SAUSA300_0925	5 nucleotidase family protein	2611
SAUSA300_0955-s3	autolysin (atl) [3.5.1.28]	2612
SAUSA300_1883	high affinity proline permease (putP)	2613
SAUSA300_2000	ABC transporter, ATP-binding protein (vga)	2614
SAUSA300_0654	staphylococcal accessory protein X (sarX)	2615
pUSA03_0001	replication initiator protein (repA)	2616
SAUSA300_0702	urea amidolyase-related protein	2617
SAUSA300_1876	DNA polymerase IV	2618
SAUSA300_0981	conserved hypothetical protein	2619
SAUSA300_1651	CBS domain protein	2620
SAUSA300_0487	MesJ/Ycf62 family protein	2621
SAUSA300_1584	ATPase, AAA family	2622
SAUSA300_0034	IS1272, transposase	2623
SAUSA300_0064	arginine/oirnithine antiporter (arcD)	2624
SAUSA300_2096	mannose-6-phosphate isomerase (manA) [5.3.1.8]	2625
SAUSA300_0873	coenzyme A disulfide reductase (cdr) [1.8.1.14]	2626
SAUSA300_1454	glucose-6-phosphate 1-dehydrogenase (zwf) [1.1.1.49]	2627
SAUSA300_0339	conserved hypothetical protein	2628
SAUSA300_0405	type I restriction-modification system, M subunit (hsdM) [2.1.1.72]	2629
SAUSA300_0829	lipoic acid synthetase (lipA)	2630
SAUSA300_2014	threonine dehydratase (ilvA) [4.3.1.19]	2631
SAUSA300_0924	sodium transport family protein	2632
SAUSA300_2531	hydrolase, CocE/NonD family	2633
SAUSA300_1156	prolyl-tRNA synthetase (proS) [6.1.1.15]	2634
SAUSA300_2645	glucose-inhibited division protein A (gidA)	2635
SAUSA300_1357	chorismate synthase (aroC) [4.2.3.5]	2636
SAUSA300_2542	putative AMP-binding enzyme	2637
SAUSA300_0228	acyl-CoA synthetase FadE (fadE)	2638
SAUSA300_0845	cytosol aminopeptidase (ampA) [3.4.11.1]	2639
SAUSA300_0387	xanthine permease (pbuX)	2640
SAUSA300_1595	queuine tRNA-ribosyltransferase (tgt) [2.4.2.29]	2641
SAUSA300_0528-s3	DNA-directed RNA polymerase, beta subunit (rpoC) [2.7.7.6]	2642
SAUSA300_0945	isochorismate synthase family protein [5.4.99.6 5.4.4.2]	2643
SAUSA300_1071	conserved hypothetical protein	2644
SAUSA300_1438	phiSLT ORF401-like protein, integrase	2645
SAUSA300_1886	ATP-dependent DNA helicase, PcrA (pcrA) [3.6.1.-]	2646
SAUSA300_0755	glycolytic operon regulator	2647
SAUSA300_0328	lipoate-protein ligase A family protein [6.-.-.-]	2648
SAUSA300_1045	excinuclease ABC, C subunit (uvrC)	2649
SAUSA300_0921	peptide chain release factor 3 (prfC)	2650
SAUSA300_1423	phage related DNA polymerase, family A (polA) [2.7.7.7]	2651

SAUSA300_1858	conserved hypothetical protein	2652

Supplementary Table S3. All antigens positive for IgG reactivity in at least one sample by protein array. Ranking was determined as in Table 3, and antigens are presented in this table if the reactivity was at least two-fold above background. Ranking number, locus tag and protein name/description is shown for each antigen.

Locus Tag	Protein Name/Description	Rank
SAUSA300_0113	immunoglobulin G binding protein A (SpA)	1
SAUSA300_0398	superantigen-like protein SSL4	2
SAUSA300_0403	superantigen-like protein SSL9	3
SAUSA300_2366	gamma-hemolysin component C (hlgC)	4
SAUSA300_2579	N-acetylmuramoyl-L-alanine amidase domain protein	5
SAUSA300_0883	MAP domain-containing protein; extracellular adherence protein Eap	6
SAUSA300_2364	IgG-binding protein SBI (sbi)	7
SAUSA300_1029	Iron-regulated surface determinant Protein A (IsdA)	8
SAUSA300_1028	Iron-regulated surface determinant Protein B (IsdB)	9
SAUSA300_1920	chemotaxis-inhibiting protein CHIPS (chs)	10
SAUSA300_1382	Panton-Valentine leukocidin, LukS-PV (lukS-PV)	11
SAUSA300_2367	gamma-hemolysin component B (hlgB)	12
SAUSA300_1768	leukotoxin LukD (lukD)	13
SAUSA300_1917	map protein, programmed frameshift (map)	14
SAUSA300_0651	peptidase M23	15
SAUSA300_0951	V8 protease (sspA) [3.4.21.19]	16
SAUSA300_0408	heme uptake protein IsdB	17
SAUSA300_0438	CHAP domain family	18
SAUSA300_0409	peroxidase inhibitor	19
SAUSA300_0693	electron transfer DM13	20
SAUSA300_0214	Sugar phosphate isomerase/epimerase PFAM family PF07582	21
SAUSA300_2506	immunodominant staphylococcal antigen A precursor (isaA)	22
SAUSA300_1058	alpha-hemolysin precursor	23
SAUSA300_0548-s2	sdrE protein (sdrE)	24
SAUSA300_0776	thermonuclease precursor (nuc) [3.1.31.1]	25
SAUSA300_0547-s2	sdrD protein (sdrD)	26
SAUSA300_2572	zinc metalloproteinase aureolysin (aur) [3.4.24.29]	27
SAUSA300_0370	SEP staphylococcal enterotoxin	28
SAUSA300_1030	iron transport associated domain protein	29
SAUSA300_1476	acetyl-CoA carboxylase, biotin carboxyl carrier protein (accB)	30
SAUSA300_1922	staphylokinase precursor (sak)	31
SAUSA300_0862	glycerophosphoryl diester phosphodiesterase (glpQ) [3.1.4.46]	32
SAUSA300_1055	fibrinogen-binding protein (efb)	33
SAUSA300_1481	putative mating channel protein	34
SAUSA300_0955-s1	autolysin (atl) [3.5.1.28]	35
SAUSA300_0774	secretory extracellular matrix and plasma binding protein (empbp)	36
SAUSA300_0950	cysteine protease precursor (sspB) [3.4.22.48]	37
SAUSA300_1381	Panton-Valentine leukocidin, LukF-PV (lukF-PV)	38
SAUSA300_2441	fibronectin binding protein A (fnbA)	39
SAUSA300_2136	iron compound ABC transporter, iron compound-binding protein	40
SAUSA300_2440	fibronectin binding protein B (fnbB)	41
SAUSA300_0242	sorbitol dehydrogenase (gutB) [1.1.1.14]	42
SAUSA300_1985	serine-aspartate repeat family protein, SdrH (sdrH)	43
SAUSA300_0279	membrane protein YhgE, phage infection protein (PIP) family, type VII secretion protein EsaA	44
SAUSA300_0708	histidinol-phosphate aminotransferase (hisC) [2.6.1.9]	45
SAUSA300_0399	superantigen-like protein SSL5	46
SAUSA300_0955-s2	autolysin (atl) [3.5.1.28]	47

SAUSA300_0395	superantigen-like protein SSL1	48
SAUSA300_1031	heme ABC transporter permease	49
SAUSA300_1512	penicillin-binding protein 3 (pbp3)	50
SAUSA300_0546	sdrC protein (sdrC)	51
SAUSA300_0404	exotoxin	52
SAUSA300_0401	exotoxin	53
SAUSA300_2146	alcohol dehydrogenase, zinc-containing	54
SAUSA300_2039	D-alanine--D-alanine ligase (ddl) [6.3.2.4]	55
SAUSA300_2219	molybdenum cofactor biosynthesis protein A (moaA)	56
SAUSA300_2441-s2	fibronectin binding protein A (fnbA)	57
SAUSA300_1890	staphopain A	58
SAUSA300_0307	5-nucleotidase, lipoprotein e(P4) family	59
SAUSA300_0589	aldo/keto reductase family protein	60
SAUSA300_0602	conserved hypothetical protein	61
SAUSA300_2573	immunodominant antigen B (isaB)	62
SAUSA300_0320	triacylglycerol lipase precursor [3.1.1.3]	63
SAUSA300_2565	clumping factor B (clfB)	64
SAUSA300_1975	Aerolysin/Leukocidin family protein	65
SAUSA300_1061	putative exotoxin 3	66
SAUSA300_2441-s1	fibronectin binding protein A (fnbA)	67
SAUSA300_0573	diphosphomevalonate decarboxylase (mvaD) [4.1.1.33]	68
SAUSA300_0038	cassette chromosome recombinase A (ccrA)	69
SAUSA300_0547-s1	sdrD protein (sdrD)	70
SAUSA300_0025	5-nucleotidase family protein	71
SAUSA300_2380	conserved hypothetical protein	72
SAUSA300_1186	conserved hypothetical protein	73
SAUSA300_2476	phosphotransferase system, glucose-specific IIABC component (ptsG) [2.7.1.69]	74
SAUSA300_0396	exotoxin 7 (set7)	75
SAUSA300_1988	delta-hemolysin precursor	76
pUSA03_0034	conserved hypothetical protein	77
SAUSA300_1613	putative abrB protein	78
SAUSA300_1664	septation ring formation regulator EzrA (ezrA)	79
SAUSA300_1059	putative exotoxin 1	80
SAUSA300_2530	transcriptional regulator, TetR family	81
SAUSA300_0229	putative acyl-CoA transferase FadX	82
SAUSA300_0734	putative comf operon protein 1	83
SAUSA300_2226	molybdenum cofactor biosynthesis protein B (moaB)	84
SAUSA300_0351	putative membrane protein	85
SAUSA300_1346	putative DnaQ family exonuclease/DinG family helicase	86
SAUSA300_0441	acetyltransferase, GNAT family	87
SAUSA300_0955	autolysin (atl) [3.5.1.28]	88
SAUSA300_0830	conserved hypothetical protein	89
SAUSA300_2589	LPXTG-motif cell wall surface anchor family protein	90
SAUSA300_2285	aldose 1-epimerase (galM) [5.1.3.3]	91
SAUSA300_1895	nitric oxide synthase oxygenase	92
SAUSA300_2641	conserved hypothetical protein	93
SAUSA300_1249	conserved hypothetical protein	94
SAUSA300_0364	GTP-binding protein YchF (ychF)	95
SAUSA300_1354	conserved hypothetical protein	96
SAUSA300_1330	threonine dehydratase (ilvA) [4.3.1.19]	97
SAUSA300_0082	conserved hypothetical protein	98
SAUSA300_1879	conserved hypothetical protein	99
SAUSA300_1095	carbamoyl-phosphate synthase, small subunit (carA) [6.3.5.5]	100
SAUSA300_2101	SAP domain protein	101

SAUSA300_1514	ferric uptake regulation protein (fur)	102
SAUSA300_2322	transcriptional regulator, TetR family	103
SAUSA300_0297	putative lipoprotein	104
SAUSA300_1677	cell wall surface anchor family protein	105
SAUSA300_0397	exotoxin	106
SAUSA300_0117	iron compound ABC transporter, iron compound-binding protein SirA (sirA)	107
SAUSA300_2351	similar to Zn-binding lipoprotein adcA	108
SAUSA300_2253	secretory antigen precursor SsaA (ssaA)	109
SAUSA300_1037	phenylalanyl-tRNA synthetase, alpha subunit (pheS) [6.1.1.20]	110
SAUSA300_0224	staphylocoagulase precursor (coa)	111
SAUSA300_1961	phiPVL ORF41-like protein	112
SAUSA300_0437	NLPA lipoprotein	113
SAUSA300_1327-s1	cell surface protein	114
SAUSA300_2230	molybdenum ABC transporter, molybdenum-binding protein ModA (modA)	115
SAUSA300_0547	sdrD protein (sdrD)	116
SAUSA300_2213-s2	AcrB/AcrD/AcrF family protein	117
SAUSA300_2597	capsular polysaccharide biosynthesis protein Cap1B (cap1B) [2.7.1.112]	118
SAUSA300_0676	anion transporter family protein	119
SAUSA300_1862	conserved hypothetical protein	120
SAUSA300_1843	D-isomer specific 2-hydroxyacid dehydrogenase family protein	121
SAUSA300_0703	sulfatase family protein [3.1.6.-]	122
SAUSA300_2447	conserved hypothetical protein	123
SAUSA300_0160	capsular polysaccharide biosynthesis protein Cap5I (cap5I)	124
SAUSA300_2336	transcriptional regulator, MerR family	125
SAUSA300_1052	fibrinogen-binding protein	126
SAUSA300_1685	conserved hypothetical protein	127
SAUSA300_1140	cell wall hydrolase (lytN)	128
SAUSA300_0711	conserved hypothetical protein	129
SAUSA300_1671	conserved hypothetical protein	130
SAUSA300_1197	glutathione peroxidase	131
SAUSA300_1669	aminotransferase, class V [1.12.-.-]	132
SAUSA300_0360	Cys/Met metabolism PLP-dependent enzyme [4.4.1.8]	133
SAUSA300_1757	serine protease SplB (splB) [3.4.21.19]	134
SAUSA300_2164	conserved hypothetical protein	135
SAUSA300_0400	exotoxin	136
SAUSA300_1758	serine protease SplA (splA) [3.4.21.19]	137
SAUSA300_1053	conserved hypothetical protein	138
SAUSA300_1702-s2	cell wall surface anchor family protein	139
SAUSA300_0958	transcriptional regulator	140
SAUSA300_0964	chitinase-related protein [3.2.1.14]	141
SAUSA300_0739	LysM domain protein	142
SAUSA300_0255	sensory transduction protein LytR	143
SAUSA300_0279-s2	putative membrane protein	144
SAUSA300_2110-s1	truncated FmtB protein (fmtB)	145
SAUSA300_1753	serine protease SplF (splF) [3.4.21.19]	146
SAUSA300_0277	putative staphyloxanthin biosynthesis protein	147
SAUSA300_2055	UDP-N-acetylglucosamine 1-carboxyvinyltransferase 1 (murA) [2.5.1.7]	148
SAUSA300_1755	serine protease SplD (splD) [3.4.21.19]	149
SAUSA300_0548	sdrE protein (sdrE)	150
SAUSA300_0721	transferrin receptor	151

SAUSA300_1711	proline dehydrogenase (putA) [1.5.99.8]	152
SAUSA300_0971	phosphoribosylformylglycinamidine synthase II (purL) [6.3.5.3]	153
SAUSA300_0402	exotoxin	154
SAUSA300_2589-s1	LPXTG-motif cell wall surface anchor family protein	155
SAUSA300_1327-s16	cell surface protein	156
SAUSA300_1930-s1	phi77 ORF001-like protein, phage tail tape measure protein	157
SAUSA300_1236	conserved hypothetical protein	158
SAUSA300_1127	chromosome segregation protein SMC (smc)	159
SAUSA300_1616	uroporphyrinogen-III synthase (hemD) [4.2.1.75]	160
SAUSA300_1699	pseudouridine synthase, family 1	161
SAUSA300_1684	conserved hypothetical protein	162
SAUSA300_1974	Leukocidin/Hemolysin toxin family protein	163
SAUSA300_0987	cytochrome D ubiquinol oxidase, subunit II [1.10.3.-]	164
SAUSA300_2292	isopentenyl-diphosphate delta-isomerase (fni) [5.3.3.2]	165
SAUSA300_1091	PyrR bifunctional protein (pyrR) [2.4.2.9]	166
SAUSA300_2041	conserved hypothetical protein	167
SAUSA300_1998	putative membrane protein	168
pUSA03_0010	transfer complex protein TraA (traA)	169
SAUSA300_1407	phi SLT ORF 145-like protein, phage transcriptional regulator	170
SAUSA300_1315	PTS system, glucose-specific IIA component (crr) [2.7.1.69]	171
SAUSA300_1706	conserved hypothetical protein	172
SAUSA300_2603	triacylglycerol lipase precursor (lip) [3.1.1.3]	173
SAUSA300_2259	putative transcriptional regulator	174
SAUSA300_2473	conserved hypothetical protein	175
SAUSA300_1973	truncated beta-hemolysin	176
SAUSA300_0092	conserved hypothetical protein	177
SAUSA300_1792	conserved hypothetical protein	178
SAUSA300_1870	conserved hypothetical protein	179
SAUSA300_0157	capsular polysaccharide biosynthesis protein Cap5F (cap5F)	180
SAUSA300_2510	conserved hypothetical protein	181
SAUSA300_2276	peptidase, M20/M25/M40 family [3.5.1.14]	182
SAUSA300_0161	capsular polysaccharide biosynthesis protein Cap5J (cap5J)	183
SAUSA300_0326	conserved hypothetical protein	184
SAUSA300_1464	2-oxoisovalerate dehydrogenase, E2 component, dihydrolipoamide acetyltransferase [2.3.1.-]	185
SAUSA300_2435	cell wall surface anchor family protein	186
SAUSA300_1178	recombinase A protein (recA)	187
SAUSA300_0860	Ornithine aminotransferase (rocD) [2.6.1.13]	188
SAUSA300_1333-s2	conserved hypothetical protein	189
SAUSA300_0280	conserved hypothetical protein	190
SAUSA300_0177	conserved hypothetical protein	191
SAUSA300_2590	conserved hypothetical protein	192
SAUSA300_1588	N-acetylmuramoyl-L-alanine amidase (lytH)	193
SAUSA300_1593	protein-export membrane protein SecF (secF)	194
SAUSA300_0181-s3	non-ribosomal peptide synthetase	195
SAUSA300_1756	serine protease SplC (splC) [3.4.21.19]	196
SAUSA300_2051	conserved hypothetical protein	197
SAUSA300_0270	peptidoglycan hydrolase (lytM) [3.4.24.75]	198
SAUSA300_0279-s1	putative membrane protein	199
SAUSA300_2013	3-isopropylmalate dehydratase, small subunit (leuD) [4.2.1.33]	200
SAUSA300_2581	putative surface anchored protein	201

SAUSA300_2110	truncated FmtB protein (fmtB)	202
SAUSA300_0773	putative staphylocoagulase	203
SAUSA300_2299	multidrug resistance protein A, drug resistance transporter	204
SAUSA300_0548-s1	sdrE protein (sdrE)	205
SAUSA300_0489	putative cell division protein FtsH	206
SAUSA300_1341	penicillin binding protein 2 (pbp2) [2.4.2.-]	207
SAUSA300_1097	orotidine 5-phosphate decarboxylase (pyrF) [4.1.1.23]	208
SAUSA300_0099	1-phosphatidylinositol phosphodiesterase (plc) [3.1.4.10]	209
SAUSA300_0886	3-oxoacyl-(acyl-carrier-protein) synthase II (fabF) [2.3.1.41]	210
SAUSA300_0383	conserved hypothetical protein	211
SAUSA300_2463	D-lactate dehydrogenase (ddh) [1.1.1.28]	212
SAUSA300_0841	conserved hypothetical protein	213
SAUSA300_2321	putative membrane protein	214
SAUSA300_2500	glycosyl transferase	215
SAUSA300_0419	staphylococcus tandem lipoprotein	216
SAUSA300_2161	Hyaluronate lyase precursor (hysA) [4.2.2.1]	217
SAUSA300_1863	conserved hypothetical protein	218
SAUSA300_0769	putative lipoprotein	219
SAUSA300_1606	conserved hypothetical protein	220
SAUSA300_0995	dihydrolipoamide acetyltransferase	221
SAUSA300_1428	conserved hypothetical phage protein	222
SAUSA300_2457	phospholipase/carboxylesterase family protein	223
SAUSA300_1299	putative tellurite resistance protein	224
SAUSA300_2166	alpha-acetolactate synthase (alsS) [2.2.1.6]	225
SAUSA300_2643	putative chromosome partioning protein, ParB family	226
SAUSA300_2109-s2	truncated FmtB protein (fmtB)	227
SAUSA300_0798	ABC transporter, substrate-binding protein	228
SAUSA300_0965	methylenetetrahydrofolate dehydrogenase/methenyltetrahydrofolate cyclohydrolase (folD) [1.5.1.5]	229
SAUSA300_0627	teichoic acid biosynthesis protein X (tagX)	230
SAUSA300_1905	conserved hypothetical protein	231
SAUSA300_1060	putative exotoxin 4	232
SAUSA300_1361	conserved hypothetical protein	233
SAUSA300_2505	acetyltransferase, GNAT family [2.3.1.-]	234
SAUSA300_1179	conserved hypothetical protein	235
SAUSA300_0913	putative membrane protein	236
SAUSA300_1023	conserved hypothetical protein	237
SAUSA300_1707	conserved hypothetical protein	238
SAUSA300_0984	phosphoenolpyruvate-protein phosphotransferase (ptsI) [2.7.3.9]	239
SAUSA300_0247	putative teichoic acid biosynthesis protein B	240
SAUSA300_0764	ribonuclease R (rnr) [3.1.-.-]	241
SAUSA300_0943	acetyltransferase, GNAT family family	242
SAUSA300_1648	putative NADP-dependent malic enzyme	243
SAUSA300_0450	trehalose operon repressor (treR)	244
SAUSA300_0022	YycH protein	245
SAUSA300_0443	YibE/F-like protein	246
SAUSA300_1652	conserved hypothetical protein	247
SAUSA300_1291	hippurate hydrolase [3.5.1.14]	248
SAUSA300_0469	primase-related protein	249
SAUSA300_1465	2-oxoisovalerate dehydrogenase, E1 component, beta subunit [1.2.4.1]	250
SAUSA300_1617	porphobilinogen deaminase (hemC) [2.5.1.61]	251

SAUSA300_0815	Ear protein (ear)	252
SAUSA300_2110-s2	truncated FmtB protein (fmtB)	253
SAUSA300_1289	dihydrodipicolinate reductase (dapB) [1.3.1.26]	254
SAUSA300_2436	putative cell wall surface anchor family protein	255
SAUSA300_0424.2	psmA3	256
SAUSA300_1884	CamS sex pheromone cAM373 precursor	257
SAUSA300_1532	conserved hypothetical protein	258
SAUSA300_1046	succsuccinate dehydrogenase, cytochrome b-558 subunit (sdhC)	259
SAUSA300_2109-s1	truncated FmtB protein (fmtB)	260
SAUSA300_2462	NAD(P)H-flavin oxidoreductase (frp) [1.6.99.-]	261
SAUSA300_1978	ferric hydroxamate receptor	262
SAUSA300_2235	iron compound ABC transporter, iron compound-binding protein	263
SAUSA300_1096-s2	carbamoyl-phosphate synthase, large subunit (carB) [6.3.5.5]	264
SAUSA300_0200	peptide ABC transporter, ATP-binding protein	265
SAUSA300_0213	oxidoreductase, Gfo/Idh/MocA family	266
SAUSA300_1702-s1	cell wall surface anchor family protein	267
SAUSA300_1043	DNA mismatch repair MutS2 protein (mutS2)	268
SAUSA300_2646	tRNA modification GTPase (trmE)	269
SAUSA300_0359	trans-sulfuration enzyme family protein	270
SAUSA300_1122	fatty acid/phospholipid synthesis protein PlsX (plsX)	271
SAUSA300_2307	ABC transporter, permease protein	272
SAUSA300_2172	50S ribosomal protein L13 (rplM)	273
SAUSA300_0775	conserved hypothetical protein	274
SAUSA300_0992	putative lipoprotein	275
SAUSA300_1243-s1	exonuclease SbcC (sbcC)	276
SAUSA300_1533	conserved hypothetical protein	277
SAUSA300_0223	conserved hypothetical protein	278
SAUSA300_2316	acetyltransferase, GNAT family	279
SAUSA300_1639	alkaline phosphatase synthesis transcriptional regulatory protein PhoP (phoP)	280
SAUSA300_2087	putative peptidase	281
SAUSA300_0527-s2	DNA-directed RNA polymerase, beta subunit (rpoB) [2.7.7.6]	282
SAUSA300_1889	adenylosuccinate lyase (purB) [4.3.2.2]	283
SAUSA300_1769	leukotoxin LukE (lukE)	284
SAUSA300_0136	cell wall surface anchor family protein	285
SAUSA300_2540	fructose-bisphosphate aldolase class-I [4.1.2.13]	286
SAUSA300_1143	DNA topoisomerase I (topA) [5.99.1.2]	287
SAUSA300_1942	conserved hypothetical phage protein	288
SAUSA300_1919	conserved hypothetical protein	289
SAUSA300_0724	putative lipoprotein	290
SAUSA300_1014-s1	pyruvate carboxylase (pyc) [6.4.1.1]	291
SAUSA300_0982	conserved hypothetical protein	292
SAUSA300_0414	staphylococcus tandem lipoprotein	293
SAUSA300_1759	conserved hypothetical protein	294
SAUSA300_1449	MutT/nudix family protein [3.6.1.13]	295
SAUSA300_1622	trigger factor (tig) [5.2.1.8]	296
SAUSA300_1564	acetyl-CoA carboxylase, biotin carboxyl carrier protein (accB)	297
SAUSA300_1687-s2	FtsK/SpoIIIE family protein	298
SAUSA300_0463	conserved hypothetical protein	299
SAUSA300_2371	biotin synthase (bioB) [2.8.1.6]	300
SAUSA300_0244	oxidoreductase, zinc-binding dehydrogenase family	301

SAUSA300_1486	conserved hypothetical protein	302
SAUSA300_1446	conserved hypothetical protein	303
SAUSA300_2068	conserved hypothetical protein	304
SAUSA300_0166	capsular polysaccharide biosynthesis protein Cap5O (cap5O) [1.1.1.233]	305
SAUSA300_1928-s2	phi77 ORF002-like protein, phage minor structural protein	306
SAUSA300_0599	iron compound ABC transporter, permease protein	307
SAUSA300_0535	putative pyridoxal phosphate-dependent acyltransferase	308
SAUSA300_2278	urocanate hydratase (hutU) [4.2.1.49]	309
SAUSA300_1113	protein kinase (pknB) [2.7.1.-]	310
SAUSA300_2589-s2	LPXTG-motif cell wall surface anchor family protein	311
SAUSA300_2578	putative phage infection protein	312
SAUSA300_2561	alkaline phosphatase (phoB) [3.1.3.1]	313
SAUSA300_0308	ABC transporter, permease protein	314
SAUSA300_1729	conserved hypothetical protein	315
SAUSA300_0411	staphylococcus tandem lipoprotein	316
SAUSA300_1175	conserved hypothetical protein	317
SAUSA300_0685	fructose specific permease (fruA)	318
SAUSA300_1155	putative membrane-associated zinc metalloprotease	319
SAUSA300_1130	signal recognition particle protein (ffh)	320
SAUSA300_1644	pyruvate kinase (pyk) [2.7.1.40]	321
SAUSA300_0800	staphylococcal enterotoxin K (sek)	322
SAUSA300_2249	secretory antigen precursor SsaA (ssaA)	323
SAUSA300_1075	penicillin-binding protein 1 (pbpA)	324
SAUSA300_1162	translation initiation factor IF-2 (infB)	325
SAUSA300_0879	isopropylmalate synthase-related protein	326
SAUSA300_0006	DNA gyrase, A subunit (gyrA) [5.99.1.3]	327
SAUSA300_1702-s3	cell wall surface anchor family protein	328
SAUSA300_1599	ACT domain protein PheB	329
SAUSA300_1157-s3	DNA polymerase III, alpha subunit (polC) [2.7.7.7]	330
SAUSA300_1401	phiSLT ORF387-like protein, putative phage capsid protein	331
SAUSA300_0505	conserved hypothetical protein	332
SAUSA300_0889	oligopeptide ABC transporter, ATP-binding protein (oppD)	333
SAUSA300_0580	conserved hypothetical protein	334
SAUSA300_1367	cytidylate kinase (cmk) [2.7.4.14]	335
SAUSA300_0870-s2	exonuclease RexA (rexA)	336
SAUSA300_1697	Peptidase family M20/M25/M40 [3.4.-.-]	337
SAUSA300_2213-s1	AcrB/AcrD/AcrF family protein	338
SAUSA300_0264	ribose transporter RbsU	339
SAUSA300_0677	putative deoxyribodipyrimidine photolyase	340
SAUSA300_1149	30S ribosomal protein S2 (rpsB)	341
SAUSA300_1468	DNA repair protein RecN (recN)	342
SAUSA300_0509	ATP guanido phosphotransferase	343
SAUSA300_1713	riboflavin biosynthesis protein (ribBA) [3.5.4.25]	344
SAUSA300_0296	conserved hypothetical protein	345
SAUSA300_1559	putative enterotoxin type A	346
SAUSA300_0560	acetyl-CoA c-acetyltransferase (vraB) [2.3.1.9]	347
SAUSA300_0283-s3	essC protein	348
SAUSA300_1521	RNA polymerase sigma factor RpoD (rpoD)	349
SAUSA300_1754	serine protease SplE (splE) [3.4.21.19]	350
SAUSA300_0680	multi drug resistance protein (norA)	351
SAUSA300_1012	conserved hypothetical protein	352
SAUSA300_0167	capsular polysaccharide biosynthesis protein Cap5P (cap5P) [5.1.3.14]	353
SAUSA300_1938	phi77 ORF006-like protein, putative capsid protein	354
SAUSA300_0344	putative lipoprotein	355

SAUSA300_0283-s2	essC protein	356
SAUSA300_2254	similar to glycerate dehydrogenase	357
SAUSA300_1796	conserved hypothetical protein	358
SAUSA300_0418	staphylococcus tandem lipoprotein	359
SAUSA300_2213	AcrB/AcrD/AcrF family protein	360
SAUSA300_0105	peptidase, M20/M25/M40 family [3.5.1.14]	361
SAUSA300_1855	monofunctional glycosyltransferase (sgtB) [3.4.-.-]	362
SAUSA300_1674	putative serine protease HtrA	363
SAUSA300_1272	conserved hypothetical protein	364
SAUSA300_1257	peptide methionine sulfoxide reductase regulator MsrR (msrR)	365
SAUSA300_1285	ABC transporter, ATP-binding protein	366
SAUSA300_0415	staphylococcus tandem lipoprotein (lpl3)	367
SAUSA300_2141	putative transposase, degenerate	368
SAUSA300_1286	aspartate kinase [2.7.2.4]	369
SAUSA300_1741	putative lipoprotein	370
SAUSA300_0348	twin-arginine translocation protein, TatA/E family	371
SAUSA300_0203	putative lipoprotein	372
SAUSA300_0274	conserved hypothetical protein	373
SAUSA300_2503	secretory antigen precursor SsaA	374
SAUSA300_0065	arginine deiminase (arcA) [3.5.3.6]	375
SAUSA300_1893	NH(3)-dependent NAD+ synthetase (nadE) [6.3.1.5]	376
SAUSA300_1605	rod shape-determining protein MreC (mreC)	377
SAUSA300_1682	catabolite control protein A (ccpA)	378
SAUSA300_1344	putative DNA replication protein DnaD	379
SAUSA300_1864	putative membrane protein	380
SAUSA300_1475	acetyl-CoA carboxylase, biotin carboxylase (accC) [6.4.1.2]	381
SAUSA300_1138	succinyl-CoA synthetase, beta subunit (sucC) [6.2.1.5]	382
SAUSA300_2302	teicoplanin resistance associated membrane protein TcaA protein (tcaA)	383
SAUSA300_1347	BirA bifunctional protein (birA) [6.3.4.15]	384
SAUSA300_2023	anti-sigma-B factor, serine-protein kinase (rsbW) [2.7.1.37]	385
SAUSA300_2148	conserved hypothetical protein	386
SAUSA300_0864	argininosuccinate synthase (argG) [6.3.4.5]	387
SAUSA300_1127-s2	chromosome segregation protein SMC (smc)	388
SAUSA300_1078	cell division protein (divIB)	389
SAUSA300_0866	conserved hypothetical protein	390
SAUSA300_1480	putative traG membrane protein	391
pUSA03_0016	transfer complex protein TraG (traG)	392
SAUSA300_1127-s1	chromosome segregation protein SMC (smc)	393
pUSA03_0027	conserved hypothetical protein	394
SAUSA300_1243	exonuclease SbcC (sbcC)	395
SAUSA300_1702-s4	cell wall surface anchor family protein	396
SAUSA300_0861	NAD-specific glutamate dehydrogenase (gudB) [1.4.1.2]	397
SAUSA300_0870-s1	exonuclease RexA (rexA)	398
SAUSA300_1350	conserved hypothetical protein	399
SAUSA300_0716	ribonucleoside-diphosphate reductase, alpha subunit [1.17.4.1]	400
SAUSA300_1680	acetoin utilization protein AcuA (acuA) [2.3.1.-]	401
SAUSA300_0905	putative adenylate cyclase	402
SAUSA300_0793	conserved hypothetical protein	403
SAUSA300_1903	conserved hypothetical protein	404
SAUSA300_1503	putative competence protein ComGB	405
SAUSA300_1217	ABC transporter, ATP-binding protein	406
SAUSA300_0032	penicillin-binding protein 2 (mecA) [3.4.16.4]	407

SAUSA300_0893	oligopeptide ABC transporter, ATP-binding protein (oppF)	408
pUSA03_0033	conserved hypothetical protein	409
SAUSA300_0947	hydrolase, alpha/beta hydrolase fold family [3.4.11.5]	410
SAUSA300_0258	transcriptional regulator, GntR family	411
SAUSA300_2332	heat shock protein	412
SAUSA300_1124	3-oxoacyl-(acyl-carrier-protein) reductase (fabG) [1.1.1.100]	413
SAUSA300_2076	aldehyde dehydrogenase family protein	414
SAUSA300_1940	phage portal protein	415
SAUSA300_2114	arginase (rocF) [3.5.3.1]	416
SAUSA300_0801	staphylococcal enterotoxin Q (seq)	417
SAUSA300_1495	conserved hypothetical protein	418
SAUSA300_1119	conserved hypothetical protein	419
SAUSA300_1172	peptidase, M16 family [3.4.24.56]	420
SAUSA300_0028	putative transposase	421
SAUSA300_2574	conserved hypothetical protein	422
SAUSA300_2315	putative lipoprotein	423
SAUSA300_1327-s2	cell surface protein	424
SAUSA300_1574	conserved hypothetical protein	425
SAUSA300_1393-s3	phiSLT ORF2067-like protein, phage tail tape measure protein	426
SAUSA300_1415	phiSLT ORF 77-like protein	427
SAUSA300_2153	tagatose-6-phosphate kinase (lacC) [2.7.1.144]	428
SAUSA300_0336	conserved hypothetical protein	429
SAUSA300_2504	acyltransferase	430
pUSA03_0031	LtrC-like protein	431
SAUSA300_2623	pyrrolidone-carboxylate peptidase (pcp) [3.4.19.3]	432
SAUSA300_0141	phosphopentomutase (deoB) [5.4.2.7]	433
SAUSA300_1290	tetrahydrodipicolinate acetyltransferase (dapD) [2.3.1.117]	434
SAUSA300_0237	inosine-uridine preferring nucleoside hydrolase	435
SAUSA300_2427	conserved hypothetical protein, authentic frameshift	436
SAUSA300_0023	YycI protein	437
SAUSA300_1187	conserved hypothetical protein	438
SAUSA300_1396	phiSLT ORF151-like protein, major tail protein	439
SAUSA300_2486	putative ATP-dependent Clp proteinase	440
SAUSA300_1026	conserved hypothetical protein	441
SAUSA300_0552	conserved hypothetical protein	442
SAUSA300_0413	staphylococcus tandem lipoprotein	443
SAUSA300_1158	conserved hypothetical protein	444
SAUSA300_0003	conserved hypothetical protein	445
SAUSA300_0759	2,3-bisphosphoglycerate-independent phosphoglycerate mutase (gpmI) [5.4.2.1]	446
SAUSA300_1413	conserved hypothetical phage protein	447
SAUSA300_2178	DNA-directed RNA polymerase alpha subunit (rpoA) [2.7.7.6]	448
SAUSA300_0170	aldehyde dehydrogenase [1.2.1.3]	449
SAUSA300_0634	ferrichrome transport permease protein fhuB (fhuB)	450
SAUSA300_1725	transaldolase [2.2.1.2]	451
SAUSA300_2566	transcriptional regulator, Crp/Fnr family (arcR)	452
SAUSA300_0477	UDP-N-acetylglucosamine pyrophosphorylase (glmU) [2.7.7.23]	453
SAUSA300_0885	3-oxoacyl-(acyl-carrier-protein) synthase III (fabH) [2.3.1.41]	454
SAUSA300_2554	sulfite reductase flavoprotein [1.8.1.2]	455
SAUSA300_0562	phosphomethylpyrimidine kinase (thiD) [2.7.4.7]	456

SAUSA300_1048	succinate dehydrogenase, iron-sulfur protein (sdhB) [1.3.99.1]	457
SAUSA300_0821	SUF system FeS assembly protein, NifU family	458
SAUSA300_0846	Na+/H+ antiporter family protein	459
SAUSA300_0235	L-lactate dehydrogenase [1.1.1.27]	460
SAUSA300_0993	pyruvate dehydrogenase E1 component, alpha subunit (pdhA) [1.2.4.1]	461
SAUSA300_0869-s2	exonuclease RexB (rexB)	462
SAUSA300_1540	chaperone protein DnaK (dnaK)	463
SAUSA300_2387	NAD dependent epimerase/dehydratase family protein	464
SAUSA300_1157-s1	DNA polymerase III, alpha subunit (polC) [2.7.7.7]	465
SAUSA300_2089	pyrimidine nucleoside phosphorylase (pdp) [2.4.2.2]	466
SAUSA300_0262	ribokinase (rbsK) [2.7.1.15]	467
SAUSA300_2100	lytic regulatory protein	468
SAUSA300_1808	amino acid ABC transporter, permease/substrate-binding protein	469
SAUSA300_2297	conserved hypothetical protein	470
SAUSA300_1976	probable succinyl-diaminopimelate desuccinylase	471
SAUSA300_1611	valyl-tRNA synthetase (valS) [6.1.1.9]	472
SAUSA300_2455	putative fructose-1,6-bisphosphatase	473
SAUSA300_2112	conserved hypothetical protein	474
SAUSA300_0031	conserved hypothetical protein	475
SAUSA300_1696	D-alanine aminotransferase (dat) [2.6.1.21]	476
SAUSA300_0743	HPr(Ser) kinase/phosphatase (hprK) [2.7.1.-]	477
SAUSA300_2341	respiratory nitrate reductase, delta subunit (narJ)	478
SAUSA300_2634	ABC transporter, permease protein	479
SAUSA300_1770	conserved hypothetical protein	480
SAUSA300_0547-s3	sdrD protein (sdrD)	481
SAUSA300_0021	sensory box histidine kinase [2.7.3.-]	482
SAUSA300_2420	conserved hypothetical protein	483
SAUSA300_2478	Holin-like protein cidB (cidB)	484
SAUSA300_2086	conserved hypothetical protein	485
SAUSA300_1418	phiSLT ORF 82-like protein	486
SAUSA300_2498	squalene synthase (crtN) [1.3.-.-]	487
SAUSA300_2458	glyoxylase family protein	488
SAUSA300_1575	alanyl-tRNA synthetase (alaS) [6.1.1.7]	489
SAUSA300_2374	ABC transporter, ATP-binding/permease protein	490
SAUSA300_1845	glutamate-1-semialdehyde-2,1-aminomutase (hemL) [5.4.3.8]	491
SAUSA300_2354	putative lipoprotein	492
SAUSA300_1624	MutT/nudix family protein	493
SAUSA300_2377	glycerate kinase [2.7.1.31]	494
SAUSA300_1150	translation elongation factor Ts (tsf)	495
SAUSA300_0377	putative lipoprotein	496
SAUSA300_1888	conserved hypothetical protein	497
SAUSA300_0372	putative lipoprotein	498
SAUSA300_0485	cell-division initiation protein	499
SAUSA300_1002	spermidine/putrescine ABC transporter, spermidine/putrescine-binding protein (potD)	500
SAUSA300_1007	inositol monophosphatase family protein [3.1.3.25]	501
SAUSA300_1087	isoleucyl-tRNA synthetase (ileS) [6.1.1.5]	502
SAUSA300_1034	sortase B (srtB)	503
SAUSA300_0869-s1	exonuclease RexB (rexB)	504
SAUSA300_1089	lipoprotein signal peptidase (lspA) [3.4.23.36]	505
SAUSA300_2358	ABC transporter, permease protein	506
SAUSA300_0763	carboxylesterase (est) [3.1.1.1]	507

SAUSA300_0445-s2	glutamate synthase, large subunit (gltB) [1.4.1.13]	508
SAUSA300_2400	glutamyl-aminopeptidase	509
SAUSA300_2424	putative staphylococcus tandem lipoprotein	510
SAUSA300_1219	putative sensor histidine kinase	511
pUSA01_0004	hypothetical protein	512
SAUSA300_0570	phosphate acetyltransferase (pta) [2.3.1.8]	513
SAUSA300_2060	ATP synthase F1, alpha subunit (atpA) [3.6.3.14]	514
SAUSA300_0642	conserved hypothetical protein	515
SAUSA300_1106	putative lipoprotein	516
SAUSA300_1270	methicillin resistance protein FemB (femB)	517
SAUSA300_2484	hydroxymethylglutaryl-CoA synthase [2.3.3.10]	518
SAUSA300_0230	putative membrane protein	519
SAUSA300_1398	phiSLT ORF123-like protein	520
SAUSA300_1960	putative phage-related DNA recombination protein	521
SAUSA300_0645	DNA-binding response regulator	522
SAUSA300_0600	hydrolase, haloacid dehalogenase-like family	523
SAUSA300_0536	DJ-1/PfpI family protein	524
SAUSA300_0853	Na(+)/H(+) antiporter subunit C (mnhC)	525
SAUSA300_0030	putative glycerophosphoryl diester phosphodiesterase	526
SAUSA300_2370	putative 8-amino-7-oxononanoate synthase	527
SAUSA300_0754	conserved hypothetical protein	528
SAUSA300_2343-s1	respiratory nitrate reductase, alpha subunit [1.7.99.4]	529
SAUSA300_1378	conserved hypothetical protein	530
SAUSA300_2248	transcriptional regulator, AraC family	531
SAUSA300_0212	oxidoreductase, Gfo/Idh/MocA family	532
SAUSA300_0999	spermidine/putrescine ABC transporter, ATP-binding protein (potA)	533
SAUSA300_1881	Aspartyl/glutamyl-tRNA amidotransferase subunit A (gatA) [6.3.5.-]	534
SAUSA300_2301	teicoplanin resistance associated membrane protein TcaB protein (tcaB)	535
SAUSA300_2109	truncated FmtB protein (fmtB)	536
SAUSA300_2069	conserved hypothetical protein	537
SAUSA300_0633	ferrichrome transport ATP-binding protein fhuA (fhuA)	538
SAUSA300_1426	conserved hypothetical phage protein	539
SAUSA300_1631	replication initiation and membrane attachment protein	540
SAUSA300_2411	oligopeptide permease, peptide-binding protein (opp-1A)	541
SAUSA300_1047	succinate dehydrogenase, flavoprotein subunit (sdhA) [1.3.99.1]	542
SAUSA300_0424	putative cobalamin synthesis protein	543
SAUSA300_2429	staphylococcus tandem lipoprotein	544
SAUSA300_0181-s4	non-ribosomal peptide synthetase	545
SAUSA300_1911	ABC transporter, ATP-binding protein	546
SAUSA300_0888	oligopeptide ABC transporter, permease protein (oppC)	547
SAUSA300_2520	transporter gate domain protein	548
SAUSA300_1641	citrate synthase II (gltA) [4.1.3.7]	549
SAUSA300_2244	urease accessory protein UreD (ureD) [3.5.1.5]	550
SAUSA300_2035	sensor histidine kinase, KdpD (kdpD) [2.7.3.-]	551
SAUSA300_1640	isocitrate dehydrogenase, NADP-dependent (icd) [1.1.1.42]	552
SAUSA300_1781	protoporphyrinogen oxidase (hemG) [1.3.3.4]	553
SAUSA300_0492	dihydropteroate synthase (folP) [2.5.1.15]	554
SAUSA300_1678	formate-tetrahydrofolate ligase (fhs) [6.3.4.3]	555
SAUSA300_1483	conserved hypothetical protein	556
SAUSA300_0892	oligopeptide ABC transporter, oligopeptide-binding protein (oppA)	557

SAUSA300_1147	heat shock protein HslVU, ATPase subunit HslU (hslU)	558
SAUSA300_1098	orotate phosphoribosyltransferase (pyrE) [2.4.2.10]	559
SAUSA300_1111	conserved hypothetical protein	560
SAUSA300_0935	conserved hypothetical protein	561
SAUSA300_2598	capsular polysaccharide biosynthesis protein Cap1A (cap1A)	562
SAUSA300_1246	aconitate hydratase (acnA) [4.2.1.3]	563
SAUSA300_1704	leucyl-tRNA synthetase (leuS) [6.1.1.4]	564
SAUSA300_1705	putative drug transporter	565
SAUSA300_1301	conserved hypothetical protein	566
SAUSA300_0179	putative D-isomer specific 2-hydroxyacid dehydrogenase	567
SAUSA300_2329	proton/sodium-glutamate symport protein (gltT)	568
SAUSA300_1327-s15	cell surface protein	569
SAUSA300_1227	threonine synthase (thrC) [4.2.3.1]	570
SAUSA300_1171	conserved hypothetical protein	571
SAUSA300_1307	sensor histidine kinase protein (arlS) [2.7.3.-]	572
SAUSA300_0527-s1	DNA-directed RNA polymerase, beta subunit (rpoB) [2.7.7.6]	573
SAUSA300_0758	triosephosphate isomerase (tpiA) [5.3.1.1]	574
SAUSA300_0844	conserved hypothetical protein	575
SAUSA300_0871	conserved hypothetical protein	576
SAUSA300_2483	hydroxymethylglutaryl-CoA reductase [1.1.1.88]	577
SAUSA300_0002	DNA polymerase III, beta subunit (dnaN) [2.7.7.7]	578
SAUSA300_2419	conserved hypothetical protein	579
SAUSA300_1014	pyruvate carboxylase (pyc) [6.4.1.1]	580
SAUSA300_0475	SpoVG protein	581
SAUSA300_2149	6-phospho-beta-galactosidase (lacG) [3.2.1.85]	582
SAUSA300_1891	conserved hypothetical protein	583
SAUSA300_1698	conserved hypothetical protein	584
SAUSA300_0375	putative phosphoglycerate mutase family protein	585
SAUSA300_2470	L-serine dehydratase, iron-sulfur-dependent, beta subunit (sdaAB) [4.3.1.17]	586
SAUSA300_2125	ATP-binding protein, Mrp/Nbp35 family	587
SAUSA300_0462	conserved hypothetical protein	588
SAUSA300_1689	conserved hypothetical protein	589
SAUSA300_1168	RNA-metabolising metallo-beta-lactamase	590
SAUSA300_1011	conserved hypothetical protein	591
SAUSA300_1494	conserved hypothetical protein	592
SAUSA300_1807	amino acid ABC transporter, ATP-binding protein	593
SAUSA300_1090	conserved hypothetical protein	594
SAUSA300_1459	6-phosphogluconate dehydrogenase, decarboxylating (gnd) [1.1.1.44]	595
pUSA03_0022	transfer complex protein TraM (traM)	596
SAUSA300_2471	perfringolysin O regulator protein	597
SAUSA300_0192	conserved hypothetical protein	598
SAUSA300_1169	DNA translocase FtsK (ftsK)	599
SAUSA300_1245	glycine betaine transporter opuD (opuD)	600
SAUSA300_2113	conserved hypothetical protein	601
SAUSA300_1712	riboflavin synthase, beta subunit (ribH) [6.3.3.-]	602
SAUSA300_1600	GTP-binding protein Obg/CgtA	603
SAUSA300_2398	putative membrane protein	604
SAUSA300_1153	undecaprenyl diphosphate synthase (uppS) [2.5.1.31]	605
SAUSA300_0204	gamma-glutamyltranspeptidase (ggt) [2.3.2.2]	606
SAUSA300_0934	membrane protein	607
SAUSA300_1766	lantibiotic epidermin biosynthesis protein EpiB (epiB)	608
SAUSA300_1199	putative aluminium resistance protein	609

SAUSA300_2208	DNA topoisomerase III (topB) [5.99.1.2]	610
SAUSA300_1467	2-oxoisovalerate dehydrogenase, E3 component, lipoamide dehydrogenase (lpdA) [1.8.1.4]	611
SAUSA300_0194	sucrose-specific PTS tranporter protein	612
SAUSA300_0722	UDP-N-acetylenolpyruvoylglucosamine reductase (murB) [1.1.1.158]	613
SAUSA300_0959	fmt protein (fmt) [3.5.2.6]	614
SAUSA300_1539	chaperone protein DnaJ (dnaJ)	615
SAUSA300_1860	aminopeptidase PepS (pepS) [3.4.11.-]	616
SAUSA300_1327-s3	cell surface protein	617
SAUSA300_1327-s14	cell surface protein	618
SAUSA300_1327-s7	cell surface protein	619
SAUSA300_1327-s9	cell surface protein	620
SAUSA300_0733	degV family protein	621
SAUSA300_0686	N-acetylglucosamine-6-phosphate deacetylase (nagA) [3.5.1.25]	622
SAUSA300_1327-s4	cell surface protein	623
SAUSA300_1067	antibacterial protein	624
SAUSA300_1411	phiSLT ORF66-like protein	625
SAUSA300_1082	conserved hypothetical protein	626
SAUSA300_1619	glutamyl-tRNA reductase (hemA) [1.2.1.-]	627
SAUSA300_1327-s12	cell surface protein	628
SAUSA300_2449	putative transporter	629
SAUSA300_1327-s8	cell surface protein	630
SAUSA300_0416	staphylococcus tandem lipoprotein	631
SAUSA300_1390	phiSLT ORF96-like protein	632
SAUSA300_0528-s2	DNA-directed RNA polymerase, beta subunit (rpoC) [2.7.7.6]	633
SAUSA300_1794	conserved hypothetical protein	634
SAUSA300_2078	UDP-N-acetylglucosamine 1-carboxyvinyltransferase (murA) [2.5.1.7]	635
SAUSA300_1327-s13	cell surface protein	636
SAUSA300_1327-s6	cell surface protein	637
SAUSA300_1928-s1	phi77 ORF002-like protein, phage minor structural protein	638
SAUSA300_0452	DNA polymerase III, gamma and tau subunits (dnaX) [2.7.7.7]	639
SAUSA300_0948	naphthoate synthase (menB) [4.1.3.36]	640
SAUSA300_0205	staphylococcus tandem lipoprotein	641
SAUSA300_0963	quinol oxidase, subunit II (qoxA) [1.9.3.-]	642
SAUSA300_2343-s2	respiratory nitrate reductase, alpha subunit [1.7.99.4]	643
SAUSA300_1327-s5	cell surface protein	644
pUSA03_0014	transfer complex protein TraE (traE)	645
SAUSA300_1327-s10	cell surface protein	646
SAUSA300_1842	transcriptional regulator, Fur family	647
SAUSA300_2475	conserved hypothetical protein	648
SAUSA300_0690	sensor histidine kinase SaeS (saeS) [2.7.3.-]	649
SAUSA300_0417	staphylococcus tandem lipoprotein	650
SAUSA300_1077	UDP-N-acetylmuramoylalanine--D-glutamate ligase (murD) [6.3.2.9]	651
SAUSA300_0973	phosphoribosylformylglycinamidine cyclo-ligase (purM) [6.3.3.1]	652
SAUSA300_0511	DNA repair protein RadA (radA)	653
SAUSA300_2105	PTS system, mannitol specific IIBC component (mtlF) [2.7.1.69]	654
SAUSA300_1327-s11	cell surface protein	655
SAUSA300_2589-s3	LPXTG-motif cell wall surface anchor family protein	656

SAUSA300_2550	anaerobic ribonucleotide reductase, small subunit (nrdG) [1.97.1.4]	657
SAUSA300_1006	conserved hypothetical protein	658
SAUSA300_0152	capsular polysaccharide biosynthesis protein Cap5A (cap5A)	659
SAUSA300_1866	two-component sensor histidine kinase (vraS) [2.7.3.-]	660
SAUSA300_2224	molybdopterin biosynthesis protein A (moeA)	661
SAUSA300_2003	ribosomal-protein-alanine acetyltransferase (rimI) [2.3.1.128]	662
SAUSA300_2567	carbamate kinase (arcC) [2.7.2.2]	663
SAUSA300_1385	phiSLT ORF 99-like protein	664
SAUSA300_0707	osmoprotectant ABC transporter, permease [3.6.3.32]	665
SAUSA300_1910	putative membrane protein	666
SAUSA300_0445-s1	glutamate synthase, large subunit (gltB) [1.4.1.13]	667
SAUSA300_1139	succinyl-CoA synthetase, alpha subunit (sucD) [6.2.1.5]	668
SAUSA300_0970	phosphoribosylformylglycinamidine synthase I (purQ) [6.3.5.3]	669
SAUSA300_0056	conserved hypothetical protein	670
SAUSA300_1739	conserved hypothetical protein	671
SAUSA300_1376	putative lipoprotein	672
SAUSA300_1181	conserved hypothetical protein	673
SAUSA300_1569	peptidase, U32 family [3.4.-.-]	674
SAUSA300_0479	ribosomal protein L25, Ctc-form	675
SAUSA300_0449	alpha,alpha-phosphotrehalase (treC) [3.2.1.93]	676
SAUSA300_0858	conserved hypothetical protein	677
SAUSA300_2214	FmhB protein	678
SAUSA300_0424.1	psmA4	679
SAUSA300_0771	acetyltransferase, GNAT family [2.3.1.-]	680
SAUSA300_0717	ribonucleoside-diphosphate reductase, beta subunit [1.17.4.1]	681
SAUSA300_0045	HNH endonuclease family protein	682
SAUSA300_2477	pyruvate oxidase (cidC) [1.2.3.3]	683
SAUSA300_1443	ribosomal large subunit pseudouridine synthase B, RluB (rluB) [4.2.1.70]	684
SAUSA300_1947	phi77 ORF031-like protein	685
SAUSA300_1460	peptidase, M20/M25/M40 family	686
SAUSA300_0794	Toprim domain protein	687
SAUSA300_2515	transcriptional regulator, TetR family	688
SAUSA300_0541	deoxynucleoside kinase family protein [2.7.1.145 2.7.1.-]	689
SAUSA300_1439	conserved hypothetical protein, authentic frameshift	690
SAUSA300_1596	S-adenosylmethionine:tRNA ribosyltransferase-isomerase (queA) [5.-.-.-]	691
pUSA03_0028	resolvase	692
SAUSA300_1452	pyrroline-5-carboxylate reductase (proC) [1.5.1.2]	693
SAUSA300_1284	conserved hypothetical protein	694
SAUSA300_0318	N-acetylmannosamine-6-phosphate 2-epimerase [5.1.3.9]	695
SAUSA300_1906	conserved hypothetical protein	696
SAUSA300_1562	LamB/YcsF family protein	697
SAUSA300_0303	putative lipoprotein	698
SAUSA300_1287	aspartate semialdehyde dehydrogenase (asd) [1.2.1.11]	699
SAUSA300_0231	ABC transporter, substrate-binding protein	700
SAUSA300_0540	HAD-superfamily hydrolase, subfamily IA, variant 1	701
SAUSA300_1946	phiPVL ORF057-like protein, transcriptional activator RinB	702
SAUSA300_1192	glycerol kinase (glpK) [2.7.1.30]	703
SAUSA300_2541	malate:quinone-oxidoreductase (mqo) [1.1.99.16]	704
SAUSA300_2010	2-isopropylmalate synthase (leuA) [2.3.3.13]	705

SAUSA300_1544	GTP-binding protein LepA (lepA)	706
SAUSA300_2576	phosphotransferase system, fructose-specific IIABC component [2.7.1.69]	707
SAUSA300_0663	putative lipoprotein	708
SAUSA300_1311	undecaprenyldiphospho-muramoylpentapeptide beta-N-acetylglucosaminyltransferase (murG) [2.4.1.227]	709
SAUSA300_2439	UTP-glucose-1-phosphate uridylyltransferase (galU) [2.7.7.9]	710
SAUSA300_2589-s4	LPXTG-motif cell wall surface anchor family protein	711
SAUSA300_0681	conserved hypothetical protein	712
SAUSA300_0867	signal peptidase IA (lepB) [3.4.21.89]	713
SAUSA300_0168	conserved hypothetical protein	714
SAUSA300_0243	conserved hypothetical protein	715
SAUSA300_1471	exodeoxyribonuclease VII, small subunit (xseB) [3.1.11.6]	716
SAUSA300_1695	conserved hypothetical protein	717
SAUSA300_1630	primosomal protein DnaI (dnaI)	718
SAUSA300_2085	conserved hypothetical protein	719
SAUSA300_2467	sortase (srtA)	720
SAUSA300_2529	conserved hypothetical protein	721
SAUSA300_2430	staphylococcus tandem lipoprotein	722
SAUSA300_2536	alpha-acetolactate decarboxylase (budA) [4.1.1.5]	723
SAUSA300_0208	putative maltose ABC transporter, ATP-binding protein	724
SAUSA300_0828	5-nucleotidase family protein	725
SAUSA300_1402	phiSLT ORF257-like protein, putative prophage protease	726
SAUSA300_1951	phiPVL ORF052-like protein	727
SAUSA300_1204	conserved hypothetical protein	728
SAUSA300_2489	similar to antibiotic transport-associated protein	729
SAUSA300_2106	putative transcriptional regulator	730
SAUSA300_2024	anti-sigma-B factor, antagonist (rsbV)	731
SAUSA300_1127-s3	chromosome segregation protein SMC (smc)	732
SAUSA300_1254	putative membrane protein	733
pUSA03_0032	oriT nickase (nes)	734
SAUSA300_0809	putative DNA primase	735
SAUSA300_0941	putative ferrichrome ABC transporter	736
SAUSA300_1612	DNA-3-methyladenine glycosidase (tag) [3.2.2.-]	737
SAUSA300_0523	ribosomal protein L1 (rplA)	738
SAUSA300_0151	alcohol dehydrogenase, iron-containing (adhE) [1.1.1.1]	739
SAUSA300_0424.4	psmA1	740
SAUSA300_0578	conserved hypothetical protein	741
SAUSA300_1304	conserved hypothetical protein	742
SAUSA300_0324	conserved hypothetical protein	743
SAUSA300_2311	conserved hypothetical protein	744
SAUSA300_2363	cation efflux family protein	745
SAUSA300_1923	autolysin [3.5.1.28]	746
SAUSA300_2049	phosphomethylpyrimidine kinase (thiD) [2.7.4.7]	747
SAUSA300_1159	transcription termination factor NusA (nusA)	748
SAUSA300_0039	conserved hypothetical protein	749
SAUSA300_2526	dihydroorotate dehydrogenase (pyrD) [1.3.3.1]	750
SAUSA300_0491	cysteine synthase A (cysK) [2.5.1.47]	751
SAUSA300_1962	phiPVL ORF39-like protein	752
SAUSA300_2229	molybdenum ABC transporter, permease protein ModB (modB)	753
SAUSA300_0756	glyceraldehyde-3-phosphate dehydrogenase, type I (gap) [1.2.1.-]	754
SAUSA300_0765	SsrA-binding protein (smpB)	755
SAUSA300_0298	conserved hypothetical protein	756

SAUSA300_1715	riboflavin biosynthesis protein (ribD) [3.5.4.26 1.1.1.193]	757
SAUSA300_1165	riboflavin biosynthesis protein ribF (ribF) [2.7.1.26 2.7.7.2]	758
SAUSA300_1765	lantibiotic epidermin biosynthesis protein EpiC (epiC)	759
SAUSA300_0834	D-isomer specific 2-hydroxyacid dehydrogenase	760
SAUSA300_0910	magnesium transporter (mgtE)	761
SAUSA300_0891	oligopeptide ABC transporter, substrate-binding protein (oppA)	762
SAUSA300_1720	conserved hypothetical protein	763
SAUSA300_0014	DHH subfamily 1 protein	764
SAUSA300_0625	teichoic acid ABC transporter protein (tagG)	765
SAUSA300_0293	conserved hypothetical protein	766
SAUSA300_2239	urease, beta subunit (ureB) [3.5.1.5]	767
SAUSA300_0327	conserved hypothetical protein	768
SAUSA300_2402	conserved hypothetical protein	769
SAUSA300_0668	conserved hypothetical protein	770
SAUSA300_0088	hypothetical protein	771
SAUSA300_2584	preprotein translocase, secA protein	772
SAUSA300_2006	dihydroxy-acid dehydratase (ilvD) [4.2.1.9]	773
SAUSA300_0412	staphylococcus tandem lipoprotein, authentic frameshift	774
SAUSA300_0738	peptide chain release factor 2, programmed frameshift (prfB)	775
SAUSA300_0551	conserved hypothetical protein	776
SAUSA300_0007	conserved hypothetical protein	777
SAUSA300_0770	conserved hypothetical protein	778
SAUSA300_1068	antibacterial protein	779
SAUSA300_0504	pyridoxine biosynthesis protein	780
SAUSA300_0574	phosphomevalonate kinase [2.7.4.2]	781
SAUSA300_0225	putative acyl-CoA acetyltransferase FadA	782
pUSA03_0012	membrane protein TraC (traC)	783
SAUSA300_2251	dehydrogenase family protein	784
SAUSA300_1323	conserved hypothetical protein	785
SAUSA300_1235	guanosine monophosphate reductase (guaC) [1.7.1.7]	786
SAUSA300_1520	conserved hypothetical protein	787
SAUSA300_1636	DNA polymerase I superfamily (polA) [2.7.7.7]	788
SAUSA300_0514	serine acetyltransferase (cysE) [2.3.1.30]	789
SAUSA300_1783	uroporphyrinogen decarboxylase (hemE) [4.1.1.37]	790
SAUSA300_1730	S-adenosylmethionine synthetase (metK) [2.5.1.6]	791
SAUSA300_1549	ComE operon protein 1	792
SAUSA300_1092	uracil permease (pyrP)	793
SAUSA300_2545	choline dehydrogenase (betA) [1.1.99.1]	794
SAUSA300_2241	urease accessory protein UreE (ureE)	795
SAUSA300_0980	putative membrane protein	796
SAUSA300_1731	phosphoenolpyruvate carboxykinase (ATP) (pckA) [4.1.1.49]	797
SAUSA300_0922	membrane protein, TerC family	798
SAUSA300_1986	nitroreductase family protein	799
SAUSA300_1302	ATPase family protein	800
SAUSA300_1136	putative GTP-binding protein	801
SAUSA300_0426	conserved hypothetical protein	802
SAUSA300_0911	transporter, monovalent cation:proton antiporter-2 (CPA2) family protein	803
SAUSA300_0859	NADH-dependent flavin oxidoreductase	804
SAUSA300_0748	conserved hypothetical protein	805
SAUSA300_0434	cystathionine gamma-synthase (metB) [4.4.1.8]	806
SAUSA300_1798	DNA-binding response regulator	807

SAUSA300_1466	2-oxoisovalerate dehydrogenase, E1 component, alpha subunit [1.2.4.1]	808
SAUSA300_2539	aminotransferase	809
SAUSA300_1112	protein phosphatase 2C domain protein	810
SAUSA300_1538	ribosomal protein L11 methyltransferase (prmA) [2.1.1.-]	811
SAUSA300_1885	DNA ligase (ligA) [6.5.1.2]	812
SAUSA300_0825	oxidoreductase, 2-nitropropane dioxygenase family	813
SAUSA300_2644	glucose-inhibited division protein B (gidB) [2.1.-.-]	814
SAUSA300_1576	helicase, RecD/TraA family	815
pUSA03_0013	TraD (traD)	816
SAUSA300_1928	phi77 ORF002-like protein, phage minor structural protein	817
SAUSA300_0087	conserved hypothetical protein, authentic point mutation	818
SAUSA300_2513	conserved hypothetical protein	819
SAUSA300_0929	conserved hypothetical protein	820
SAUSA300_0865	glucose-6-phosphate isomerase (pgi) [5.3.1.9]	821
SAUSA300_1548	ComE operon protein 2	822
SAUSA300_2298	multidrug resistance protein B, drug resistance transporter	823
SAUSA300_0199	conserved hypothetical protein	824
SAUSA300_0532	translation elongation factor G (fusA)	825
SAUSA300_1992	accessory gene regulator protein A (agrA)	826
SAUSA300_0299	conserved hypothetical protein	827
SAUSA300_1852	putative ABC transporter, ATP-binding protein	828
SAUSA300_0301	conserved hypothetical protein	829
SAUSA300_1928-s3	phi77 ORF002-like protein, phage minor structural protein	830
SAUSA300_0454	recombination protein RecR (recR)	831
SAUSA300_1200	glutamine synthetase repressor (glnR)	832
SAUSA300_2325	conserved hypothetical protein	833
SAUSA300_1676	probable transglycosylase (sgtA)	834
SAUSA300_2176	ABC transporter, ATP-binding protein	835
SAUSA300_0125	pyridoxal-dependent decarboxylase	836
SAUSA300_1587	histidyl-tRNA synthetase (hisS) [6.1.1.21]	837
SAUSA300_0706	putative osmoprotectant ABC transporter, ATP-binding protein	838
SAUSA300_2391	glycine betaine/carnitine/choline ABC transporter (opuCc) [3.6.3.32]	839
SAUSA300_1507	glucokinase (glk) [2.7.1.2]	840
SAUSA300_1560	conserved hypothetical protein	841
SAUSA300_0146	conserved hypothetical protein	842
SAUSA300_0478	ribose-phosphate pyrophosphokinase (prs) [2.7.6.1]	843
SAUSA300_0295	conserved hypothetical protein	844
SAUSA300_0994	pyruvate dehydrogenase E1 component, beta subunit (pdhB) [1.2.4.1]	845
SAUSA300_2192	50S ribosomal protein L5 (rplE)	846
SAUSA300_0757	phosphoglycerate kinase (pgk) [2.7.2.3]	847
SAUSA300_0079	putative lipoprotein	848
SAUSA300_1681	acetoin utilization protein AcuC (acuC) [1.2.4.1]	849
SAUSA300_0857	conserved hypothetical protein	850
SAUSA300_1571	O-methyltransferase family protein [2.1.1.-]	851
SAUSA300_0190	indole-3-pyruvate decarboxylase (ipdC) [4.1.1.74]	852
SAUSA300_1627	translation initiation factor IF-3 (infC)	853
SAUSA300_0601	hydrolase, alpha/beta hydrolase fold family [3.4.11.5]	854
SAUSA300_2061	ATP synthase F1, delta subunit (atpH) [3.6.3.14]	855
SAUSA300_1793	conserved hypothetical protein	856
SAUSA300_2038	UDP-N-acetylmuramoyl-tripeptide--D-alanyl-D- alanine ligase (murF) [6.3.2.10]	857

SAUSA300_1269	methicillin resistance protein FemA (femA)	858
SAUSA300_1547	DNA internalization-related competence protein ComEC/Rec2	859
SAUSA300_0919	UDP-N-acetylmuramoylalanyl-D-glutamate--2, 6-diaminopimelate ligase (murE)	860
SAUSA300_2344	uroporphyrin-III C-methyl transferase	861
SAUSA300_0373	conserved hypothetical protein	862
SAUSA300_0481-s1	transcription-repair coupling factor (mfd)	863
SAUSA300_0145	phosphonate ABC transporter, phosphonate-binding protein	864
SAUSA300_2469	L-serine dehydratase, iron-sulfur-dependent, alpha subunit (sdaAA) [4.3.1.17]	865
SAUSA300_0723	conserved hypothetical protein	866
SAUSA300_2187	30S ribosomal protein S5 (rpsE)	867
SAUSA300_0132	glycosyl transferase, group 1 family protein	868
SAUSA300_0137	transcriptional regulator, GntR family	869
SAUSA300_2560	conserved hypothetical protein	870
SAUSA300_1133	tRNA (guanine-N1)-methyltransferase (trmD) [2.1.1.31]	871
SAUSA300_0528-s1	DNA-directed RNA polymerase, beta subunit (rpoC) [2.7.7.6]	872
SAUSA300_1594	preprotein translocase, YajC subunit (yajC)	873
SAUSA300_0424.3	psmA2	874
SAUSA300_2282	putative membrane protein	875
SAUSA300_0603	conserved hypothetical protein	876
SAUSA300_0139	putative tetracycline resistance protein	877
SAUSA300_0284	conserved hypothetical protein	878
SAUSA300_0202	peptide ABC transporter, permease protein	879
SAUSA300_0283-s1	essC protein	880
SAUSA300_1957	phiPVL ORF046-like protein	881
SAUSA300_1371	ATP-dependent DNA helicase RecQ (recQ) [3.6.1.-]	882
SAUSA300_2562	conserved hypothetical protein	883
SAUSA300_0795	putative thioredoxin	884
SAUSA300_2289	conserved hypothetical protein	885
SAUSA300_0004	DNA replication and repair protein recF (recF)	886
SAUSA300_0252	glycosyl transferase, group 2 family protein	887
SAUSA300_2330	conserved hypothetical protein	888
SAUSA300_2378	conserved hypothetical protein	889
SAUSA300_1102	guanylate kinase (gmk) [2.7.4.8]	890
SAUSA300_0275	putative membrane protein	891
SAUSA300_1441	staphylococcal respiratory response protein, SrrB (srrB)	892
SAUSA300_0644	conserved hypothetical protein	893
SAUSA300_0133	putative membrane protein	894
SAUSA300_1393-s1	phiSLT ORF2067-like protein, phage tail tape measure protein	895
SAUSA300_2444	gluconate operon transcriptional repressor (gntR)	896
SAUSA300_0101	staphylococcus tandem lipoprotein	897
SAUSA300_0649	conserved hypothetical protein	898
SAUSA300_1370	cell surface elastin binding protein (ebpS)	899
SAUSA300_1649-s1	DNA polymerase III, alpha subunit (dnaE) [2.7.7.7]	900
SAUSA300_1474	conserved hypothetical protein	901
SAUSA300_0356	conserved hypothetical protein	902
SAUSA300_0410	staphylococcus tandem lipoprotein	903
SAUSA300_1389	phiSLT ORF636-like protein	904
SAUSA300_0311	PfkB family carbohydrate kinase	905
SAUSA300_0938	conserved hypothetical protein	906
SAUSA300_2600	intercellular adhesion protein A (icaA) [2.4.1.-]	907

SAUSA300_0219	putative iron compound A C transporter, iron compound-binding protein	908
SAUSA300_2130	UTP-glucose-1-phosphate uridylyltransferase family protein [2.7.7.23]	909
SAUSA300_2491	1-pyrroline-5-carboxylate dehydrogenase	910
SAUSA300_2296	similar to esterase	911
SAUSA300_2465	ABC transporter, ATP-binding protein	912
SAUSA300_2165	alpha-acetolactate decarboxylase (budA) [4.1.1.5]	913
SAUSA300_0338	glyoxalase family protein	914
SAUSA300_1447	tyrosine recombinase XerD (xerD)	915
SAUSA300_0286	conserved hypothetical protein	916
SAUSA300_2382	conserved hypothetical protein	917
SAUSA300_0272	conserved hypothetical protein	918
SAUSA300_0467	methionyl-tRNA synthetase (metS) [6.1.1.10]	919
SAUSA300_1444	segregation and condensation protein B (scpB)	920
SAUSA300_2083	acetyltransferase, GNAT family	921
SAUSA300_2030	putative membrane protein	922
SAUSA300_2413	conserved hypothetical protein	923
SAUSA300_2163	conserved hypothetical protein	924
SAUSA300_2271	phosphosugar-binding transcriptional regulator	925
SAUSA300_1801	fumarate hydratase, class II (fumC) [4.2.1.2]	926
SAUSA300_0568	integral membrane protein	927
SAUSA300_1332	putative 5-3 exonuclease	928
SAUSA300_0234	putative flavohemoprotein	929
SAUSA300_1528	cytidine deaminase (cdd) [3.5.4.5]	930
SAUSA300_1856	conserved hypothetical protein	931
SAUSA300_1517	endonuclease IV [3.1.21.2]	932
SAUSA300_0128	conserved hypothetical protein	933
SAUSA300_1216	cardiolipin synthetase [2.7.8.-]	934
SAUSA300_1762	lantibiotic epidermin immunity protein F (epiF)	935
pUSA03_0021	transfer complex protein TraL (traL)	936
SAUSA300_2362	2,3-bisphosphoglycerate-dependent phosphoglycerate mutase (gpmA) [5.4.2.1]	937
SAUSA300_0682	ybaK/ebsC protein (ybaK)	938
SAUSA300_0329	putative oxidoreductase	939
SAUSA300_2072	peptide chain release factor 1 (prfA)	940
SAUSA300_2173	tRNA pseudouridine synthase A (truA) [4.2.1.70]	941
SAUSA300_0640	putative membrane protein	942
SAUSA300_0820	cysteine desulfurases, SufS subfamily subfamily (sufS)	943
SAUSA300_0897	tryptophanyl-tRNA synthetase (trpS) [6.1.1.2]	944
SAUSA300_1771	conserved hypothetical protein	945
SAUSA300_0730	GGDEF domain protein	946
SAUSA300_0171	cation efflux family protein	947
SAUSA300_0527	DNA-directed RNA polymerase, beta subunit (rpoB) [2.7.7.6]	948
SAUSA300_1908	conserved hypothetical protein	949
SAUSA300_2092	general stress protein 20U (dps) [1.16.3.1]	950
SAUSA300_0185	arginine biosynthesis bifunctional protein ArgJ (argJ) [2.3.1.35]	951
SAUSA300_2546	glycine betaine aldehyde dehydrogenase (betB) [1.2.1.8]	952
SAUSA300_1145	tyrosine recombinase xerC (xerC)	953
SAUSA300_2184	preprotein translocase, SecY subunit	954
SAUSA300_1615	delta-aminolevulinic acid dehydratase (hemB) [4.2.1.24]	955
SAUSA300_1982	60 kDa chaperonin (groL)	956
SAUSA300_2569	ornithine carbamoyltransferase (arcB) [2.1.3.3]	957
SAUSA300_0425	NADH dehydrogenase I, F subunit	958

SAUSA300_0071	ISSep1-like transposase	959
SAUSA300_1141	endopeptidase resistance gene	960
SAUSA300_0944	1,4-dihydroxy-2-naphthoate octaprenyltransferase (menA) [2.5.-.-]	961
SAUSA300_2586	accessory secretory protein Asp2	962
SAUSA300_1225	aspartate kinase [2.7.2.4]	963
SAUSA300_0184	acetylglutamate kinase (argB) [2.7.2.8]	964
SAUSA300_1393-s2	phiSLT ORF2067-like protein, phage tail tape measure protein	965
SAUSA300_1152	ribosome recycling factor (rrf)	966
SAUSA300_1250	DNA topoisomerase IV, subunit B (parE) [5.99.1.-]	967
SAUSA300_1764	lantibiotic epidermin biosynthesis protein EpiD (epiD) [4.1.1.36]	968
SAUSA300_2004	conserved hypothetical protein	969
SAUSA300_0593	conserved hypothetical protein	970
SAUSA300_0097-s2	conserved hypothetical protein	971
SAUSA300_0691	DNA-binding response regulator SaeR (saeR)	972
SAUSA300_0294	conserved hypothetical protein	973
SAUSA300_1364	GTP-binding protein EngA (engA) [1.1.1.95]	974
SAUSA300_2421	conserved hypothetical protein	975
SAUSA300_2170	endonuclease/methylase LlaGI, degenerate	976
SAUSA300_2079	fructose bisphosphate aldolase (fba) [4.1.2.13]	977
SAUSA300_0661	conserved hypothetical protein	978
SAUSA300_0481-s2	transcription-repair coupling factor (mfd)	979
SAUSA300_2528	conserved hypothetical protein	980
SAUSA300_0292	conserved hypothetical protein	981
SAUSA300_1391	phiSLT ORF527-like protein	982
SAUSA300_2605	histidine biosynthesis bifunctional protein hisIE (hisIE)	983
SAUSA300_1900	manganese-dependent inorganic pyrophosphatase (ppaC) [3.6.1.1]	984
SAUSA300_0887	oligopeptide ABC transporter, permease protein (oppB)	985
SAUSA300_0040	conserved hypothetical protein	986
SAUSA300_2432	hydrolase, MutT/nudix family [3.6.1.-]	987
SAUSA300_0367	single-strand binding protein (ssb)	988
pUSA02_0002	plasmid recombination enzyme	989
SAUSA300_1633	glyceraldehyde-3-phosphate dehydrogenase, type I (gap) [1.2.1.-]	990
pUSA03_0030	conserved hypothetical protein	991
SAUSA300_1009	GTP-binding protein (typA)	992
SAUSA300_2403	putative lipoprotein	993
SAUSA300_0787	3-dehydroquinate dehydratase, type I (aroD) [4.2.1.10]	994
SAUSA300_1740	conserved hypothetical protein	995
SAUSA300_1193	glycerol-3-phosphate dehydrogenase (glpD) [1.1.99.5]	996
SAUSA300_1239	transketolase (tkt) [2.2.1.1]	997
SAUSA300_1592	single-stranded-DNA-specific exonuclease RecJ (recJ) [3.1.-.-]	998
pUSA03_0007	rRNA adenine N-6-methyltransferase (Macrolide-lincosamide-streptogramin B resistance protein) (ermC) [2.1.1.48]	999
SAUSA300_0126	conserved hypothetical protein	1000
SAUSA300_1335	conserved hypothetical protein	1001
SAUSA300_2012	3-isopropylmalate dehydratase, large subunit (leuC) [4.2.1.33]	1002
SAUSA300_1234	30S ribosomal protein S14-2. (rpmN)	1003
SAUSA300_2637	conserved hypothetical protein	1004
SAUSA300_1581	conserved hypothetical protein	1005

SAUSA300_1014-s2	pyruvate carboxylase (pyc) [6.4.1.1]	1006
SAUSA300_0598	putative iron compound ABC transporter, iron compound-binding protein	1007
SAUSA300_1340	recombination protein U (recU)	1008
SAUSA300_0671	ABC transporter, ATP-binding protein, MsbA family	1009
SAUSA300_0287	conserved hypothetical protein	1010
SAUSA300_1519	conserved hypothetical protein	1011
SAUSA300_0221	pyruvate formate-lyase activating enzyme (pflA) [1.97.1.4]	1012
SAUSA300_1637	putative membrane protein	1013
SAUSA300_1260	prephenate dehydrogenase [1.3.1.12]	1014
SAUSA300_1896	prephenate dehydratase (pheA) [4.2.1.51]	1015
SAUSA300_1550	conserved hypothetical protein	1016
SAUSA300_0407	exotoxin	1017
SAUSA300_1987	hydrolase, carbon-nitrogen family	1018
SAUSA300_1516	ABC transporter, ATP-binding protein	1019
SAUSA300_2075	transcription termination factor Rho (rho)	1020
SAUSA300_1310	PAP2 family protein [3.1.3.27]	1021
pUSA03_0003-s2	Isoleucyl-tRNA synthetase, mupirocin resistant protein (ileS) [6.1.1.5]	1022
SAUSA300_0899	putative negative regulator of genetic competence	1023
SAUSA300_0838	D-alanine-activating enzyme/D-alanine-D-alanyl, dltD protein (dltD)	1024
SAUSA300_0870	exonuclease RexA (rexA)	1025
SAUSA300_1108	polypeptide deformylase (def) [3.5.1.88]	1026
SAUSA300_2386	beta-lactamase	1027
SAUSA300_1018	conserved hypothetical protein	1028
SAUSA300_2415	conserved hypothetical protein	1029
SAUSA300_0727	peptidase T (pepT) [3.4.11.4]	1030
SAUSA300_0524	ribosomal protein L10 (rplJ)	1031
SAUSA300_1659	thiol peroxidase (tpx) [1.11.1.-]	1032
SAUSA300_1096	carbamoyl-phosphate synthase, large subunit (carB) [6.3.5.5]	1033
SAUSA300_0796	ABC transporter, ATP-binding protein	1034
SAUSA300_2084	pantothenate kinase (coaA) [2.7.1.33]	1035
SAUSA300_2220	molybdopterin-guanine dinucleotide biosynthesis protein A (mobA)	1036
SAUSA300_0534	amidohydrolase	1037
SAUSA300_2404	conserved hypothetical protein	1038
SAUSA300_2517	amidohydrolase family protein	1039
SAUSA300_0923	serine protease (htrA)	1040
SAUSA300_0355	acetyl-CoA acetyltransferase [2.3.1.9]	1041
SAUSA300_1473	transcription antitermination factor NusB (nusB)	1042
SAUSA300_1114	ribosome small subunit-dependent GTPase A (rsgA)	1043
SAUSA300_1387	phiSLT ORF129-like protein	1044
SAUSA300_1365	30S ribosomal protein S1 (rpsA)	1045
SAUSA300_1185	tRNA-i(6)A37 thiotransferase enzyme MiaB (miaB)	1046
SAUSA300_0196	type I restriction-modification enzyme, R subunit (hsdR)	1047
SAUSA300_1220	DNA-binding response regulator, LuxR family	1048
SAUSA300_1658	conserved hypothetical protein	1049
SAUSA300_0282	conserved hypothetical protein	1050
SAUSA300_1417	phiSLT ORF 175-like protein	1051
SAUSA300_2323	transporter, CorA family (cobI)	1052
SAUSA300_0510	endopeptidase (clpC) [3.4.21.-]	1053
SAUSA300_1363	glycerol-3-phosphate dehydrogenase, NAD-dependent (gpsA) [1.1.1.94 1.1.1.8]	1054
SAUSA300_1320	thymidylate synthase (thyA) [2.1.1.45]	1055

SAUSA300_1105	primosomal protein N` (priA)	1056
SAUSA300_1157	DNA polymerase III, alpha subunit (polC) [2.7.7.7]	1057
SAUSA300_1393	phiSLT ORF2067-like protein, phage tail tape measure protein	1058
SAUSA300_0162	capsular polysaccharide biosynthesis protein Cap5K (cap5K)	1059
SAUSA300_0420	conserved hypothetical protein	1060
SAUSA300_1526	DNA repair protein RecO (recO)	1061
SAUSA300_0445	glutamate synthase, large subunit (gltB) [1.4.1.13]	1062
SAUSA300_0097-s1	conserved hypothetical protein	1063
SAUSA300_1442	staphylococcal respiratory response protein, SrrA (srrA)	1064
SAUSA300_0358	putative 5-methyltetrahydrofolate--homocysteine methyltransferase	1065
SAUSA300_2183	adenylate kinase (adk) [2.7.4.3]	1066
SAUSA300_0550	glycosyl transferase, group 1 family protein	1067
SAUSA300_1477	transposase, IS30 family, authentic frameshift	1068
SAUSA300_1334	putative membrane protein	1069
SAUSA300_0123	siderophore biosynthesis protein, IucC family	1070
SAUSA300_1800	ribosomal large subunit pseudouridine synthase, RluD subfamily	1071
SAUSA300_2275	oxidoreductase, short chain dehydrogenase/reductase family	1072
SAUSA300_1691	glutamyl-aminopeptidase [3.4.11.7]	1073
SAUSA300_1349	glycosyl transferase, group 1 family protein [2.4.1.-]	1074
SAUSA300_2601	intercellular adhesion protein B (icaB)	1075
SAUSA300_1104	phosphopantothenoylcysteine decarboxylase/phosphopantothenate--cysteine ligase (coaBC) [4.1.1.36 6.3.2.5]	1076
SAUSA300_2209	conserved hypothetical protein	1077
SAUSA300_1994	sucrose-6-phosphate hydrolase (scrB) [3.2.1.26]	1078
SAUSA300_2111	phosphoglucosamine mutase (glmM) [5.4.2.10]	1079
SAUSA300_0156	capsular polysaccharide biosynthesis protein Cap5E (cap5E) [5.1.3.- 4.2.1.-]	1080
SAUSA300_2159	aldo/keto reductase family protein [1.1.1.218]	1081
SAUSA300_1780	conserved hypothetical protein	1082
SAUSA300_1913	ABC transporter, ATP-binding protein	1083
SAUSA300_1880	Aspartyl/glutamyl-tRNA amidotransferase subunit B (gatB) [6.3.5.-]	1084
SAUSA300_0085	conserved hypothetical protein	1085
SAUSA300_1403	phiSLT ORF412-like protein, portal protein	1086

Supplementary Table S4: Reactivity values of the 100 most IgG-reactive S. aureus antigen by protein immunoarray. IgG reactivity for each antigen was determined across all patients and samples, with ranking determined by overall mean reactivity, in addition to the presence of reactivity in each patient at each time point (see Methods).

ID	Description	Average	28729 v1	28729 v2	28729 v3	67029 v1	67029 v2	67029 v3	78202 v1	78202 v2	78202 v3	99361 v1	99361 v2	99361 v3	HSS46713 v1	HSS46713 v2	HSS46713 v3	Vander 06 v1	Vander 06 v2	Vander 06 v3	Vander 07 v1	Vander 07 v2	Vander 07 v3
SAUSA300_0113	immunoglobulin G binding protein A precursor	6.370	5.871	5.542	5.932	6.766	6.121	6.443	5.554	4.970	6.285	5.427	6.508	6.162	6.201	6.339	6.199	7.634	7.722	6.768	7.802	6.982	6.538
SAUSA300_0398	superantigen-like protein SSL4	6.212	5.841	5.560	5.863	6.797	6.150	6.505	5.634	5.031	6.491	5.407	6.492	6.085	6.167	6.320	6.074	6.391	6.774	5.905	7.674	6.886	6.412
SAUSA300_0403	superantigen-like protein SSL9	6.210	5.982	5.597	5.989	6.735	6.113	6.466	5.477	4.970	6.415	5.114	6.292	5.889	6.172	6.316	6.094	6.800	6.771	6.193	7.738	6.872	6.411
SAUSA300_2366	gamma-hemolysin component C (hlgC)	6.185	5.624	5.468	5.866	6.595	5.965	6.338	5.337	4.857	6.271	5.225	6.522	6.020	6.004	6.153	5.912	7.304	7.456	6.531	7.347	6.818	6.273
SAUSA300_2579	N-acetylmuramoyl-L-alanine amidase domain protein	6.123	5.944	5.532	5.840	6.388	5.706	6.130	5.611	4.963	6.402	5.031	6.276	5.865	6.251	6.336	6.104	6.592	6.693	5.847	7.749	6.910	6.411
SAUSA300_0883	MAP domain-containing protein; extracellular adherence protein Eap	6.086	5.095	5.106	5.314	6.520	5.998	6.314	5.422	4.909	6.401	5.190	6.262	5.886	6.027	6.211	6.125	7.224	7.232	6.440	7.281	6.670	6.169
SAUSA300_2364	IgG-binding protein SBI (sbi)	6.039	5.753	5.427	5.772	6.402	5.820	6.234	5.272	4.536	6.096	4.898	6.207	5.793	6.239	6.322	6.077	6.956	7.448	6.147	7.216	6.358	5.852
SAUSA300_1029	Iron-regulated surface determinant Protein A (IsdA)	6.032	5.688	5.341	5.661	6.350	5.819	6.079	5.325	4.723	6.018	5.005	6.231	5.865	5.968	5.856	5.896	7.080	7.324	6.243	7.382	6.613	6.197
SAUSA300_1028	Iron-regulated surface determinant Protein B (IsdB)	6.028	5.709	5.422	5.811	6.290	5.695	6.025	5.531	4.988	6.352	5.144	6.377	5.868	6.067	6.145	5.964	6.469	6.937	5.702	7.279	6.671	6.147
SAUSA300_1920	chemotaxis-inhibiting protein CHIPS (chs)	5.947	5.628	5.282	5.596	6.543	5.872	6.219	5.096	4.503	5.840	4.955	6.232	5.833	5.874	6.125	5.819	6.893	7.043	6.104	7.084	6.422	5.928
SAUSA300_1382	Panton-Valentine leukocidin, LukS-PV (lukS-PV)	5.857	5.372	5.273	5.768	6.197	5.769	6.188	5.496	4.944	6.413	4.800	6.423	5.808	5.806	6.121	5.743	6.119	6.918	5.687	6.481	6.267	5.414
SAUSA300_2367	gamma-hemolysin component B (hlgB)	5.854	5.245	5.116	5.499	6.155	5.712	6.076	5.336	4.884	6.182	5.026	6.258	5.863	5.601	5.800	5.646	6.409	7.122	5.879	6.835	6.501	5.799
SAUSA300_1768	leukotoxin LukD (lukD)	5.850	5.416	5.207	5.573	6.158	5.746	6.128	5.500	4.999	6.356	5.115	6.397	5.970	5.815	5.954	5.814	5.852	6.783	5.411	6.698	6.315	5.648
SAUSA300_1917	map protein, programmed frameshift (map)	5.828	5.166	4.961	5.295	6.172	5.801	5.975	5.256	4.632	6.045	4.867	6.157	5.817	6.054	5.900	5.844	7.005	7.196	6.094	6.602	6.122	5.431
SAUSA300_0651	CHAP domain family	5.802	4.230	4.071	3.766	6.428	5.811	6.191	5.156	4.529	6.010	4.776	6.007	5.593	6.021	5.962	5.901	7.315	7.307	6.459	7.348	6.834	6.121
SAUSA300_0951	V8 protease (sspA) [3.4.21.19]	5.800	4.470	4.777	5.057	5.963	5.492	5.801	5.441	4.777	6.195	4.765	6.240	5.701	5.645	5.675	5.585	7.066	6.933	6.022	7.312	6.787	6.089
SAUSA300_0408	heme uptake protein IsdB	5.792	5.377	4.944	4.988	6.560	5.853	6.359	5.246	4.752	6.113	5.021	6.275	5.773	5.599	5.642	5.200	5.966	6.071	5.469	7.399	6.732	6.287
SAUSA300_0438	CHAP domain family	5.766	4.207	3.900	3.719	6.528	5.883	6.225	5.008	4.492	6.002	4.901	6.144	5.589	6.012	6.126	5.953	7.069	7.022	6.320	7.192	6.604	6.185
SAUSA300_0409	putative exported protein	5.763	5.093	4.896	5.419	6.590	5.778	6.272	4.730	4.201	5.817	4.853	6.228	5.705	6.005	6.218	5.943	6.387	6.960	5.607	6.766	6.210	5.333
SAUSA300_0693	putative lipoprotein	5.747	5.150	4.935	5.714	5.769	5.446	5.760	5.128	4.802	6.121	5.361	6.472	6.042	6.164	6.218	6.050	6.395	7.079	5.917	6.076	6.018	4.071
SAUSA300_0214	Sugar phosphate isomerase/epimerase PFAM family PF07582	5.730	4.909	4.895	5.253	6.037	5.674	5.967	5.149	4.662	6.074	4.860	6.173	5.624	5.537	5.691	5.535	6.537	6.986	5.924	6.690	6.364	5.793
SAUSA300_2506	immunodominant staphylococcal antigen A precursor (isaA)	5.655	4.982	4.599	5.191	6.383	5.749	6.121	4.627	4.214	5.431	4.878	6.094	5.693	5.973	5.935	5.841	6.413	6.309	5.145	6.968	6.379	5.819
SAUSA300_1058	alpha-hemolysin precursor	5.599	5.358	5.112	5.618	6.215	5.757	6.032	5.256	4.828	6.100	5.072	6.225	5.837	5.827	5.926	5.882	3.890	4.641	4.090	7.342	6.524	6.048
SAUSA300_0548-s2	sdrE protein (sdrE)	5.592	4.758	4.452	4.642	6.870	6.090	6.535	5.726	5.055	6.538	4.014	5.335	4.724	5.583	5.940	5.192	6.644	6.676	6.148	5.908	5.568	5.026
SAUSA300_0776	thermonuclease precursor (nuc) [3.1.31.1]	5.558	4.000	3.813	3.941	6.124	5.635	5.883	5.077	4.504	5.831	4.920	6.098	5.705	5.554	5.608	5.573	6.772	6.906	6.045	6.867	6.225	5.639
SAUSA300_0547-s2	sdrD protein (sdrD)	5.498	4.913	4.695	5.022	6.749	6.035	6.455	5.352	4.700	6.104	3.868	5.191	4.642	5.563	5.728	5.359	6.386	6.416	5.833	5.868	5.622	4.949
SAUSA300_2572	zinc metalloproteinase aureolysin (aur) [3.4.24.29]	5.494	3.505	3.541	3.679	6.640	6.060	6.382	4.911	4.337	6.117	5.349	6.511	6.145	5.932	5.824	5.604	6.676	6.523	5.486	5.926	5.568	4.662
SAUSA300_0370	SEP staphylococcal enterotoxin	5.445	5.103	4.857	5.184	6.301	5.858	6.199	4.917	4.351	5.766	5.183	6.213	5.890	5.849	6.011	5.849	4.417	6.157	2.651	6.284	5.910	5.401
SAUSA300_1030	iron transport associated domain protein	5.418	4.930	5.023	5.469	6.449	5.848	6.174	5.121	4.637	6.082	4.487	5.507	5.105	5.708	5.894	5.676	6.514	7.034	6.026	4.406	4.514	3.184
SAUSA300_1476	acetyl-CoA carboxylase, biotin carboxyl carrier protein (accB)	5.411	5.099	4.693	4.899	6.438	5.763	6.121	4.613	3.979	5.306	4.372	5.688	5.153	5.592	5.725	5.440	5.973	6.392	5.293	6.280	5.695	5.111
SAUSA300_1922	staphylokinase precursor (sak)	5.342	5.157	4.806	5.279	6.212	5.544	5.922	4.228	3.795	5.461	4.448	5.711	5.197	4.157	4.191	3.282	6.823	6.921	6.103	6.868	6.276	5.798
SAUSA300_0862	glycerophosphoryl diester phosphodiesterase (glpQ) [3.1.4.46]	5.268	4.757	4.393	4.830	5.859	5.411	5.693	3.958	3.210	4.682	4.297	5.504	4.979	5.180	5.144	5.050	6.544	6.669	5.697	6.745	6.220	5.804
SAUSA300_1055	fibrinogen-binding protein (efb)	5.217	4.956	4.653	4.957	5.933	5.504	5.705	4.415	3.897	5.293	4.277	5.249	4.838	5.383	5.469	5.331	6.086	6.727	5.378	5.587	5.285	4.631
SAUSA300_1481	putative membrane protein	5.172	4.055	3.864	3.983	5.535	5.194	5.377	4.890	4.305	5.757	4.218	5.517	5.176	5.967	5.938	5.810	6.163	6.734	5.205	5.377	5.234	4.315
SAUSA300_0955-s1	autolysin (atl) [3.5.1.28]	5.170	3.664	3.408	3.277	6.138	5.676	5.905	4.485	3.940	5.382	4.666	5.812	5.410	5.539	5.499	5.341	6.077	6.232	5.424	5.994	5.681	5.024
SAUSA300_0774	secretory extracellular matrix and plasma binding protein (empbp)	5.170	5.319	5.022	5.336	6.315	5.603	6.133	5.202	4.643	6.154	4.495	5.786	5.403	5.905	5.832	5.514	3.517	4.856	3.217	5.214	4.893	4.206
SAUSA300_0950	cysteine protease precursor (sspB) [3.4.22.48]	5.136	1.555	1.596	2.397	6.188	5.768	5.978	5.197	4.379	6.035	3.839	5.457	4.836	5.375	5.378	5.274	6.761	6.828	5.925	6.999	6.458	5.638
SAUSA300_1381	Panton-Valentine leukocidin, LukF-PV (lukF-PV)	5.127	4.998	4.821	5.115	4.405	4.982	5.395	5.182	4.647	5.896	4.728	6.080	5.617	5.161	5.149	5.271	3.834	5.911	4.121	5.722	5.656	4.979
SAUSA300_2441	fibronectin binding protein A (fnbA)	5.124	5.087	4.886	5.196	6.065	5.400	5.828	4.562	3.827	5.536	4.586	5.922	5.421	5.557	5.840	5.367	4.309	4.231	4.126	5.719	5.371	4.766
SAUSA300_2136	iron compound ABC transporter, iron compound-binding protein	5.048	5.340	5.123	5.502	6.172	5.695	5.986	4.443	3.706	4.950	4.369	5.444	5.012	5.345	5.143	4.914	2.871	6.744	3.692	5.168	5.733	4.648
SAUSA300_2440	fibronectin binding protein B (fnbB)	5.013	4.795	4.632	4.875	5.860	5.346	5.698	4.616	3.919	5.408	4.627	5.854	5.414	5.301	5.581	5.266	4.076	4.111	3.684	5.812	5.532	4.859
SAUSA300_0242	sorbitol dehydrogenase (gutB) [1.1.1.14]	5.003	4.166	4.249	4.472	5.354	5.098	5.362	4.623	4.235	5.397	4.250	5.623	5.083	5.038	4.992	4.827	5.216	6.305	4.953	5.583	5.555	4.680
SAUSA300_1985	serine-aspartate repeat family protein, SdrH (sdrH)	4.977	4.687	4.494	4.866	6.632	6.047	6.383	5.138	4.441	5.889	3.154	4.412	3.920	6.012	5.997	5.878	6.671	6.687	6.151	2.528	2.080	2.455
SAUSA300_0279	membrane protein YhgE, phage infection protein (PIP) family EsaA	4.976	2.502	2.441	2.421	6.802	6.150	6.528	5.039	4.319	5.936	4.428	5.630	5.159	5.581	5.510	5.302	7.034	7.009	6.368	3.744	3.671	2.923
SAUSA300_0708	histidinol-phosphate aminotransferase (hisC) [2.6.1.9]	4.936	4.394	4.484	4.769	5.331	5.169	5.546	5.017	4.640	5.894	4.621	5.944	5.423	5.257	5.200	5.081	3.590	5.269	3.519	4.960	5.217	4.329
SAUSA300_0399	superantigen-like protein SSL5	4.931	4.618	4.385	4.861	5.787	5.202	5.569	4.254	3.770	5.250	3.850	5.146	4.661	4.936	5.087	4.588	4.985	4.900	4.702	6.181	5.588	5.234
SAUSA300_0955-s2	autolysin (atl) [3.5.1.28]	4.927	3.129	2.903	2.920	5.703	5.295	5.499	4.478	3.814	5.544	4.078	5.364	4.998	4.977	4.985	4.967	5.846	5.838	5.232	6.487	6.040	5.377
SAUSA300_0395	superantigen-like protein SSL1	4.861	5.928	5.672	5.957	6.506	6.112	6.462	5.584	5.011	6.395	4.752	5.952	6.156	6.255	6.300	6.138	2.180	2.268	1.906	2.284	2.318	1.952
SAUSA300_1031	heme ABC transporter permease	4.843	3.503	3.471	3.863	6.090	5.623	5.867	4.183	3.771	5.322	4.578	5.705	5.306	4.924	5.031	4.434	5.622	5.548	5.215	5.057	4.833	3.764
SAUSA300_1512	penicillin-binding protein 3 (pbp3)	4.726	3.782	3.763	3.858	6.180	5.597	5.863	3.146	2.629	4.087	3.760	4.658	4.315	4.884	4.825	4.383	5.502	5.427	5.089	6.324	5.897	5.269
SAUSA300_0546	sdrC protein (sdrC)	4.696	2.544	2.375	2.397	6.332	5.780	6.058	5.114	4.547	6.040	3.406	4.510	3.868	4.981	5.469	4.902	5.653	5.586	5.207	4.913	4.664	4.273
SAUSA300_0404	exotoxin	4.682	4.089	3.769	4.198	5.323	4.902	5.108	3.902	3.293	4.954	3.575	4.569	4.117	4.741	5.001	4.811	5.100	5.646	4.560	6.148	5.396	5.113
SAUSA300_0401	exotoxin	4.633	5.210	4.980	5.138	6.506	6.039	6.365	4.173	3.751	5.190	4.585	5.607	5.598	6.176	6.106	6.040	1.998	2.383	1.770	3.564	3.303	2.817
SAUSA300_2146	alcohol dehydrogenase, zinc-containing	4.607	4.102	4.279	4.657	4.808	4.408	5.234	4.701	4.225	5.630	3.663	5.510	4.705	4.874	5.091	4.790	3.700	5.537	3.922	4.311	4.913	3.682
SAUSA300_2039	D-alanine--D-alanine ligase (ddl) [6.3.2.4]	4.598	4.179	3.751	3.769	5.009	4.683	4.880	3.782	3.134	4.390	4.297	5.366	5.051	5.029	4.846	4.818	4.750	5.716	4.161	5.478	5.044	4.428
SAUSA300_2219	molybdenum cofactor biosynthesis protein A (moaA)	4.585	2.424	2.367	2.029	5.705	5.033	5.436	3.604	3.125	4.671	3.676	4.797	4.260	5.331	5.468	5.245	5.635	5.564	5.183	6.008	5.654	5.066
SAUSA300_2441-s2	fibronectin binding protein A (fnbA)	4.566	4.660	4.472	4.779	5.081	4.362	4.986	3.968	3.275	4.974	4.353	5.502	5.017	5.054	5.402	4.962	3.776	3.755	3.507	5.042	4.813	4.159
SAUSA300_1890	staphopain A	4.522	3.232	3.076	3.353	5.671	5.039	5.396	4.221	3.725	5.222	3.141	4.299	3.696	5.008	5.078	4.864	4.806	4.773	4.355	5.854	5.347	4.810
SAUSA300_0307	5-nucleotidase, lipoprotein e(P4) family	4.513	4.117	3.812	4.228	4.963	4.296	4.746	3.811	3.199	4.690	3.528	4.700	4.283	4.309	4.482	4.137	5.649	5.895	5.030	5.617	4.896	4.383
SAUSA300_0589	aldo/keto reductase family protein	4.510	3.990	3.884	4.139	5.789	5.419	5.660	4.652	4.049	5.491	3.691	4.667	4.290	4.975	5.047	4.888	2.797	2.787	2.533	5.739	5.346	4.871
SAUSA300_0602	conserved hypothetical protein	4.413	3.985	3.863	3.977	4.929	4.715	4.538	4.511	4.098	5.319	2.492	3.151	2.967	5.580	5.680	5.634	5.420	5.412	5.087	4.747	4.223	2.347
SAUSA300_2573	immunodominant antigen B (isaB)	4.388	3.789	3.613	3.567	6.178	5.619	5.853	4.566	4.135	5.626	3.188	3.996	3.626	5.711	5.708	5.216	4.028	3.964	3.922	3.872	3.505	2.470
SAUSA300_0320	triacylglycerol lipase precursor [3.1.1.3]	4.384	4.081	3.796	4.241	5.600	5.021	5.392	3.844	3.593	5.251	3.608	4.619	4.128	5.249	5.373	5.014	3.786	4.102	3.807	4.196	3.983	3.368
SAUSA300_2565	clumping factor B (clfB)	4.334	2.465	2.264	2.376	6.462	5.840	6.169	4.933	4.322	5.798	3.657	4.707	4.190	3.673	4.953	3.488	3.926	4.016	3.782	5.184	4.684	4.120
SAUSA300_1975	LukA	4.266	4.175	3.888	3.879	4.948	4.363	5.024	3.458	2.922	4.263	3.085	4.638	4.014	4.721	4.398	3.887	3.401	4.788	2.913	6.095	5.686	5.043
SAUSA300_1061	putative exotoxin 3	4.163	3.508	3.405	3.456	5.093	4.852	5.093	4.444	3.979	5.272	3.110	4.044	3.738	4.550	4.715	4.344	3.385	4.035	3.318	4.878	4.354	3.854
SAUSA300_2441-s1	fibronectin binding protein A (fnbA)	4.115	3.266	3.154	3.428	6.167	5.576	5.989	3.978	3.493	4.931	3.344	4.587	4.081	5.261	5.151	4.274	1.633	2.008	1.440	5.186	5.127	4.351
SAUSA300_0573	diphosphomevalonate decarboxylase (mvaD) [4.1.1.33]	4.080	3.091	3.111	3.381	4.423	4.092	4.426	3.642	3.123	4.526	3.286	4.513	4.004	3.952	3.988	3.965	4.658	5.247	4.149	4.939	4.924	4.235
SAUSA300_0038	cassette chromosome recombinase A (ccrA)	4.041	3.744	3.585	3.672	4.454	3.852	4.449	3.829	3.147	4.754	2.858	3.944	3.494	4.784	4.755	4.316	4.337	5.357	3.658	4.485	4.034	3.348
SAUSA300_0547-s1	sdrD protein (sdrD)	4.030	4.092	3.940	4.382	6.383	5.790	6.117	3.435	2.748	4.438	1.250	1.704	1.452	4.280	4.604	4.193	4.208	4.277	4.096	4.778	4.480	3.976
SAUSA300_0025	5-nucleotidase family protein	3.970	2.746	2.597	2.753	4.988	4.648	4.923	3.559	2.818	4.401	3.276	4.274	3.971	3.644	3.521	3.167	4.910	4.951	4.441	4.987	4.672	4.126
SAUSA300_2380	conserved hypothetical protein	3.926	3.649	3.294	3.290	4.941	4.329	4.705	3.282	2.724	3.950	2.937	4.135	3.505	4.489	4.528	4.087	4.337	4.837	3.760	4.227	3.985	3.448
SAUSA300_1186	conserved hypothetical protein	3.778	3.442	3.075	3.122	4.811	4.175	4.435	3.216	2.637	3.892	2.815	3.942	3.380	4.424	4.417	3.933	4.127	4.642	3.593	4.022	3.888	3.360
SAUSA300_2476	phosphotransferase system, glucose-specific IIABC component (ptsG)	3.660	3.269	3.005	3.080	4.004	3.418	3.774	3.303	2.897	4.021	2.709	3.755	3.246	4.422	4.514	4.291	3.455	4.463	2.873	4.659	4.143	3.569
SAUSA300_0396	exotoxin 7 (set7)	3.657	2.987	2.955	3.249	4.590	4.160	4.499	2.917	2.435	3.761	2.822	3.339	3.183	4.470	4.369	4.235	3.539	3.899	3.233	4.476	4.145	3.533
SAUSA300_1988	delta-hemolysin precursor	3.617	1.090	1.106	1.461	5.995	5.311	5.681	2.449	1.854	3.030	1.048	1.872	1.435	5.327	5.380	4.747	5.985	6.141	5.259	3.848	3.840	3.104
pUSA03_0034	conserved hypothetical protein	3.551	1.241	1.358	1.085	4.783	4.031	4.442	2.312	1.931	3.480	2.532	3.680	3.083	4.524	4.552	4.348	4.505	4.388	4.204	4.976	4.902	4.208
SAUSA300_1613	putative abrB protein	3.548	2.642	2.774	3.028	3.944	3.595	3.990	3.446	3.136	4.166	2.879	4.109	3.569	3.368	3.576	3.257	3.607	4.857	3.446	3.854	4.092	3.169
SAUSA300_1664	septation ring formation regulator EzrA (ezrA)	3.509	3.580	3.338	3.526	5.158	4.276	4.693	3.952	3.057	4.542	2.127	3.073	3.760	3.988	4.276	3.960	3.476	3.296	3.519	2.548	1.881	1.661
SAUSA300_1059	putative exotoxin 1	3.483	3.131	2.835	2.852	4.064	3.538	3.866	3.140	2.652	4.171	2.711	3.825	3.267	3.698	3.930	3.617	3.635	4.114	3.543	3.998	3.507	3.050
SAUSA300_2530	transcriptional regulator, TetR family	3.400	3.185	3.144	3.514	4.842	4.068	4.645	2.764	2.400	3.860	2.381	3.385	2.969	3.629	3.655	3.028	2.537	2.534	2.668	4.439	4.099	3.649
SAUSA300_0229	putative acyl-CoA transferase FadX	3.319	2.452	2.269	2.561	3.966	3.479	3.880	2.397	2.089	3.072	2.080	2.942	2.938	3.813	3.883	3.670	4.449	4.900	3.612	4.266	3.689	3.292
SAUSA300_0734	putative comf operon protein 1	3.315	2.708	2.709	2.962	3.638	3.518	3.899	3.793	3.564	4.614	2.966	4.182	3.727	3.818	3.567	3.633	1.589	3.448	1.611	3.206	3.677	2.777
SAUSA300_2226	molybdenum cofactor biosynthesis protein B (moaB)	3.218	3.028	2.867	2.909	4.294	3.671	4.120	2.662	2.099	3.613	2.287	2.916	2.500	4.022	4.101	3.751	3.730	4.605	3.333	2.774	2.321	1.982
SAUSA300_0351	putative membrane protein	3.170	3.518	3.415	3.718	4.435	3.905	4.357	2.728	2.412	3.749	1.592	1.997	1.479	4.066	4.268	3.869	1.537	1.484	1.676	4.616	4.181	3.574
SAUSA300_1346	putative DnaQ family exonuclease/DinG family helicase	3.155	2.528	2.268	2.444	4.532	4.038	4.331	3.026	1.939	3.462	2.489	3.405	3.098	3.416	3.440	3.255	2.806	2.848	2.519	3.848	3.477	3.093
SAUSA300_0441	acetyltransferase, GNAT family	3.125	1.423	1.589	1.670	4.092	3.794	4.128	2.767	2.285	3.781	2.364	2.493	2.373	3.927	4.072	3.959	4.003	4.043	3.545	3.348	3.278	2.698
SAUSA300_0955	autolysin (atl) [3.5.1.28]	3.074	1.967	1.836	1.770	4.272	3.576	4.065	2.143	1.462	3.086	2.216	3.482	3.045	3.575	3.546	3.232	3.695	3.677	3.108	4.207	3.725	2.868
SAUSA300_0830	conserved hypothetical protein	3.061	2.812	2.414	2.462	4.183	3.563	3.818	2.498	1.982	3.104	2.116	3.205	2.543	3.792	3.807	3.302	3.338	3.994	2.828	3.069	2.929	2.525
SAUSA300_2589	LPXTG-motif cell wall surface anchor family protein	3.048	1.084	1.041	1.078	4.368	3.864	4.254	3.279	2.572	4.079	2.173	3.219	2.939	4.221	4.054	3.835	4.084	4.535	3.405	2.188	2.319	1.409
SAUSA300_2285	aldose 1-epimerase (galM) [5.1.3.3]	2.944	2.516	2.753	3.098	3.070	2.732	3.685	3.232	2.788	4.225	2.142	3.940	3.107	3.332	3.646	3.134	1.508	3.649	2.006	2.299	3.117	1.855
SAUSA300_1895	nitric oxide synthase oxygenase	2.938	2.014	1.990	2.125	3.367	3.023	3.444	2.510	2.041	3.372	2.059	3.201	2.655	3.233	2.987	2.782	3.107	3.777	2.676	3.985	4.040	3.303
SAUSA300_2641	conserved hypothetical protein	2.908	2.625	2.236	2.352	3.974	3.404	3.653	2.386	1.859	2.933	1.895	2.997	2.340	3.627	3.679	3.197	3.164	3.884	2.725	2.925	2.840	2.384
SAUSA300_1249	conserved hypothetical protein	2.740	2.539	2.508	2.844	4.034	3.314	3.830	2.231	1.765	3.282	1.823	2.740	2.390	2.932	2.952	2.378	1.827	1.798	1.971	3.783	3.479	3.112
SAUSA300_0364	GTP-binding protein YchF (ychF)	2.644	2.374	2.031	1.952	3.429	2.988	3.361	1.853	1.204	2.536	1.618	2.442	2.249	3.365	3.129	3.009	2.687	3.854	2.304	3.509	3.227	2.401
SAUSA300_1354	conserved hypothetical protein	2.560	1.482	1.560	1.743	2.864	2.601	2.940	2.452	1.963	3.268	1.726	2.836	2.346	2.590	2.547	2.355	2.819	3.413	2.463	3.401	3.543	2.843
SAUSA300_1330	threonine dehydratase (ilvA) [4.3.1.19]	2.457	1.720	1.948	2.283	2.357	2.003	2.841	2.446	2.056	3.414	1.287	3.157	2.352	2.599	2.850	2.342	3.492	4.134	3.568	1.483	2.247	1.020
SAUSA300_0082	conserved hypothetical protein	2.379	2.088	1.744	1.819	3.470	2.918	3.170	1.961	1.479	2.462	1.452	2.407	1.801	3.145	3.113	2.695	2.771	3.306	2.147	2.142	2.119	1.741
SAUSA300_1879	conserved hypothetical protein	2.334	1.464	1.680	1.837	2.693	2.370	2.729	2.138	1.687	2.750	1.505	2.678	2.232	2.271	2.468	2.094	3.219	3.634	2.474	2.405	2.730	1.966
SAUSA300_1095	carbamoyl-phosphate synthase, small subunit (carA) [6.3.5.5]	2.189	1.318	1.515	1.676	2.517	2.268	2.676	2.038	1.811	2.777	1.602	2.662	2.254	2.170	2.280	1.874	2.106	3.395	2.017	2.421	2.638	1.950

Supplementary Table S5. Protein subsets based on differential IgG reactivity between infection groups. Proteins identified in each group of the Venn Diagram in Figure 1B. The cutoff for each group was an average reactivity for that infection group of 1 or above.

Supplementary Table S6: *S. aureus* patient isolates sequencing data. Patient infecting *S. aureus* isolates were collected at the time of presentation and sequenced as described in Methods. Key: -: negative; +: positive; ACME: arginine catabolic mobile element; CC: clonal complex; *pvl*: Panton-Valentine leukocidin; SCC*mec:* staphylococcal chromosome cassette *mec.*
[a] Strains were assigned to clonal complexes using the MLST mapping feature at the Ridom SpaServer (http://spa.ridom.de/mlst.shtml), or a combination of typing methods[1].

Patient ID	Kreiswith/Ridom *spa* type	*spa* motif	MRSA	SCC*mec*	*pvl*	ACME	CC[a]
SSTI 1	59/t211	YHGGFMBQBLO	+	4	+	+	CC8
SSTI 2	1/t008	YHGFMBQBLO	+	4	+	+	CC8
SSTI 3	1/t008	YHGFMBQBLO	-	-	+	-	CC8
SSTI 4	t665	WGKKAKAO	+	4	+	-	CC30
PJI 1	7/t064	YHGCMBQBLO	+	4	-	-	CC8
PHO 1	536/t046	XKAKAOMQQQ	-	-	-	-	CC30
PHO 2	42/t065	A2AKBEMBKB	-	-	-	-	CC45

Supplementary References:

1 Mendes, R. E. *et al.* Characterization of methicillin-resistant *Staphylococcus aureus* strains recovered from a phase IV clinical trial for linezolid versus vancomycin for treatment of nosocomial pneumonia. *J Clin Microbiol* **50**, 3694-3702, doi:10.1128/JCM.02024-12 (2012).
